# Synthesis of New Fused Heterocyclic 2-Quinolones and 3-Alkanonyl-4-Hydroxy-2-Quinolones

**DOI:** 10.3390/molecules24203782

**Published:** 2019-10-21

**Authors:** Ashraf A. Aly, Alaa A. Hassan, Nasr K. Mohamed, Lamiaa E. Abd El-Haleem, Stefan Bräse, Mika Polamo, Martin Nieger, Alan B. Brown

**Affiliations:** 1 Chemistry Department, Faculty of Science, Minia University, El-Minia 61519, Egypt; alaahassan2001@mu.edu.eg (A.A.H.); nasrmohamed603@yahoo.com (N.K.M.);; 2 Institute of Organic Chemistry, Karlsruhe Institute of Technology, 76131 Karlsruhe, Germany; 3 Institute of Toxicology and Genetics, Karlsruhe Institute of Technology, 76344 Eggenstein-Leopoldshafen, Germany; 4 Department of Chemistry, University of Helsinki, P.O. Box 55 (A. I. Virtasen aukio I), 00014 Helsinki, Finland; mika.polamo@helsinki.fi (M.P.); martin.nieger@helsinki.fi (M.N.); 5 Chemistry Program, Florida Institute of Technology, Melbourne, FL 32901, USA; abrown@fit.edu

**Keywords:** 6,7-Disubstituted-4-hydroxy-quinoline-2-ones, 2,3-dichloropyrazine, ANRORC process bis-pyrazinofuro-quinoline, 1,4-diphenylbutane-1,4-diones, NMR, X-ray

## Abstract

Herein, we report the synthesis of 5,12-dihydropyrazino[2,3-*c*:5,6-*c*′]difuro[2,3-*c*:4,5-*c*′]-diquinoline-6,14(*5H*,12*H*)diones, 2-(4-hydroxy-2-oxo-1,2-dihydroquinolin-3-yl)-1,4-diphenyl- butane-1,4-diones and 4-(benzo-[*d*]oxazol-2-yl)-3-hydroxy-1*H*-[4,5]oxazolo[3,2-*a*]pyridine-1-one. The new candidates were synthesized and identified by different spectroscopic techniques, and X-ray crystallography.

## 1. Instruction

**Figure 1:**
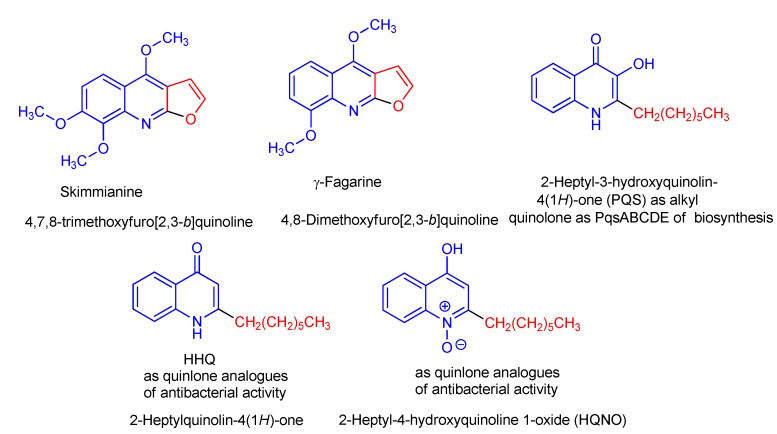
The structures of Skimmianine, γ-Fagarine, an example of QS of PqsABCDE biosynthesis and two examples of quinolone analogues as antibacterial reagents.

Furoquinolones are an interesting class of 2-quinolones and representative examples of them as naturally occurring compounds are shown in compounds such as *Skimmianine* and γ-Fagarine (Figure 1), both compounds that have been found to have anti-cancer activity [1,2]. It was reported that furopyrazine scaffold was functionalized with an amino- and a carboxy-terminus resulting in a conformationally restricted dipeptidomimetic scaffold [3].

Alkyl quinolones (AQs) are a species-specific class of quorum-sensing molecule that have been described in *P. aeruginosa* [4,5] and related bacteria including *P. putida* and *Burkholderia spp*. [6]. More than 55 distinct AQs (i.e., an example is shown in Figure 1 and assigned as PQS) are produced through the PqsABCDE (Figure 1) biosynthetic pathway in *P. aeruginosa*, with the majority of the diversity arising from unsaturation, different alkyl chain lengths, and modification of the ring-substituted nitrogen [6,7]. An insight into the evolutionary basis of AQ diversity has emerged from *Burkholderia thailandensis* where AQ analogues (i.e., two examples assigned as HHQ and HQNP and are shown in Figure 1) are shown to act synergistically to inhibit bacterial growth [8,9].

Quinolones have been also developed for clinical use in humans [10]. These antibiotics exert their effect by inhibition of two type II topoisomerase enzymes, DNA gyrase and topoisomerase IV [11]. DNA topoisomerases are found in both eukaryotic and prokaryotic cells and are a target for chemotherapeutic intervention in anti-bacterial and anti-cancer therapies [12]. In a recent publication [13], the synthesis of the Zwitter-ionic 4-hydroxycoumarin derivatives was reported, through a unique reaction of 4-hydroxycoumarins with *p*-benzoquinone and pyridine in aqueous acetone.

**Figure 2:**
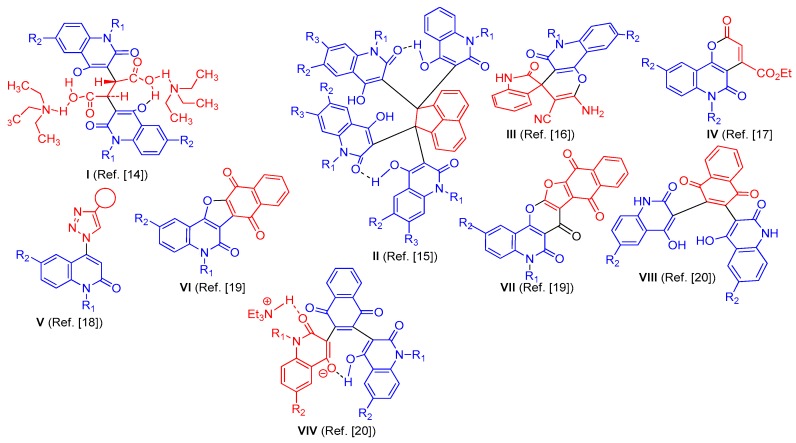
Structures of compounds that previously reported (Reproduced with permission from [14,15,16,17,18,19,20]).

Figure 2 indicates what we previously synthesized of 4-hydroxy-2-quinolone. An example, 2,3-bis-(4-hydroxy-2-oxo-1,2-dihydroquinolin-3-yl)succinic acid derivatives **I**, were obtained by one-pot reaction of one equivalent of aromatic amines with two equivalents of diethyl malonate in diphenyl ether and catalyzed with triethylamine [14]. On reacting four equivalents of 4-hydroxyquinolin-2(1*H*)-ones with one equivalent of acenaphthoquinone in absolute ethanol, containing catalytic Et_3_N, the reaction gave acenaphthylene-1,1,2,2-tetrayl-tetrakis- (4-hydroxyquinolin-2(1*H*)-ones) (**II**, [15]. We also reported that quinoline-2,4-diones reacted with 2-(2-oxo-1,2-dihydroindol-3-ylidene)malononitrile in pyridine to give spiro(indoline-3,4′-pyrano- [3,2-*c*]quinoline)-3′-carbonitriles (**III**, [16]. The same target materials of 2-quinolones reacted with diethyl acetylenedicarboxylate in absolute ethanol, containing catalytic triethylamine, to give pyrano [3,2-*c*]quinoline-4-carboxylates (**IV**, [17]. We have recently reported that a class of 1,2,3-triazoles derived by 2-quinolone (**V**, [18]) has been synthesized, via Cu-catalyzed [3 + 2]cycloadditions (Meldal–Sharpless ‘click’-reactions) of 4-azidoquinolin-2(1*H*)-ones with ethyl propiolate [18]. We also synthesized fused naphthofuro[3,2-*c*]quinoline-6,7,12-triones **VI**, and pyrano[3,2-*c*]quinoline-6,7,8,13-tetraones, **VII** that have shown potential as ERK inhibitors [19]. Whereas syntheses of bis(6-substituted-4-hydroxy-2-oxo-1,2-dihydro-quinolin-3-yl)-naphthalene-1,4-diones **VIII** and substituted *N*-(alkyl)bis-quinolinone triethyl-ammonium salts **VIV**, were explored as candidates for extracellular signal-regulated kinases 1/2 (ERK1/2) having antineoplastic activity [20].

The aforementioned interesting pharmaceutical and biological activities of 4-hydroxy-2-quinolones make them valuable in drug research and development. Hence, many publications have recently dealt with their synthetic analogous and the synthesis of their heteroannelated derivatives. Consequently, we have found that it is of importance to shed new light on these interesting heterocycles. Accordingly, the reactivity of 1,6-disubstituted-4-hydroxy- quinolinones **1a**–**f** was tested towards 2,3-dichloropyrazine (**2**) and (*E*)-dibenzoylethene (**4**).

## 2. Results and Discussion

**Scheme 1:**
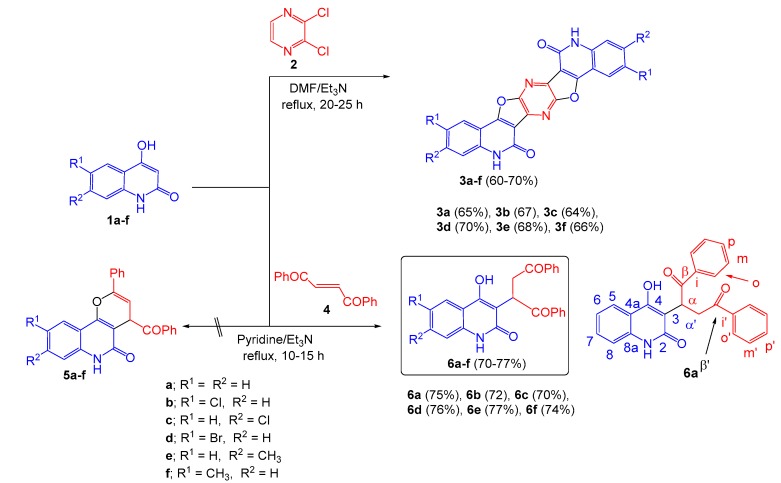
Reactions of 2-quinolinones **1a**–**f** with 2,3-dichloropyrazine (**2**) and 1,2-(*E*)-dibenzoylethene (**4**).

Upon mixing equivalent amounts of 2,3-dichloropyrazine (**2**) and 6,7-disubstituted-4-hydroxy- quinolin-2-ones **1a**–**f**, followed by refluxing in dimethylformamide (DMF) and catalyzed by triethylamine (Et_3_N), **3a**–**f** were obtained as single products (Scheme 1). Structure elucidation of compounds **3a**–**f** was carried out by infrared (IR), ^1^H-nuclear magnetic resonance (NMR), ^13^C-NMR and mass spectrometry, as well as elemental analyses.

**Figure 3:**
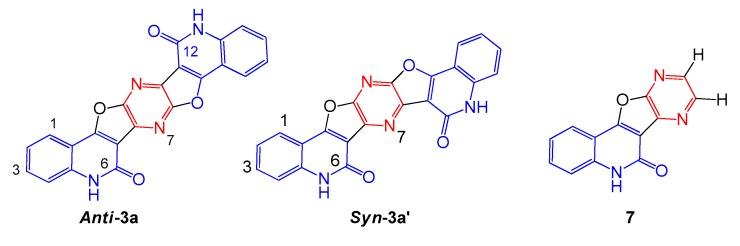
Alternative structures of compound **3a** [14,15,16,17,18,19,20] (Reproduced with permission from [14,15,16,17,18,19,20]).

The reaction products were identified as pyrazino[2′,3′:4,5]furo[3,2-*c*]quinolin-6(5*H*)-ones **3a**–**f**. The IR spectra showed absorption for C=N at ν = 1630–1600 cm^−1^. Besides the NH stretching appeared as broad peaks at ν = 3320–3260 cm^−1^. As for example, the elemental analysis and mass spectra proved its molecular formula of **3a** as C_22_H_10_N_4_O_4**,**_ which indicated addition of two moles of **1a** to two moles of **2** accompanied with elimination of four moles of HCl. The expected structure **7** was ruled out, since ^1^H-NMR spectrum did not show the expected azomethine protons (Figure 3). Either the *syn*-structure **3a**′ or the *anti-*form has to show symmetric carbon signals in ^13^C-NMR spectrum. Most assigned carbons are the two carbonyl and C=N carbons which appeared at δ = 167.1, 165.2 and 160.8, 160.2 ppm. In Spartan 18: geometries program [21] optimized at the 6-31G* level with B3LYP the energy difference between calculated *Anti*-**3a**: and *Syn*-**3a**’ shows that *Anti-***3a** is more stable by 2.029 kcal/mol.

**Scheme 2:**
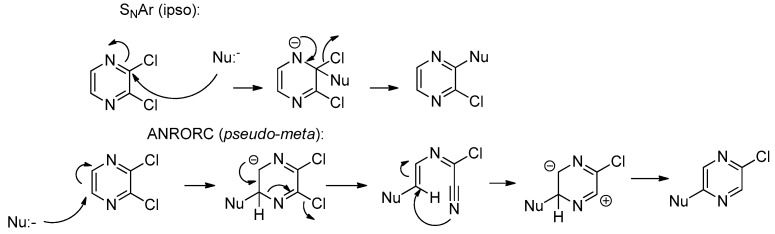
S_N_Ar versus ANRORC substitutions on **2.**

**Scheme 3:**
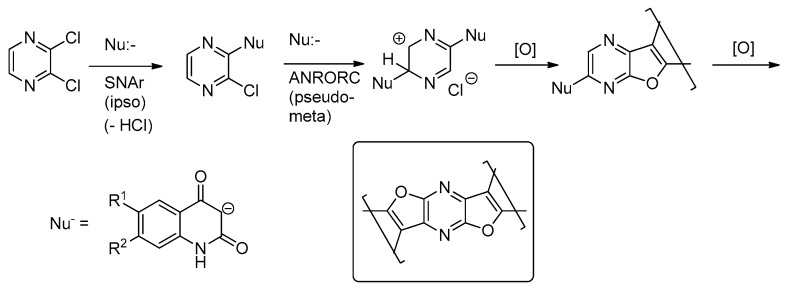
Proposed rationale for formation of **3.**

Compounds **3** are the products of reaction between one molecule of 2,3-dichloropyrazpine (**2**) and two molecules of a 4-hydroxy-2-quinolinone (**1**); substituents on **3** correspond to those on **1** in the obvious way. The reaction results in replacement of both chlorines and both hydrogens of **2**, by either an α-carbon or a *pseudo*-phenolic oxygen of **1.** Both positions involved on **1** are formally nucleophilic, although the α-carbon normally reacts first *(cf.* reaction of **1** with **4** to give **6**). We are unaware of any direct reaction of **2** with four nucleophiles; in systems where the chlorines and hydrogens of **2** are all replaced, the reaction of C-5 and C-6 (the hydrogen-bearing carbons) involves reaction with an oxidant (e.g., a molecular halogen), sometimes followed by an organometallic coupling [22,23]. On the other hand, pyrazines bearing leaving groups readily undergo displacement of those leaving groups, by either S_N_Ar or Addition of the Nucleophile, Ring Opening, and Ring Closure (ANRORC) mechanisms [24,25,26]. If the pyrazine is activated by an electrophile, the nucleophile can be as weak as *o*-phenylenediamine [27]. If **2** does not undergo four-fold nucleophilic substitution, the likeliest scenario would seem to be *two-*fold displacement followed by two-fold oxidative cyclization, presumably by air. If the chlorides are the leaving groups, only one can undergo *ipso* substitution: the other nucleophile must attack the other side of the ring. The S_N_Ar process can give *ipso* substitution only, but the ANRORC process can proceed at the *pseudo-meta* position (Scheme 2). We are therefore led to propose the mechanism for formation of **3,** shown in Scheme 3. Here “Nu:^-^“ is **1**, attacking via its α-carbon. If one substitution gave *ipso* displacement and the other gave *pseudo-meta* displacement, one would expect the observed *anti* regiochemistry: the order of the two substitutions would not matter.

**Figure 4:**
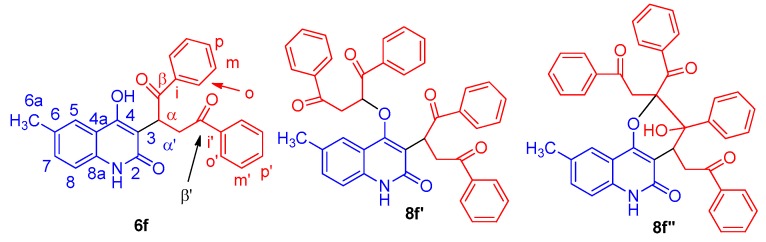
Different suggested structures of **6f**, **8f’** and **8f’’.**

**Figure 5:**
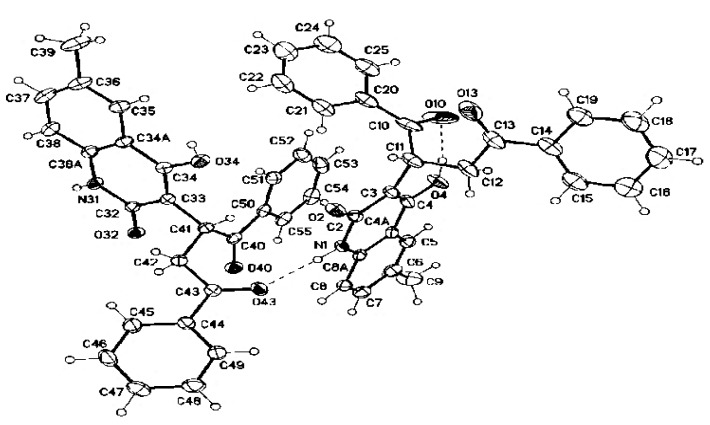
Molecular structure of the dimer of compound **6f.**

Afterward, we investigated the reactions of (*E*)-dibenzoylethene (**4**) and **1a**–**f** under the same conditions mentioned above in pyridine/Et_3_N (Scheme 1). The structure elucidation depends intensively on NMR spectra. For example, the ^1^H spectrum of **6f** consists of a 1H singlet at δ_H_ = 11.35 and a broad signal at δ_H_ = 10.77; in the aromatic region, two sets of resonances from monosubstituted phenyl rings and a three-spin system from the quinoline; and a three-spin system and a 3H methyl singlet upfield. The integrals require that there be two phenyl rings and one quinolone and, consequently, they rule out the alternative structures **8f**′ and **8f**′′ (Figure 4). The NMR correlations of **6f** are detailed in full in Table S1 (see Supplementary Material). The structure of compound **6f** was finally proved by X-ray structure analysis as shown in Figure 5.

**Scheme 4:**
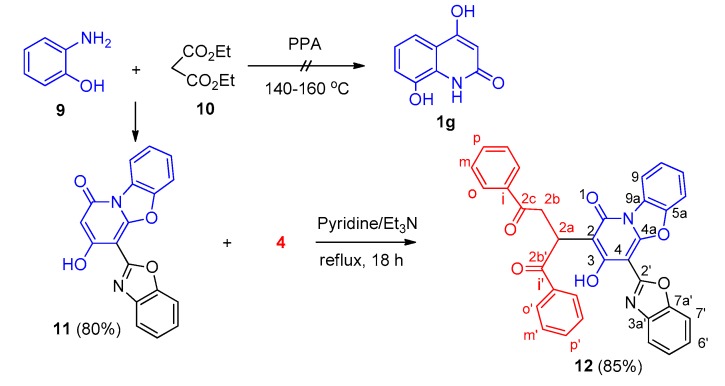
Formation of compound **11** and its reaction with compound **4**.

Surprisingly, on attempting to prepare 4-hydroxy-2-quinolone **1g** from 2-hydroxyaniline (**9**) and diethyl malonate (**10**) in polyphosphoric acid (PPA) according to the procedure described in reference [28], compound **11** was obtained in 80% yield (Scheme 4). Similarly, reaction of **11** with **4** under the same conditions produced compound **12** in 85% yield.

**Scheme 5:**
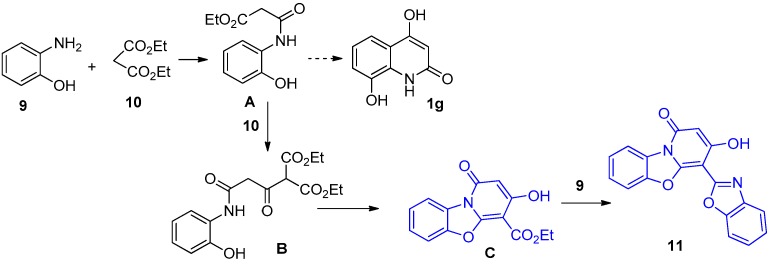
Proposed rationale for formation of **11.**

We propose that compound **11** forms as shown in Scheme 5. The reaction of **9** with **10** presumably starts with *N*-acylation to form intermediate **A**. The expected formation of **1g** would then involve ring closure in the fashion of a Friedel-Crafts acylation. The pathway leading to **11**, on the other hand, would require Claisen condensation between **A** and a second molecule of **10** to form intermediate **B**. One of the pendant esters of **B** would then react intramolecularly with the NH and OH groups, to form the benzoxazole subunit of intermediate **C**. Reaction of the remaining ester group of **C** with a second molecule of **9** would then form the other benzoxazole unit of **11**.

**Figure 6:**
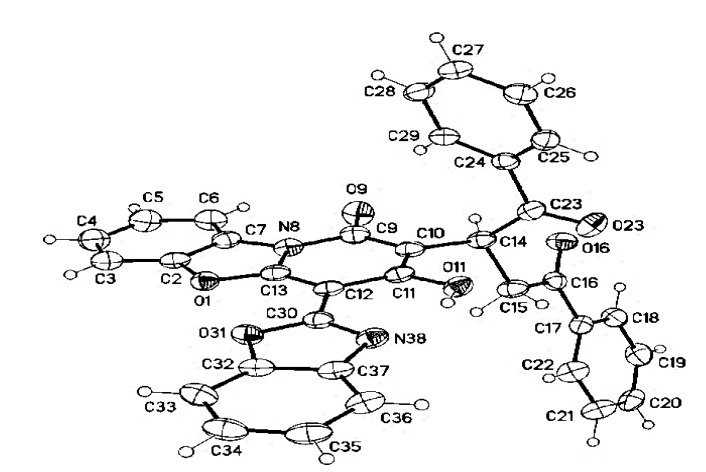
Molecular structure of compound **12** assigned as 2-(4-hydroxy-6-methyl-2-oxo-1,2-dihydroquinolin-3-yl)-1,4-diphenylbutane-1,4-dione.

The NMR spectroscopic data of compound **12** (detailed in full in Table S2; see Supplementary Materials) show a broad OH, two sets of phenyl signals, and eight other proton signals, although most of the benzoxazole signals cannot be solved fully. The protons on *sp*^3^ carbons are distinctive at δ = 5.55, 4.36, and 2.85 ppm; the latter two are assigned as H-2b based on their geminal coupling constant of 17 Hz. The attached carbons appear at δ = 39.5 (C-2a) and 37.35 ppm (C-2b). The C-2a gives Heteronuclear Multiple Bond Correlation (HMBC) correlation with a carbon at δ = 156.92 ppm, which could be either C-1 or C-3, and is assigned based on chemical shift as C-1. Correlations within the benzoyl groups are straightforward. The structure of compound **12** was confirmed by X-ray crystallography (Figure 6).

## 3. Experimental

### 3.1. Material and Methods

Melting points were taken in open capillaries on a Gallenkamp melting point apparatus (Weiss-Gallenkamp, Loughborough, UK) and are uncorrected. The IR spectra were recorded from potassium bromide disks with a Fourier Transform Infrared (FT-IR) device (Mettler-Toledo GmbH, Giessen, Germany), Minia University. Elemental analyses were carried out at the Perkin-Elmer out with Elementar 306 (Perkin-Elmer, Walluf, Germany). NMR data were recorded on Bruker AM 400 or AV400 spectrometers (Bruker, Karlsruhe, Germany), at 400 MHz for ^1^H and 100 MHz for ^13^C. Chemical shifts were reported in ppm from tetramethylsilane using solvent resonance in DMSO-*d_6_* solutions as the internal standard. Coupling constants are stated in Hz. Correlations were established using ^1^H-^1^H COSY, and ^1^H-^13^C and ^1^H-^15^N heteronuclear single quantum coherence (HSQC) and HMBC experiments. Mass spectra were recorded on a Finnigan MAT 312 instrument Fab 70 eV (Thermo Fisher, Bremen, Germany), Institute of Organic Chemistry, Karlsruhe University, Karlsruhe, Germany. Thin Layer Chromatography (TLC) was performed on analytical Merck 9385 silica aluminum sheets (Kieselgel 60) with Pf_254_ indicator; TLCs were viewed at λ _max_ = 254 nm. Elemental analyses for C, H, N were carried out with Elementar 306.

### 3.2. Starting Materials

1,6-Disubstituted-quinoline-2,4-(1*H*,3*H*)-diones **1a**–**f** were prepared according to the literature [28,29]. 2,3-Dichloropyrazine (**2**) and (*E*)-1,2-dibenzoylethene (**4**) (Aldrich, Munich, Germany) were used as received.

#### 3.2.1. Reaction of **1a**–**f** with 2,3-Dichloropyrazine (**2**)

A suspension of 1,6-disubstituted quinoline-2,4-(1*H*,3*H*)-diones **1a**–**f** (2 mmol) in 10 mL dimethylformamide (DMF) was added to a solution of (*E*)-1,4-diphenylbut-2-ene-1,4-dione (**2**, 0.148 g, 1 mmol) in 15 mL DMF and 0.5 mL of triethylamine. The reaction mixture was gently refluxed for 20–25 h, until the reactants had disappeared (monitored by TLC). The resulting precipitates of **3a**–**f** which were obtained cold were filtered off and dried. The precipitates were recrystallized from the stated solvents.

#### 3.2.2. 5,12-Dihydropyrazino[2,3-*c*:5,6-*c*′]difuro[2,3-*c*:4,5-*c*′]diquinoline-6,14(5*H*,12*H*)-Dione (**3a**)

Buff crystals (DMF/EtOH), yield: 0.256 g (65%), mp = 330–332 °C; IR (KBr): ν = 3220 (NH), 1660 (CO), 1606 cm^−1^ (Ar-C=N); ^1^H-NMR (400 MHz, DMSO-*d_6_*): δ = 12.11 (s, 2H, NH), 7.89–7.85 (m, 2H, Ar-H), 7.52–7.48 (m, 2H, Ar-H), 7.35–7.32 (m, 2H, Ar-H), 7.23–7.19 ppm (m, 2H, Ar-H); ^13^C-NMR (100 MHz, DMSO-*d_6_*): δ = 165.2 (C=O), 160.2 (C=N), 153.4, 139.4, 138.4, 130.7 (Ar-C), 125.9, 128.4, 122.8, 115.7 (Ar-CH), 109.5 ppm (Ar-C); MS (Fab, 70 eV, %): *m/z* = 394 (M^+^, 27). *Anal*. *Calcd. for* C_22_H_10_N_4_O_4_ (394.35): C, 67.01; H, 2.56; N, 14.21. *Found*: C, 66.90; H, 2.70; N, 14.30.

#### 3.2.3. 2,10-Dichloro-5,12-Dihydropyrazino[2,3-*c*:5,6-*c*′]difuro[2,3-*c*:4,5-*c*′]-Diquinoline-6,14(5*H*,12*H*)-Dione (**3b**)

Buff crystals (DMF/EtOH), yield: 0.310 g (67%), mp = 320–322 °C, IR (KBr): ν = 3210 (NH), 1665 (CO), 1612 cm^−1^ (Ar-C=N); ^1^H-NMR (400 MHz, DMSO-*d_6_*): δ = 12.07 (s, 2H, 2NH), 7.98 (d, 2H, *J* = 0.7 Hz, Ar-2CH), 7.38–7.20 ppm (m, 4H, Ar-H); ^13^C-NMR (100 MHz, DMSO-*d_6_*): δ = 167.2 (C=O), 160.2 (C=N), 154.4, 139.0, 138.2 (Ar-C), 128.1, 126.9, 125.4, 120.0 (Ar-2H), 118.0, 109.4 ppm (Ar-C); MS (Fab, 70 eV,%): *m/z* = 464/468 (M^+^, 60/30), 443 (25), 371 (22), 137 (50). *Anal*. *Calcd. for* C_22_H_8_Cl_2_N_4_O_4_ (463.23): C, 57.04; H, 1.74; N, 12.10. *Found*: C, 57.20; H, 1.70; N, 12.20.

#### 3.2.4. 3,11-Dichloro-5,12-Dihydropyrazino[2,3-*c*:5,6-*c*′]difuro[2,3-*c*:4,5-*c*′]diquinoline-6,14(5*H*,12*H*)-Dione (**3c**)

Buff crystals (DMF), yield: 0.295 g (64%), mp = 350–352 °C; IR (KBr): ν = 3230 (NH), 1663 (CO), 1620 cm^−1^ (Ar-C=N); ^1^H-NMR (400 MHz, DMSO-*d_6_*): δ = 12.11 (s, 2H, 2NH), 7.95 (d, 2H, *J* = 1.2 Hz, Ar-2CH), 7.45–7.41 ppm (m, 4H, Ar-H); ^13^C-NMR (100 MHz, DMSO-*d_6_*)_:_ δ = 167.0 (C=O), 160.4 (C=N), 152.0, 138.7, 138.0, 130.6 (Ar-C), 127.0, 124.9, 122.2 (Ar-CH), 120.0, 108.2 ppm (Ar-C); MS (Fab, 70 eV,%): *m/z* = 464/468 (M^+1^, 32/64), 462 (36), 443 (32), 371 (24), 137 (54). *Anal*. *Calcd. for* C_22_H_8_Cl_2_N_4_O_4_ (463.23): C, 57.04; H, 1.74; N, 12.10. *Found*: C, 57.00; H, 1.80; N, 12.15.

#### 3.2.5. 2,10-Dibromo-5,12-Dihydropyrazino[2,3-*c*:5,6-*c*′]difuro[2,3-*c*:4,5-*c*′]diquinoline-6,14(5H,12H)-Dione (**3d**)

Pale yellow crystals (DMF), yield: 0.390 g (70%), mp = 310–312 °C; IR (KBr): ν = 3230 (NH), 1667 (CO), 1610 cm^−1^ (Ar-C=N); ^1^H-NMR (400 MHz, DMSO-*d_6_*): δ = 12.43 (s, 2H, 2NH), 8.01–7.96 (m, 2H, Ar-CH), 7.75–7.72 (m, 2H, Ar-H), 7.38–7.36 ppm (m, 2H, Ar-H); ^13^C-NMR (100 MHz, DMSO-*d_6_*): δ = 166.8 (C=O), 160.4 (C=N), 150.8, 141.0, 135.5, 131.4 (Ar-C), 128.6, 127.8, 124.2 (Ar-CH), 118.2, 103.0 ppm (Ar-C); MS (Fab, 70 eV, %): *m/z* = 552/556 (M^+^, 34/58), 552 (100), 371 (22), 137 (50). *Anal*. *Calcd. for* C_22_H_8_B_2_N_4_O_4_ (552.14): C, 47.86; H, 1.46; N, 10.15. *Found*: C, 47.90; H, 1.56; N, 10.25.

#### 3.2.6. 5,12-Dihydro-3,11-Dimethylpyrazino[2,3-*c*:5,6-*c*′]difuro[2,3-*c*:4,5-*c*′]diquinoline-6,14(5H,12H)-Dione (**3e**)

Brown crystals (DMF/CH_3_OH), yield: 0.280 g (66%), mp = 276–278 °C (decomp.), IR (KBr): ν = 3222 (NH), 1660 (CO), 1620 cm^−1^ (Ar-C=N); ^1^H-NMR (400 MHz, DMSO-*d_6_*): δ = 12.15 (s, 2H, 2NH), 7.92 (d, 2H, *J* = 0.8 Hz, Ar-H), 7.45–7.30 (m, 4H, Ar-H), 2.16 ppm (s, 6H, 2CH_3_); ^13^C-NMR (100 MHz, DMSO-*d_6_*): δ = 166.8 (C=O), 159.8 (C=N), 150.0 (C-O), 138.0, 137.4, 130.0, 128.2 (Ar-C), 127.8, 127.2 (Ar-CH), 125.5, 110.4 (Ar-C), 20.4 ppm (CH_3_); MS (Fab, 70 eV, %): *m/z* 422 = (M^+^, 28), 406 (24), 370 (20), 137 (48). *Anal*. *Calcd. for* C_24_H_14_Cl_2_N_4_O_4_ (422.40): C, 68.24; H, 3.34; N, 13.26. *Found*: C, 68.30; H, 3.45; N, 13.35.

#### 3.2.7. 5,12-Dihydro-2,10-Dimethylpyrazino[2,3-*c*:5,6-*c*′]difuro[2,3-*c*:4,5-*c*′]diquinoline-6,14(5H,12H)-Dione (**3f**)

Brown crystals (DMF/CH_3_CN), yield: 0.290 g (68%), mp 300–302 °C; IR (KBr): ν = 3225 (NH), 1665 (CO), 1630 cm^−1^ (Ar-C=N); ^1^H-NMR (400 MHz, DMSO-*d_6_*): δ = 12.20 (s, 1H, 2NH), 7.84 (d, 2H, *J* = 1.2 Hz, Ar-2CH), 7.42–7.35 (m, 4H, Ar-H), 2.18 ppm (s, 6H, 2CH_3_); ^13^C-NMR (100 MHz, DMSO-*d_6_*): δ = 166.2 (C=O), 160.0 (C=N), 152.7 (C-O), 139.6 (Ar-CH_3_), 135.4, 130.4, 128.6 (Ar-C), 128.0, 127.0, 126.2 (Ar-CH), 110.4 (Ar-C), 20.2 ppm (CH_3_); MS (Fab, 70 eV, %): *m/z* = 422 (M^+^, 28), 406 (24), 370 (20), 137 (48). *Anal*. *Calcd. for* C_24_H_14_Cl_2_N_4_O_4_ (422.40): C, 68.24; H, 3.34; N, 13.26. *Found*: C, 68.20; H, 3.50; N, 13.32.

### 3.3. Reaction of **1a**–**f** with (E)-1,2-Dibenzoylethene *(**4**)*

A mixture of **1a**–**f** (1 mmol) and (*E*)-1,2-dibenzoylethene (**4**) (0.246 g, 1 mmol) in pyridine (50 mL) and 0.5 mL of triethylamine, was gently refluxed for 10–15 h, until the reactants had disappeared (monitored by TLC). The resulting precipitates of **6a**–**f**, which obtained on cold were filtered off and dried. The precipitates were recrystallized from the stated solvents.

#### 3.3.1. 2-(4-Hydroxy-2-oxo-1,2-Dihydroquinolin-3-yl)-1,4-Diphenylbutane-1,4-Dione (**6a**)

Yellow crystals (DMF/EtOH), yield: 0.300 g (75%), mp = 325–327 °C, IR (KBr): ν = 3220 (NH), 3023 (Ar-CH), 3000 (Aliph-CH), 1698, 1673 (CO), 1631 (Ar-C=N), 1590 cm^−1^ (C=C); ^1^H-NMR (400 MHz, DMSO-*d_6_*): δ = 8.01 (d, *J* = 7.5 Hz, 2H; H-*o’*), 7.92 (d, *J* = 8.0 Hz, 1H; H-5), 7.83 (d, *J* = 7.5 Hz, 2H; H-*o*), 7.65 (t, *J* = 7.3 Hz, 1H; H-*p’*), 7.55 (“t”, *J* = 7.6 Hz, 2H; H-*m’*), 7.48 (t, *J* = 7.5 Hz, 1H; H-*p*), 7.46 (“t”, *J* = 7.8 Hz, 1H; H-7), 7.38 (“t”, *J* = 7.5 Hz, 2H; H-*m*), 7.23 (d, *J* = 8.1 Hz, 1H; H-8), 7.14 (“t”, *J* = 7.6 Hz, 1H; H-6), 5.47 (dd, *J* = 9.6, 2.4 Hz, 1H; H-α), 4.21 (dd, *J* = 17.3, 9.8 Hz, 1H; H-α’), 2.82 ppm (dd, *J* = 17.2, 2.5 Hz, 1H; H-α’). ^15^N-NMR: 142.95 ppm (N-1). ^13^C-NMR (100 MHz, DMSO-*d_6_*): δ = 198.48 (C-β), 198.31 (C-β’), 162.36 (C-2), 158.85 (C-4), 137.81, 137.01, 136.66 (C-*i, i’,* 8a), 132.91 (C-*p’*), 132.29 (C-*p*), 130.44 (C-7), 128.63 (C-*m’*), 128.21 (C-*m*), 127.83 (C-*o’*), 127.36 (C-*o*), 122.65 (C-5), 121.08 (C-6), 115.17, 115.06 (C-4a, 8), 111.47 (C-3), 40.41 (C-α), 37.44 ppm (C-α’). MS (Fab, 70 eV, %): *m/z* = 397 (M^+^, 100). *Anal*. *Calcd. for* C_25_H_19_NO_4_ (397.43): C, 75.55; H, 4.82; N, 3.52. *Found*: C, 75.70; H, 4.90; N, 3.62.

#### 3.3.2. 2-(6-Chloro-4-Hydroxy-2-oxo-1,2-Dihydroquinolin-3-yl)-1,4-Diphenylbutane-1,4-Dione (**6b**)

Pale yellow crystals (DMF/CH_3_CN), yield: 0.310 g (72%), mp = 315–317 °C, IR (KBr): ν = 3230 (NH), 3010 (Ar-CH), 2803 (Aliph-CH), 1693, 1644 (CO), 1604 cm^−1^ (Ar-C=N); ^1^H-NMR (400 MHz, DMSO-*d_6_*): δ = 12.30 (s, 1H, OH), 11.50 (s, 1H, NH), 8.00 (d, 1H, *J* = 7.6 Hz, Ar-H), 7.90 (d, 1H, *J* = 7.98 Hz, Ar-H), 7.82 (d, 1H, *J* = 8.0 Hz, Ar-H), 7.54–7.50 (m, 5H, Ar-H), 7.30 (t, 2H, *J* = 7.8 Hz, Ar-H), 7.24–7.10 (m, 3H, Ar-H), 5.50 (dd, 1H, *J* = 10, 2.3 Hz, CH-α), 4.18 (dd, 1H, *J* = 17.0, 9.9 Hz, CH_2_-α), 2.80 (dd, 1H, *J* = 17.0, 2.6 Hz, CH_2_-α’); ^13^C-NMR (100 MHz, DMSO-*d_6_*): δ = 198.5, 198.3, 164.0 (CO), 158.0 (Ar-C), 136.8 (Ar-3C), 132.6, 132.1 (Ar-CH-*p*), 130.3 (Ar-2CH), 128.5, 128.0 (Ar-2CH-*m*), 127.8, 127.6 (Ar-2CH-*o*), 123.0 (Ar-CH-5), 120.0 (Ar-C-Cl), 115.2, 110.0 (Ar-C), 40.5 (CH_2_), 37.2 ppm (CH). MS (Fab, 70 eV, %): *m/z* = 431/433 (M^+2^, 23), 413 (17), 309 (14), 148 (37), 104 (100). *Anal*. *Calcd. for* C_25_H_18_ClNO_4_ (431.87): C, 69.53; H, 4.20; N, 3.24. *Found*: C, 69.62; H, 4.30; N, 3.32.

#### 3.3.3. 2-(7-Chloro-4-Hydroxy-2-oxo-1,2-Dihydroquinolin-3-yl)-1,4-Diphenylbutane-1,4-Dione (**6c**)

White crystals (DMF/CH_3_CN), yield: 0.302 g (70%), mp = 340–342 °C, IR (KBr): ν = 3225 (NH), 3030 (Ar-CH), 2820 (Aliph-CH), 1690, 1650 (CO), 1612 cm^−1^ (Ar-C=N); ^1^H-NMR (400 MHz, DMSO-*d_6_*): δ = 12.10 (s, 1H, OH), 11.40 (s, 1H, NH), 7.98 (d, 1H, *J* = 7.8 Hz, Ar-H), 7.80 (d, 1H, *J* = 8.0 Hz, Ar-H), 7.60–7.50 (m, 6H, Ar-H), 7.20 (t, 2H, *J* = 7.8 Hz, Ar-H), 7.18–7.12 (m, 3H, Ar-H), 5.45 (dd, 1H, *J* = 10, 2.3 Hz, CH-α), 4.20 (dd, 1H, *J* = 17.0, 9.9 Hz, CH_2_-α), 2.85 ppm (dd, 1H, *J* = 17.0, 2.6 Hz, CH_2_-α’); ^13^C-NMR (100 MHz, DMSO-*d_6_*): δ = 198.4, 198.1, 162.4 (CO), 157.8 (Ar-C), 138.7, 137.0, 136.6 (Ar-C), 134.9, 132.9 (Ar-CH-*p*), 132.4 (Ar-2CH), 129.8, 128.9 (Ar-2CH-*m*), 128.6, 128.5 (Ar-2CH-*o*), 122.6 (Ar-CH-5), 119.5 (Ar-C-Cl), 121.3, 114.3 (Ar-C), 40.6 (CH_2_), 39.0 ppm (CH). MS (Fab, 70 eV, %): *m/z* = 431/433 (36/60), 413 (17), 309 (14), 148 (37), 104 (100). *Anal*. *Calcd. for* C_25_H_18_ClNO_4_ (431.87): C, 69.53; H, 4.20; N, 3.24. *Found*: C, 69.70; H, 4.15; N, 3.28.

#### 3.3.4. 2-(6-Bromo-4-Hydroxy-2-oxo-1,2-Dihydroquinolin-3-yl)-1,4-Diphenylbutane-1,4-Dione (**6d**)

Pale yellow crystals (DMF/CH_3_OH), yield: 0.365 g (76%), mp = 348–350 °C, IR (KBr): ν = 3230 (NH), 3010 (Ar-CH), 2803 (Aliph-CH), 1693, 1644 (CO), 1604 cm^−1^ (Ar-C=N); ^1^H-NMR (400 MHz, DMSO-*d_6_*): δ = 12.30 (s, 1H, OH), 11.50 (s, 1H, NH), 8.00 (d, 1H, *J* = 7.6 Hz, Ar-H), 7.90 (d, 1H, *J* = 7.98 Hz, Ar-H), 7.82 (d, 1H, *J* = 8.0 Hz, Ar-H), 7.54–7.50 (m, 5H, Ar-H), 7.30 (t, 2H, *J* = 7.8 Hz, Ar-H), 7.24–7.10 (m, 3H, Ar-H), 5.50 (dd, 1H, *J* = 10, 2.3 Hz, CH-α), 4.18 (dd, 1H, *J* = 17.0, 9.9 Hz, CH_2_-α), 2.80 ppm (dd, 1H, *J* = 17.0, 2.6 Hz, CH_2_-α’); ^13^C-NMR (100 MHz, DMSO-*d_6_*): δ = 198.2, 197.6, 165.0 (CO), 158.2 (Ar-C), 138.0, 137.4, 133.0 (Ar-C), 132.4, 132.0 (Ar-CH-*p*), 130.0 (Ar-2CH), 128.8, 127.8 (Ar-2CH-*m*), 127.5, 127.0 (Ar-2CH-*o*), 122.6 (Ar-CH-5), 124.0 (Ar-C-Br), 115.0, 110.0 (Ar-C), 40.6 (CH_2_), 37.0 ppm (CH). MS (Fab, 70 eV, %): *m/z* = 475/477(32/58), 442 (18), 442 (12), 390 (6), 369 (10), 358 (12), 340 (17), 328 (24), 153 (100). *Anal*. *Calcd. for* C_25_H_18_BrNO_4_ (475.33): C, 63.04; H, 3.81; N, 2.94. *Found*: C, 62.96; H, 3.70; N, 3.08.

#### 3.3.5. 2-(4-Hydroxy-7-Methyl-2-oxo-1,2-Dihydroquinolin-3-yl)-1,4-Diphenylbutane-1,4-Dione (**6e**)

Pale yellow crystals (DMF/EtOH), yield: 0.320 g (77%), mp 352–354 °C; IR (KBr): ν = 3230 (NH), 3010 (Ar-CH), 2890 (Aliph-CH), 1695, 1660 (CO), 1620 cm^−1^ (Ar-C=N); ^1^H-NMR (400 MHz, DMSO-*d*_6_): δ = 11.35 (s, 1H; NH), 10.77 (b, 1H; OH), 8.01 (dd, 2H, *J* = 8.5, 1.3 Hz; H-*o’*), 7.81 (d, 2H, *J* = 7.3 Hz; H-*o),* 7.73 (bs, 1H; H-5), 7.65 (t, 1H, *J* = 7.4 Hz; H-*p’*), 7.55 (“t”, 2H, *J* = 7.6 Hz; H-*m’*), 7.47 (t, 1H, *J* = 7.3 Hz; H-*p*), 7.38 (“t”, 2H, *J* = 7.6 Hz; H-*m*), 7.29 (dd, 1H, *J* = 8.4, 1.3 Hz; H-7), 7.14 (d, 1H, *J* = 8.3 Hz; H-8), 5.46 (dd, 1H, *J* = 9.7, 3.1 Hz; H-α), 4.19 (dd, 1H, *J* = 17.3, 9.8 Hz; H-α’), 2.82 (dd, 1H, *J* = 17.3, 3.1 Hz; H-α’), 2.31 ppm (s, 3H; H-6a); ^13^C-NMR (100 MHz, DMSO-*d_6_*): δ = 198.46, 198.31 (C-β,β’), 162.21 (C-2), 158.27 (C-4), 137.81, 137.01 (C-*i,i’*), 136.66 (C-8a), 132.92 (C-*p’*), 132.26 (C-*p*), 131.66 (C-7), 130.10 (C-6), 128.63 (C-*m’*), 128.19 (C-*m*), 127.83 (C-*o’*), 127.34 (C-*o*), 122.13 (C-5), 115.12 (C-8), 114.75 (C-4a), 111.68 (C-3), 40.46 (C-α), 37.45 (C-α’), 20.59 ppm (C-6a); ^15^N-NMR (40.55 MHz, DMSO-*d*_6_): δ = 142.1 ppm (N-1). MS (Fab, 70 eV, %): *m/z* = 411 = (M^+^, 40), 393 (16), 153 (100). *Anal*. *Calcd. for* C_26_H_21_NO_4_ (411.46): C, 75.90; H, 5.14; N, 3.40. *Found*: C, 76.10; H, 5.24; N, 3.50.

#### 3.3.6. 2-(4-Hydroxy-6-Methyl-2-oxo-1,2-Dihydroquinolin-3-yl)-1,4-Diphenylbutane-1,4-Dione (**6f**)

Yellow crystals (DMF/H*_2_*O), yield: 0.320 g (77%), mp = 282–284 °C, IR (KBr): ν = 3226 (NH), 3012 (Ar-CH), 2860 (Aliph-CH), 1690, 1656 (CO), 1610 cm^−1^ (Ar-C=N); ^1^H-NMR (400 MHz, DMSO-*d_6_*): δ = 12.10 (s, 1H, OH), 11.70 (s, 1H, NH), 7.98 (d, 1H, *J* = 7.7 Hz, Ar-H), 7.86 (d, 1H, *J* = 8.00 Hz, Ar-H), 7.80 (d, 1H, *J* = 7.8 Hz, Ar-H), 7.62-7.55 (m, 5H, Ar-H), 7.28 (t, 2H, *J* = 8.0 Hz, Ar-H), 7.30–7.24 (m, 3H, Ar-H), 5.48 (dd, 1H, *J* = 10.0, 2.4 Hz, CH-α), 4.22 (dd, 1H, *J* = 17.0, 9.9 Hz, CH_2_-α), 2.82 (dd, 1H, *J* = 17.0, 2.6 Hz, CH_2_-α’), 2.20 ppm (s, 3H, CH_3_); ^13^C-NMR (100 MHz, DMSO-*d_6_*): δ = 198.0, 197.6, 163.0 (CO), 158.2 (Ar-C), 136.8, 135.4, 135.0 (Ar-C), 132.4, 132.0 (Ar-CH-*p*), 131.5, 130.0 (Ar-2CH-*m*), 128.9 (Ar-2CH), 127.8, 127.6 (Ar-2CH-*o*), 123.0 (Ar-CH-5), 120.0 (Ar-C-Cl), 115.2, 110.0 (Ar-C), 40.5 (CH_2_), 37.2 (CH), 21.2 ppm (CH_3_). MS (Fab, 70 eV, %): *m/z* = 411 (M^+^, 42), 393 (10), 153 (100). *Anal*. *Calcd. for* C_26_H_21_NO_4_ (411.46): C, 75.90; H, 5.14; N, 3.40. *Found*: C, 76.00; H, 5.22; N, 3.30.

#### 3.3.7. 4-(Benzo[*d*]oxazol-2-yl)-3-Hydroxy-1*H*-Benzo[4,5]oxazolo[3,2-*a*]pyridine-1-one (**11**)

Buff crystals (DMF/CH_3_CN), yield: 0.255 g (80%), mp = 270–272 °C, IR (KBr): ν = 3030 (Ar-CH), 2860 (Aliph-CH), 1656 (CO), 1610 cm^−1^ (Ar-C=N). ^1^H-NMR (400 MHz, DMSO-*d_6_*): δ = 12.90 (s, 1H, OH), 8.40 (dd, 1H, *J* = 7.0 Hz, Ar-H), 8.00 (d, 2H, *J* = 7.3 Hz; Ar-H), 7.96 (m, 2H, *J* = 7.6 Hz; Ar-H), 7.92 (m, 1H, *J* = 8.2 Hz; Ar-H), 7.84 (m; 1H, Ar-H), 7.80 (m; 1H, Ar-H), 5.55 ppm (dd, 1H, *J* 7.5, 1.2 Hz; Ar-H). ^13^C-NMR (100 MHz, DMSO-*d_6_*): δ = 165.4 (CO), 160.0 (CH) 153.0, 149.8 (Ar-2C) 148.3, 147.0 (Ar-2C) 138.3 (Ar-C) 126.6, 126.3 (Ar-2CH) 124.41, 125.31, 124.24 (Ar-3CH) 123.7 (Ar-CH), 118.5, 116.2 (Ar-2C), 111.4 (Ar-CH), 110.2 (Ar-CH), 104.2 ppm (Ar-CH). MS (Fab, 70 eV, %): *m/z* = 318 (M^+^, 100), 200 (30), 118 (75). *Anal*. *Calcd. for* C_18_H_10_N_2_O_4_ (318.29): C, 67.93; H, 3.17; N, 8.80. *Found*: C, 68.10; H, 3.25; N, 8.90.

#### 3.3.8. (4-(Benzo[d]oxazol-2-yl)-3-Hydroxy-1-oxo-1H-Benzo[4,5]oxazolo[3,2-a]pyridin-2-yl)-1,4-Diphenylbutane-1,4-Dione (**12**)

White crystals (DMF/EtOH), yield: 0.472 g (85%), mp = 278–280 °C, IR (KBr): ν = 3010 (Ar-CH), 2895 (Aliph-CH), 1665 (CO), 1620 cm^−1^ (Ar-C=N); ^1^H-NMR (400 MHz, DMSO-*d*_6_): δ = 13.16 (b, 1H; OH), 8.45 (dd, 1H, *J* = 7.0, 2.0 Hz; H-9)., 8.03 (d, 2H, *J* = 7.3 Hz; H-*o/o’),* 7.95 (d, 2H, *J* = 7.6 Hz; H-*o’/o),* 7.90 (bd, 1H, *J* = 8.2 Hz; H-6), 7.86 (m, 1H; H-7’), 7.80 (h, 1H; H-4’), 7.66 (t, 1H, *J* = 7.3 Hz; H-*p/p’),* 7.56 (“t”, 2H, *J* = 7.6 Hz; H-*m/m’),* 7.54 (m, 1H; H-7), 7.52 (m, 1H; H-8), 7.49 (t, 1H, *J* = 7.3 Hz; H-*p’/p),* 7.43 (m, 2H; H-5’,6’), 7.41 (“t”, 2H, *J* = 7.3 Hz; H-*m’/m),* 5.55 (dd, 1H, *J* = 10.1, 2.9 Hz; H-2a), 4.36 (dd, 1H, *J* = 17.4, 10.2 Hz; H-2b), 2.85 ppm (dd, 1H, *J* = 17.2, 2.6 Hz; H-2b); ^13^C-NMR (100 MHz, DMSO-*d*_6_): δ = 198.58, 197.93(C-2b’, 2c), 159.67 (C-3), 156.92 (C-1), 152.93 (C-4a), 149.58 (C-2’), 148.62 (C-7a’), 146.99 (C-5a), 138.39 (C-3a’), 136.98, 136.37 (C-*i, i’),* 132.94, 132.49 (C-*p, p’),* 128.66, 128.41 (C-*m, m’),* 127.85, 127.55 (C-*o, o’),* 126.86, 126.53 (C-7, 9a), 125.41, 125.31, 125.24 (C-5’, 6’, 8), 123.86 (C-2), 118.38 (C-4’), 115.92 (C-9), 111.24 (C-6), 110.83 (C-7’), 103.22 (C-4), 39.5 (C-2a), 37.35 ppm (C-2b). MS (Fab, 70 eV, %): *m/z* = 554 (M^+^, 40), 449 (25), 154 (100). *Anal*. *Calcd. for* C_34_H_22_N_2_O_6_ (554.15): C, 73.64; H, 4.00; N, 5.05. *Found*: C, 73.69; H, 4.05; N, 5.11.

### 3.4. Crystal Structure Determinations

The single-crystal X-ray diffraction study were carried out on a Bruker D8 Venture diffractometer (company, city, state abbr. if USA, country) with Photon100 detector at 123(2) K using Cu-K_α_ radiation (λ = 1.54178 Å. Direct methods (SHELXS-97 for **6f**) [30] or dual space/intrinsic methods (SHELXT for **12**) [31] were used for structure solution and refinement was carried out using SHELXL-2014 (full-matrix least-squares on *F^2^*; version, company, city, state abbr. if USA, country) [31]. Hydrogen atoms were localized by difference electron density determination and refined using a riding model (H(N,O)free). Semi-empirical absorption corrections were applied.

**6f**: colourless crystals, C_26_H_21_NO_4_, *M*_r_ = 411.44, crystal size 0.16 × 0.08 × 0.06 mm, triclinic, space group *P-1* (No. 2), *a* = 11.1823(3) Å, *b* = 14.3827(4) Å, *c* = 15.3460(4) Å, α = 67.695(1)°, β = 70.666(1)°, γ = 68.389(1)°, *V* = 2069.77(10) Å^3^, *Z* = 4, ρ = 1.320 Mg/m^−3^, µ(Cu-K_α_) = 0.72 mm^−1^, *F*(000) = 864, *2*θ_max_ = 144.2°, 28,579 reflections, of which 8117 were independent (*R*_int_ = 0.035), 577 parameters, 5 restraints, *R*_1_ = 0.043 (for 6749 I > 2σ(I)), w*R*_2_ = 0.112 (all data), *S* = 1.01, largest diff. peak/hole = 0.37/−0.50 e Å^−3^.

**12**: colourless crystals, C_34_H_2_N_2_O_6_, *M*_r_ = 554.53, crystal size 0.22 × 0.09 × 0.03 mm, triclinic, space group *P-1* (No. 2), *a* = 8.2672(2) Å, *b* = 11.3140(3) Å, *c* = 13.7326(3) Å, α = 91.721(1)°, β = 96.833(1)°, γ = 92.198(1)°, *V* = 1273.64(5) Å^3^, *Z* = 2, ρ = 1.446 Mg/m^−3^, µ(Cu-K_α_) = 0.82 mm^−1^, *F*(000) = 576, *2*θ_max_ = 144.4°, 18,981 reflections, of which 4987 were independent (*R*_int_ = 0.037), 382 parameters, 1 restraint, *R*_1_ = 0.048 (for 4212 I > 2σ(I)), w*R*_2_ = 0.127 (all data), *S* = 1.03, largest diff. peak/hole = 0.35/−0.25 e Å^−3^.CCDC 1,913,026 (**6f**), and 1,913,027 (**12**) contain the supplementary crystallographic data for this paper. These data can be obtained free of charge from The Cambridge Crystallographic Data Centre via www.ccdc.cam.ac.uk/data_request/cif.

## 4. Conclusions

Reaction of 1,6-disubstituted-4-hydroxy-quinolin-2-ones with 2,3-dichloropyrazine*,* catalyzed by triethylamine, furnished5:12-dihydropyrazino-[2,3-*c*:5,6-*c*′]difuro[2,3-*c*:4,5-*c*′]diquinoline-6,14(*5*H,12*H*)-diones, by an apparent S_N_Ar/ANRORC sequence. Reaction of the same quinolones with *(E)-*dibenzoylethene led to conjugate addition without cyclization. Reaction of 2-hydroxyaniline with diethyl malonate led unexpectedly to 4-(benzo-[*d*]oxazol-2-yl)-3-hydroxy-1*H*-[4,5]oxazolo-[3,2-*a*]pyridine-1-one, which also underwent conjugate addition to dibenzoylethene.

## Supplementary Materials

The following are available online at https://www.mdpi.com/1420-3049/24/20/3782/s1, Table S1. NMR spectroscopic data of compound **6f** and Table S2. NMR spectroscopic data of compound **12.**
