# Supplementary material for: Synthesis of New Fused Heterocyclic 2-Quinolones and 3-Alkanonyl-4-Hydroxy-2-Quinolones
Source: Molecules. 2019 Oct 21;24(20):3782. doi: 10.3390/molecules24203782 (PMC6832483; doi:10.3390/molecules24203782)
Supplement: Supplementary file 1 [file molecules-24-03782-s001.zip › 06-10-2019-revised Suppl. Data.docx]

Synthesis of new fused heterocyclic 2-quinolones and 3-alkanonyl-4-hydroxy-2-quinolones

**Ashraf A. Aly,^1^* Alaa A. Hassan,^1^ Nasr K. Mohamed,^1^ Stefan Bräse,^2,3^ Lamiaa E. Abd El-Haleem,^1^ Mika Polamo,^3^ Martin Nieger^3^ and Alan B. Brown^4^**


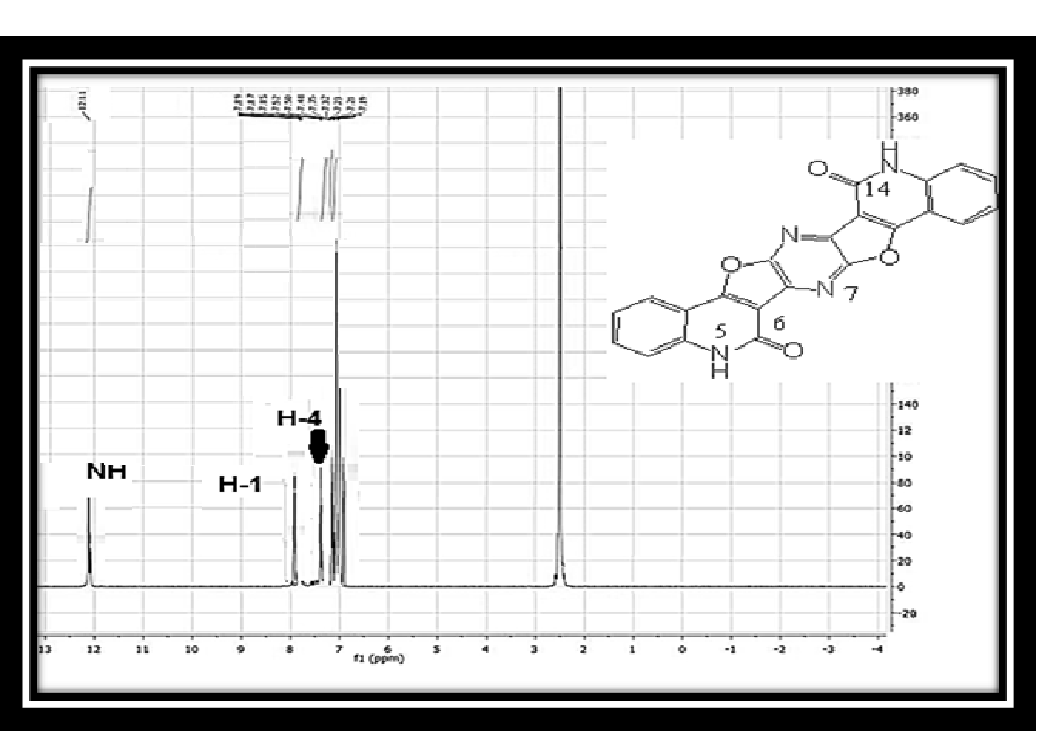


**Figure 1. ^1^**H NMR spectrum of compound **3a**

**
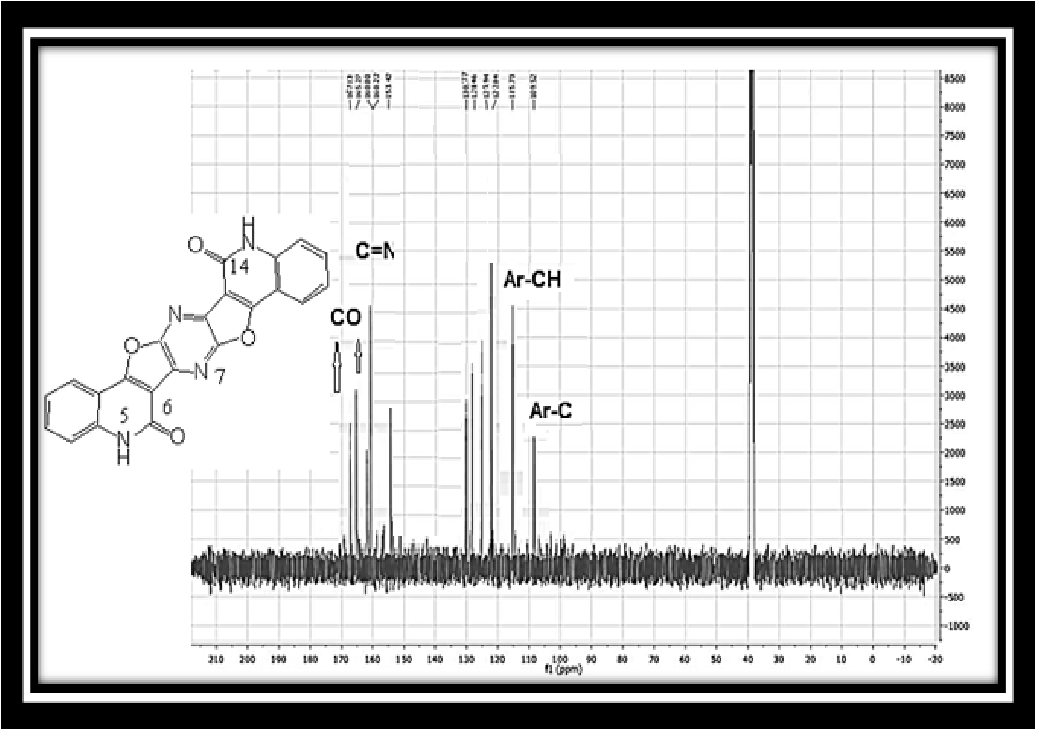
**

**Figure 2. ^13^C** NMR spectrum of compound **3a**

**
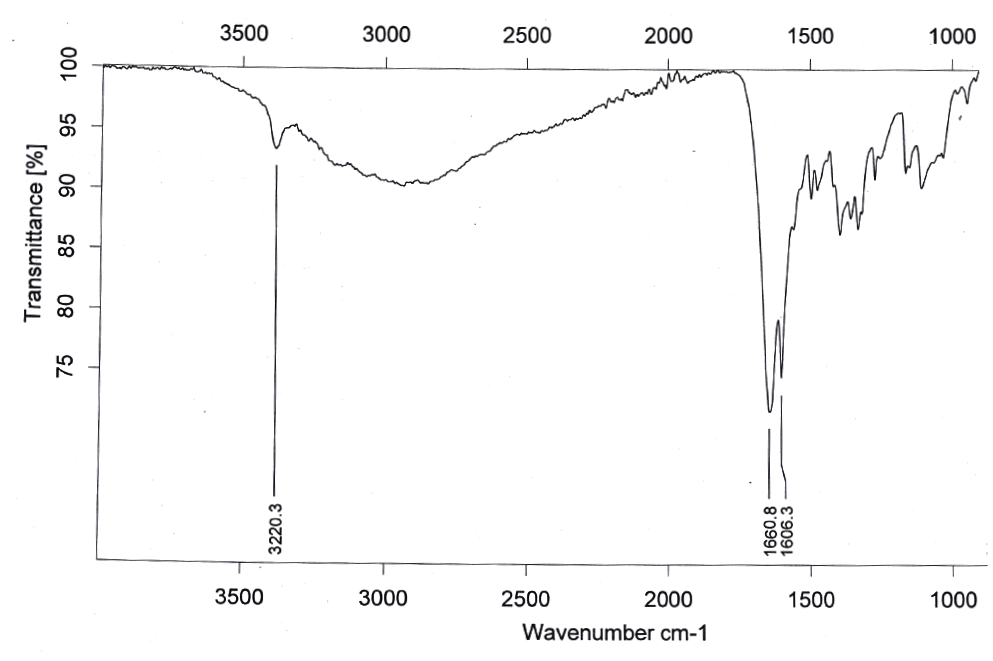
**

**Figure 3.** IR spectrum of compound **3a**

**
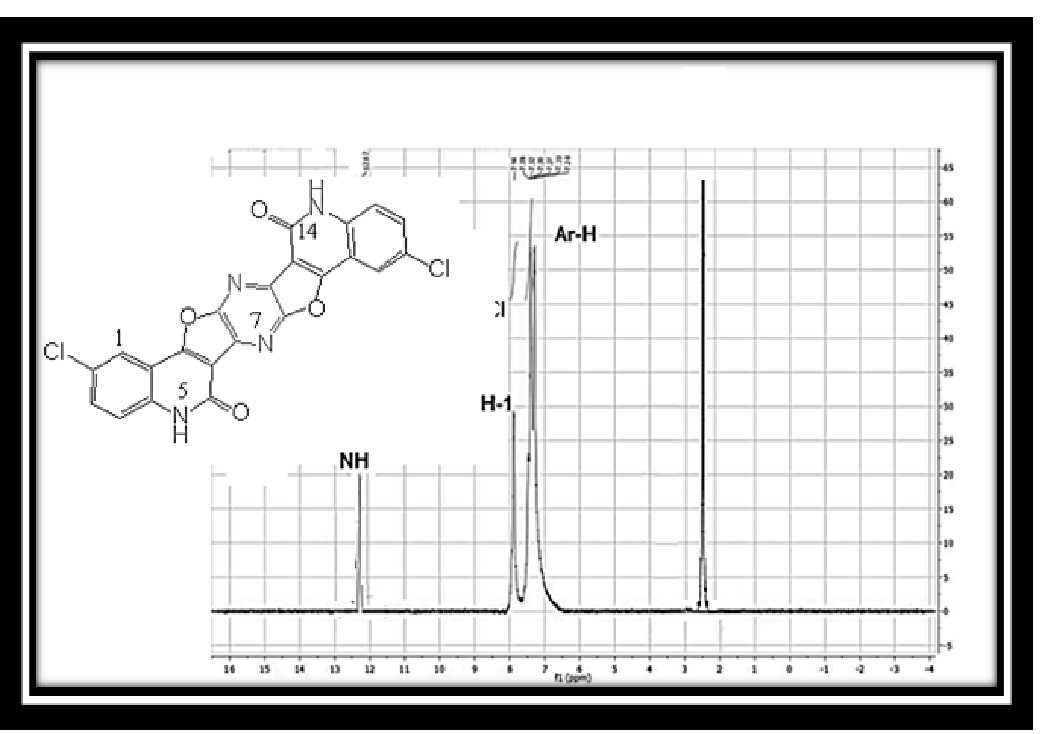
**

**Figure 4. ^1^**H NMR spectrum of compound **3b**

**
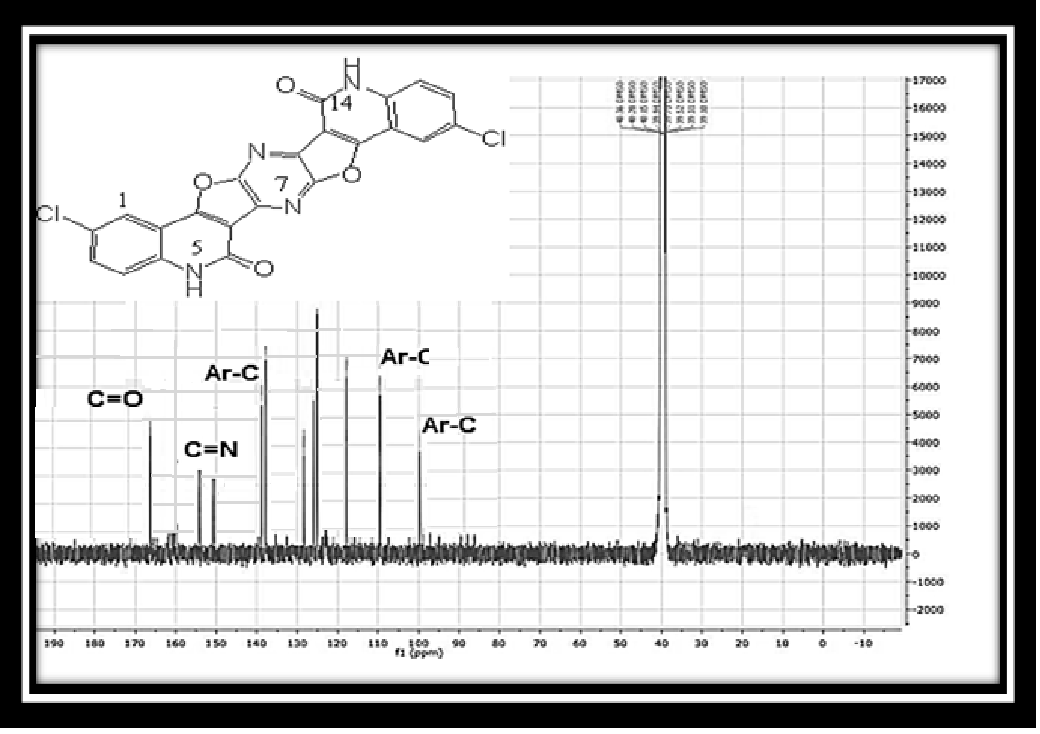
**

**Figure 5. ^13^C** NMR spectrum of compound **3b**

**
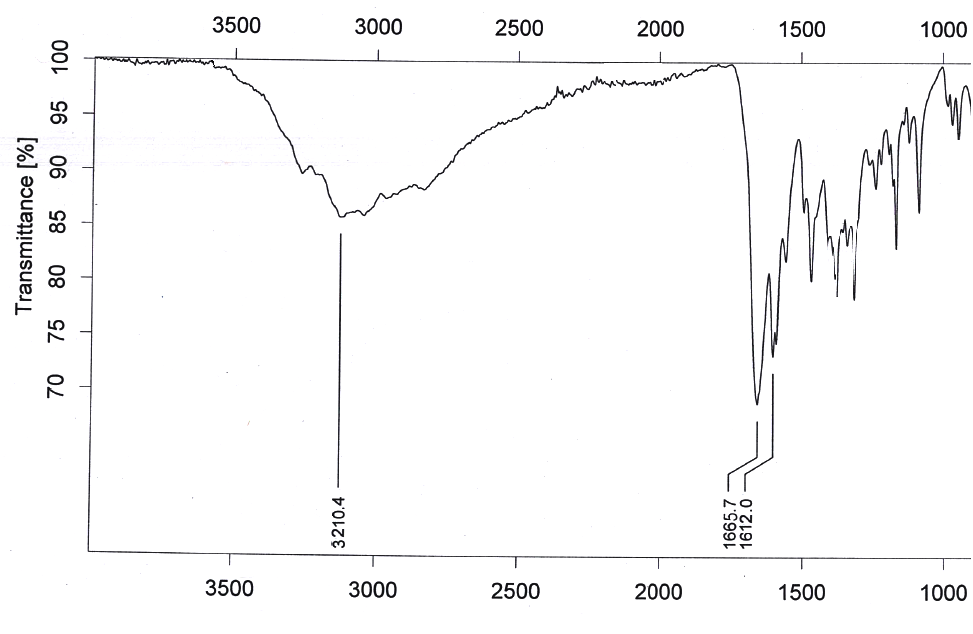
**

**Figure 6.** IR spectrum of compound **3b**

**
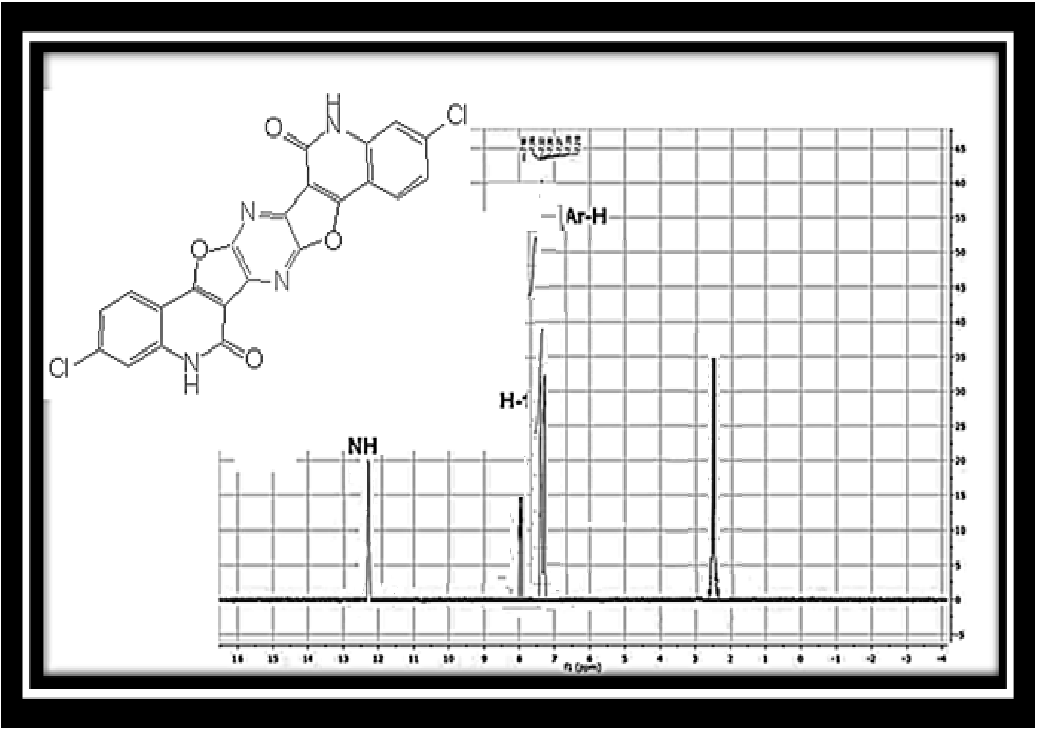
**

**Figure 7. ^1^**H NMR spectrum of compound **3c**

**
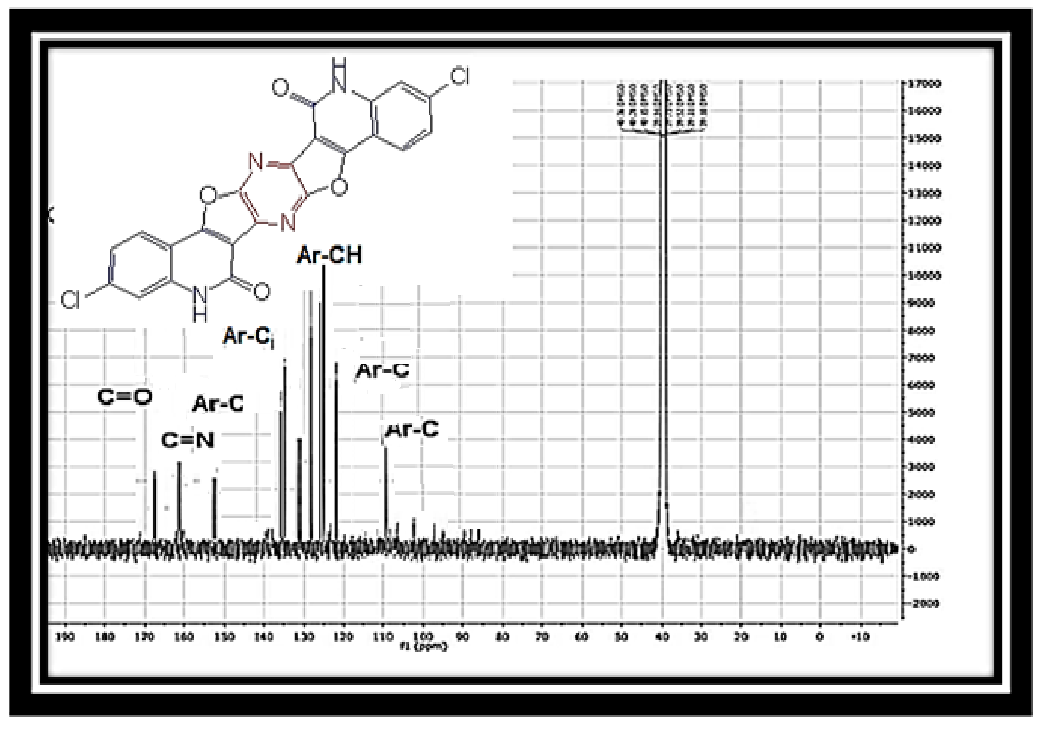
**

**Figure 8. ^13^C** NMR spectrum of compound **3c**

**
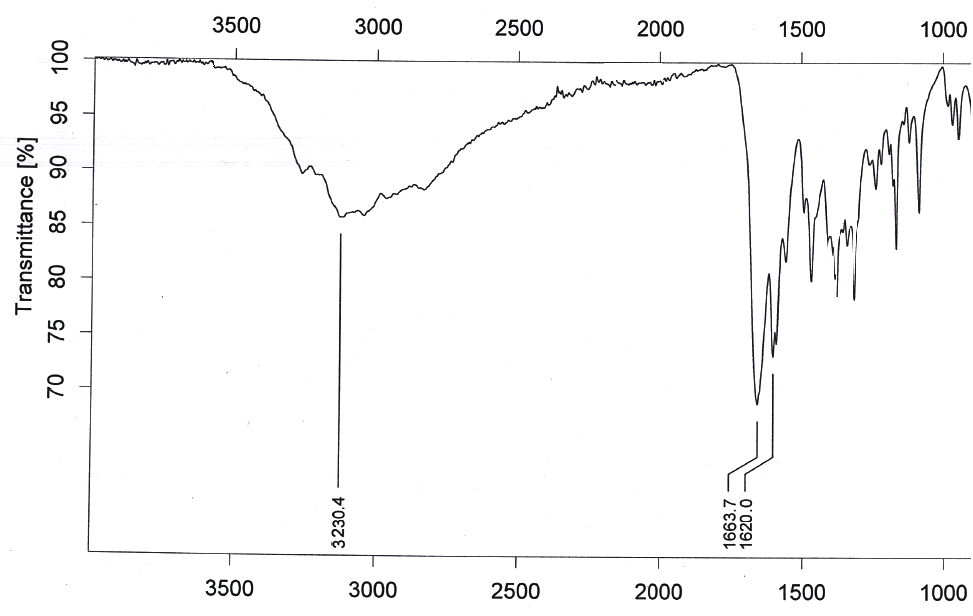
**

**Figure 9.** IR spectrum of compound **3c**

**
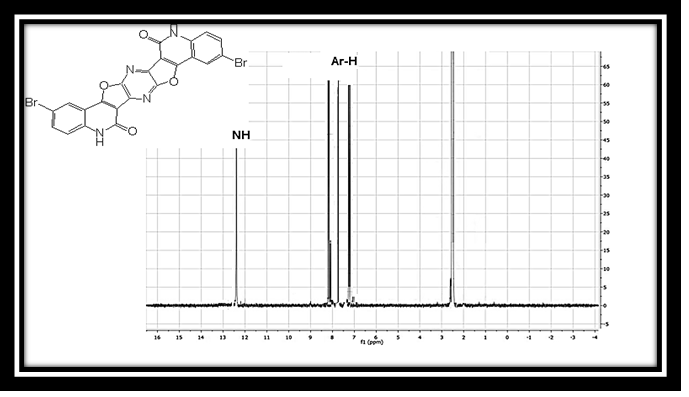
**

**Figure 10. ^1^**H NMR spectrum of compound **3d**

**
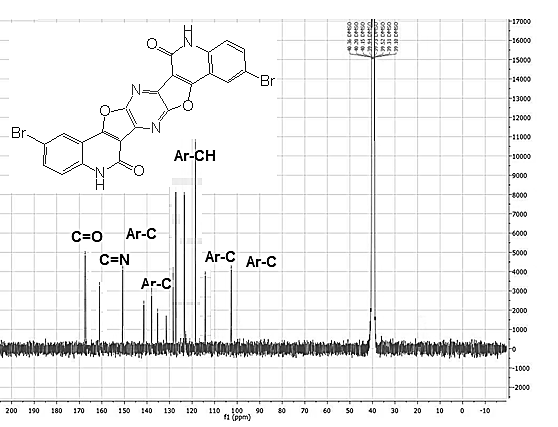
**

**Figure 11. ^13^C** NMR spectrum of compound **3d**

**
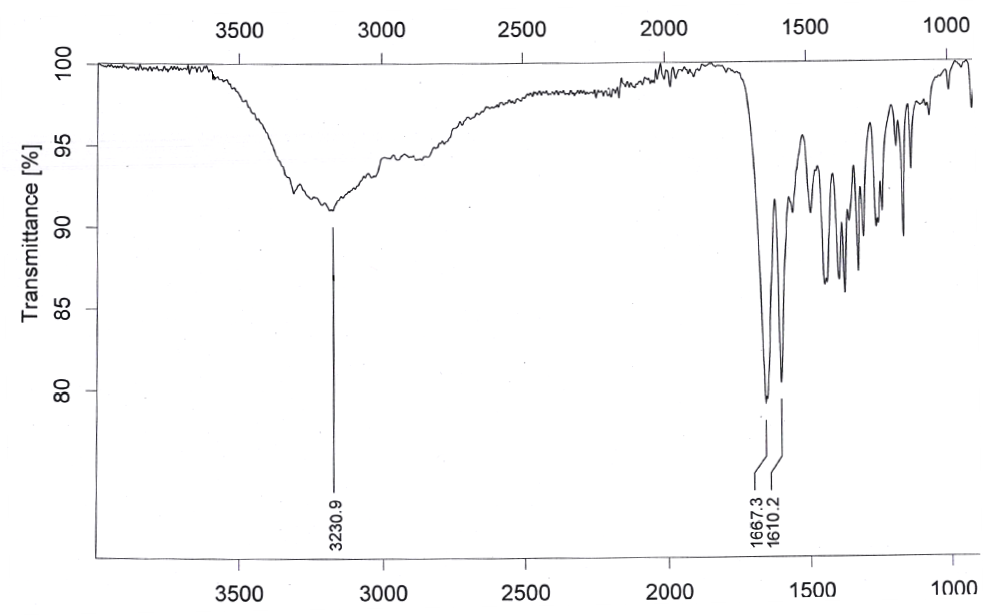
**

**Figure 12.** IR spectrum of compound **3d**

**
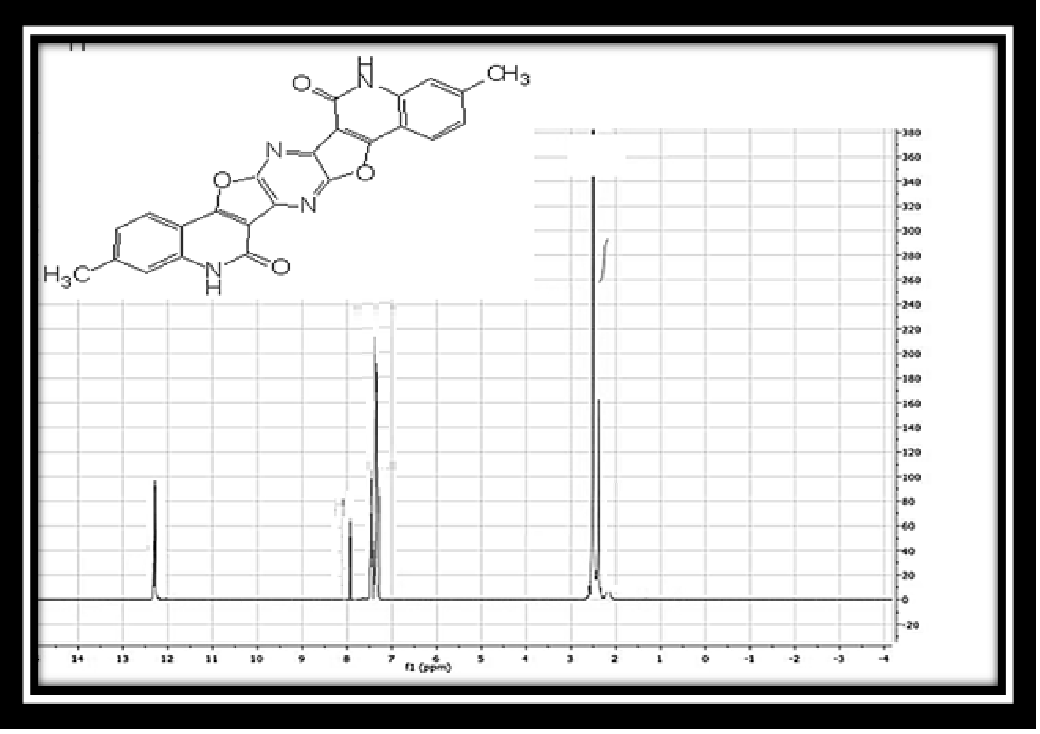
**

**Figure 13. ^1^**H NMR spectrum of compound **3e**

**
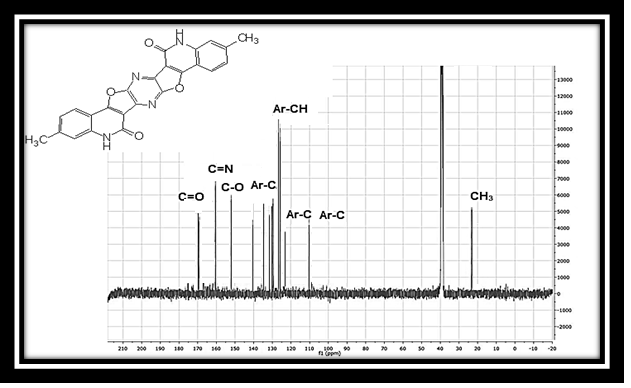
**

**Figure 14. ^13^C** NMR spectrum of compound **3e**

**
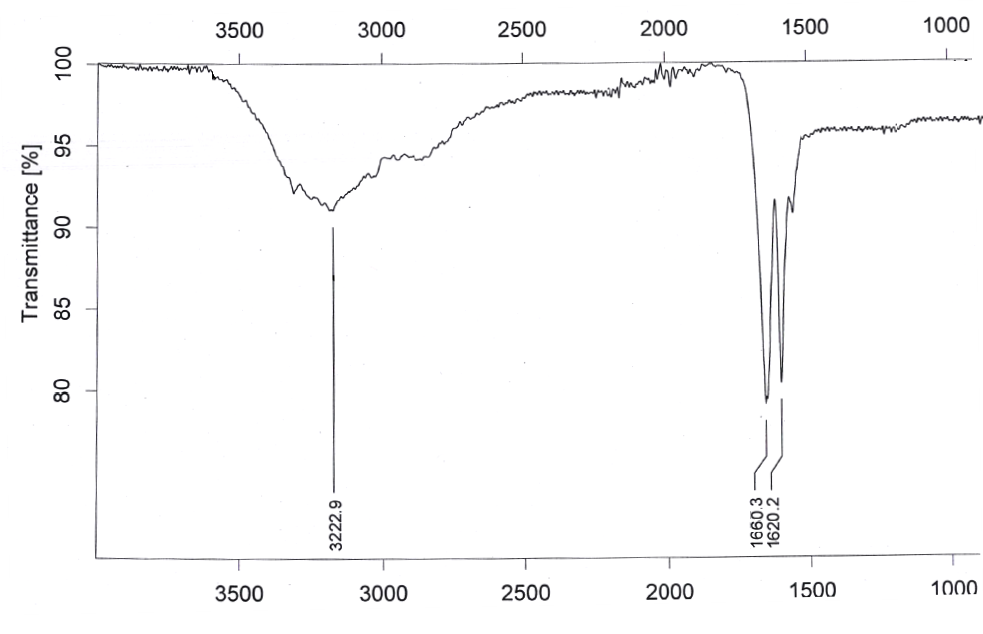
**

**Figure 15.** IR spectrum of compound **3e**

**
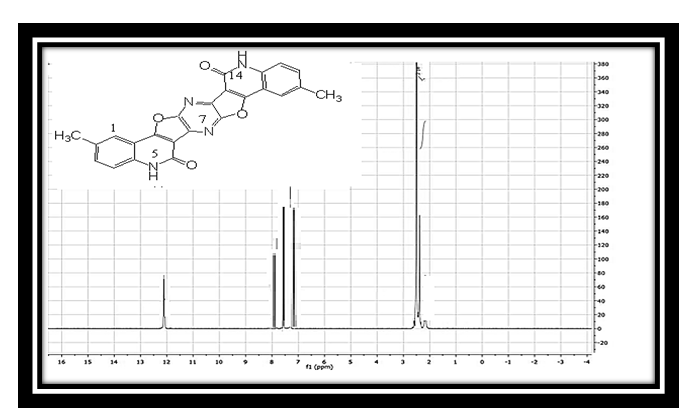
**

**Figure 16. ^1^**H NMR spectrum of compound **3f**

**
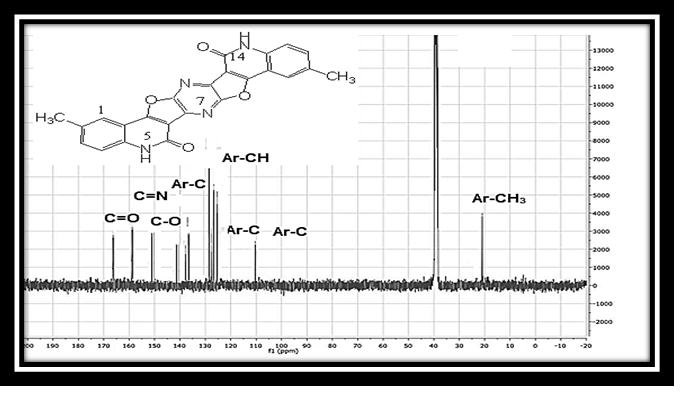
**

**Figure 17. ^13^C** NMR spectrum of compound **3f**

**
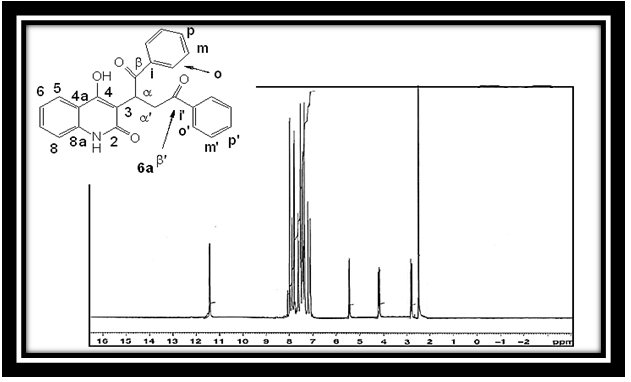
**

**Figure 18. ^1^**H NMR spectrum of compound **6a**

**Figure 19. ^1^**H NMR spectrum of compound **6a** (from δ = 7 to 8.2)

**Figure 20. ^1^**H NMR spectrum of compound **6a** (from δ = 2.5 to 5.3)

**
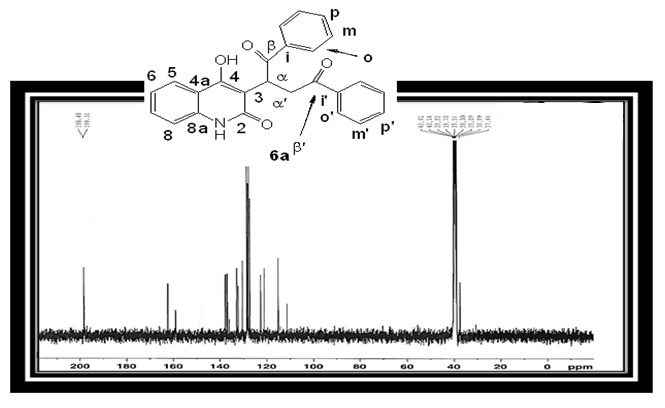
**

**Figure 21. ^13^C** NMR spectrum of compound **6a**

**
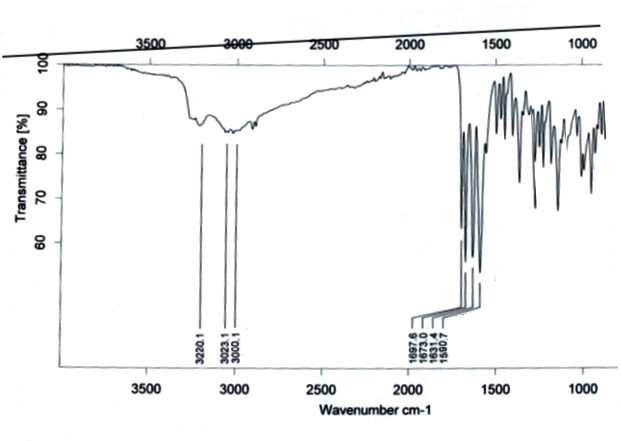
**

**Figure 22.** IR spectrum of compound **6a**

**
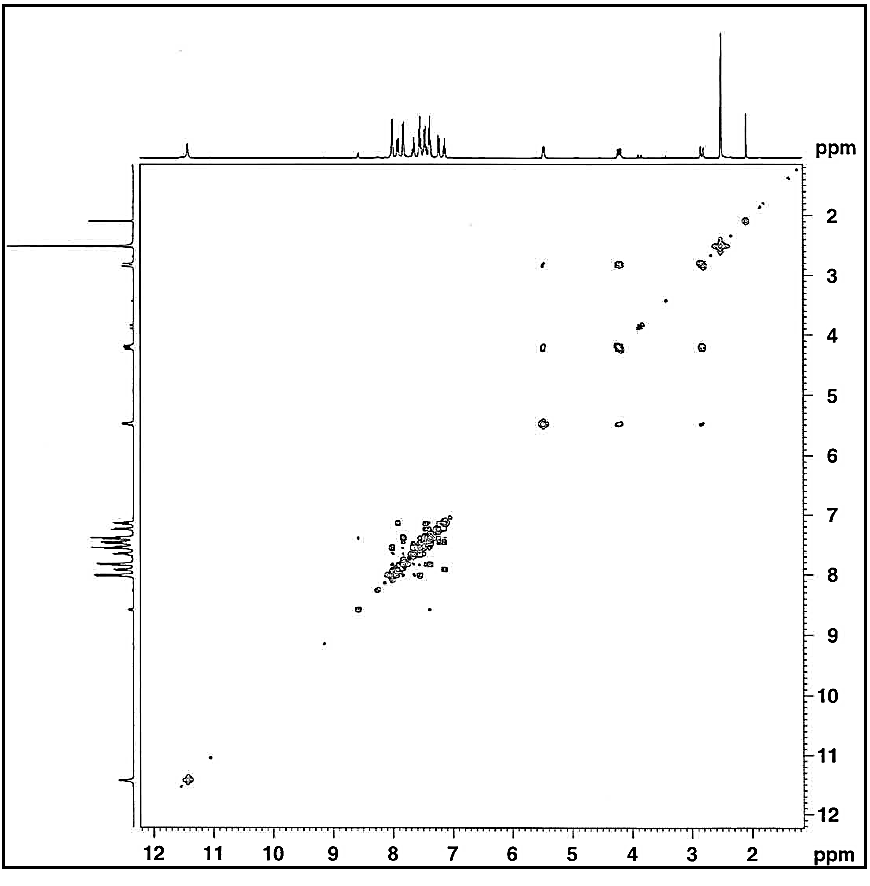
**

**Figure 23. ^1^H-^1^H** COSY NMR spectrum of compound **6a**

**
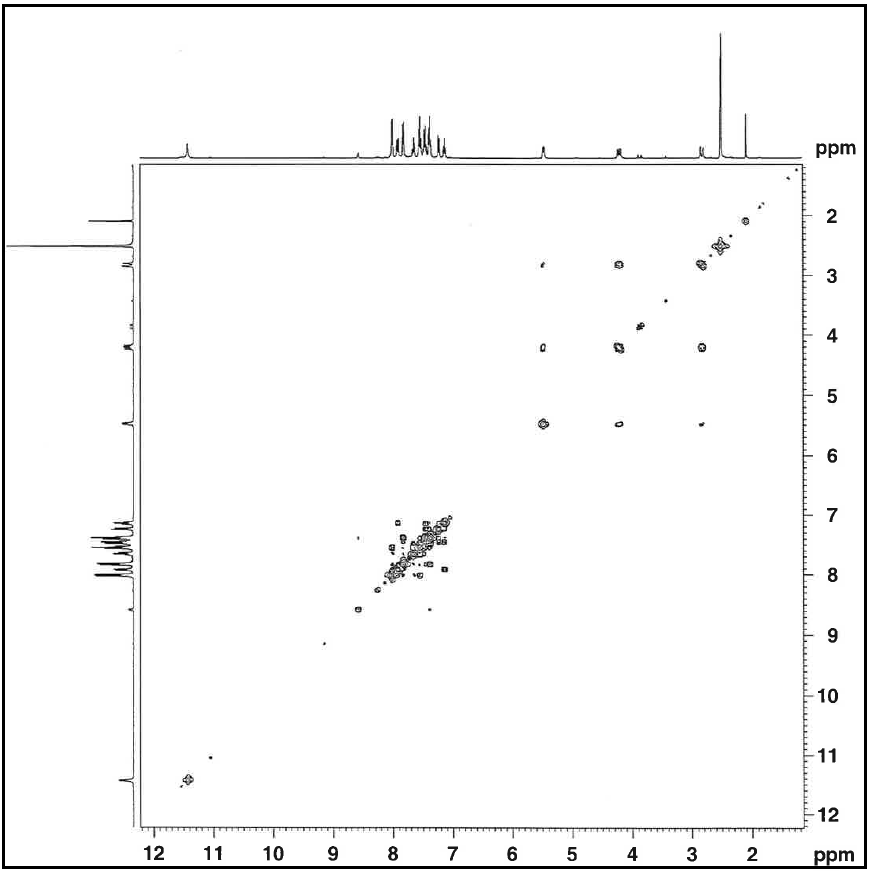
**

**Figure 24. ^1^H-^13^C** HSQC NMR spectrum of compound **6a**

**
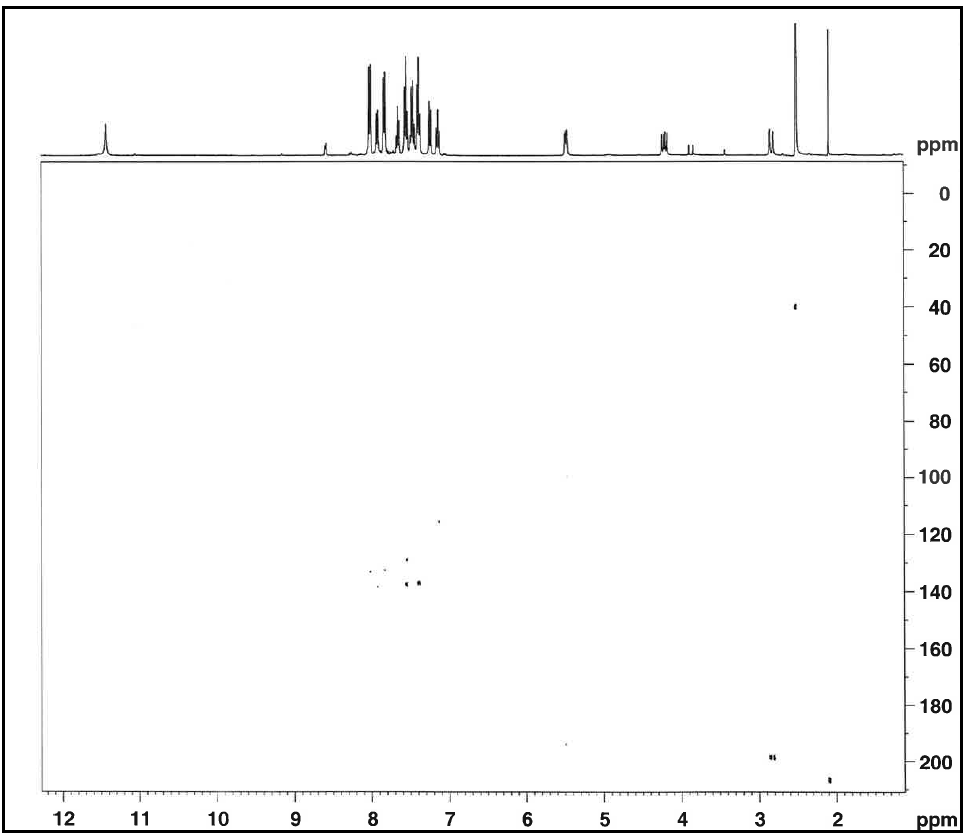
**

**Figure 25. ^1^H-^13^C** HMBC NMR spectrum of compound **6a**

**
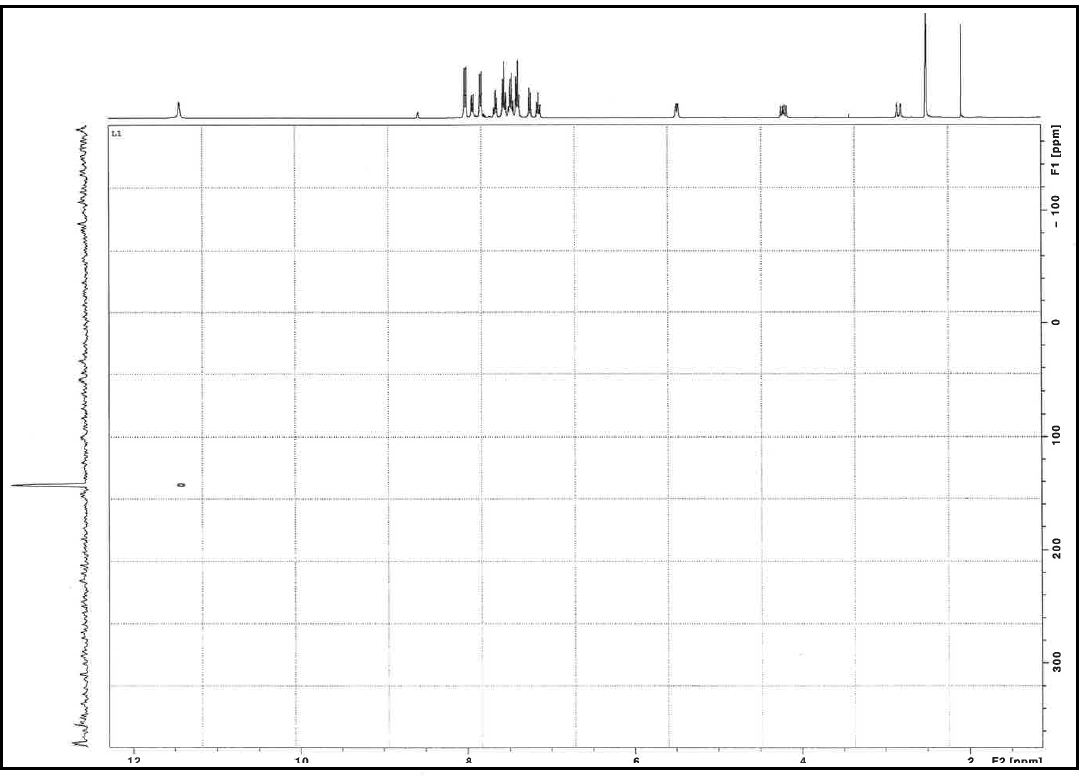
**

**Figure 26. ^1^H-^15^N** HSQC NMR spectrum of compound **6a**

**
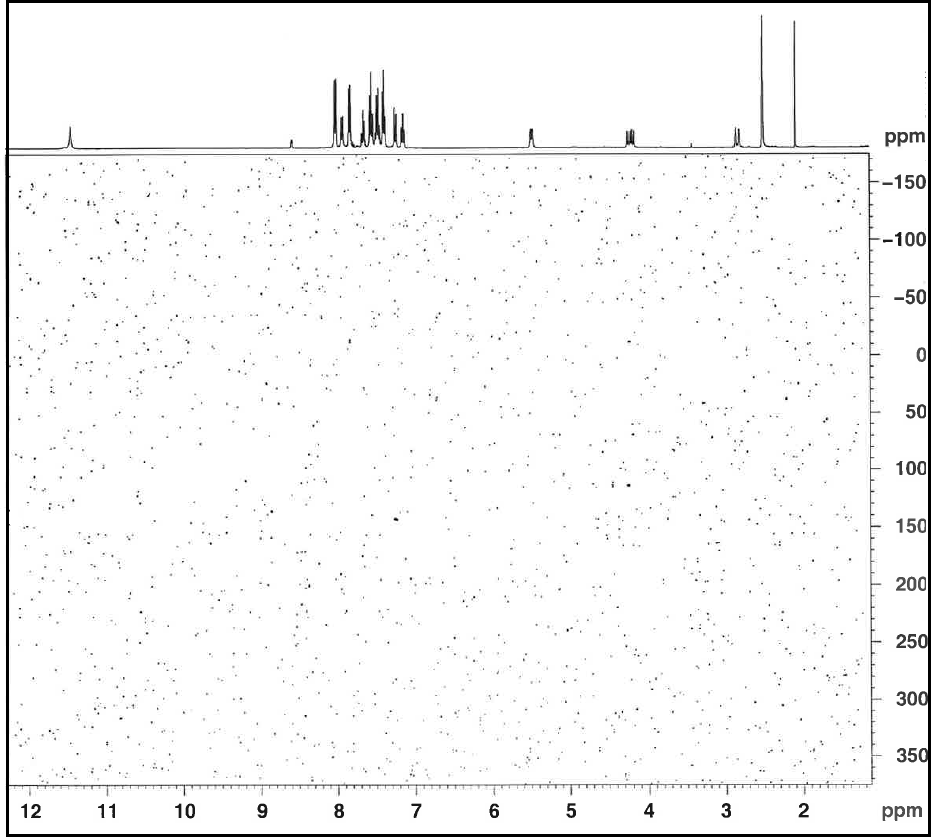
**

**Figure 27. ^1^H-^15^N** HMBC NMR spectrum of compound **6a**

**
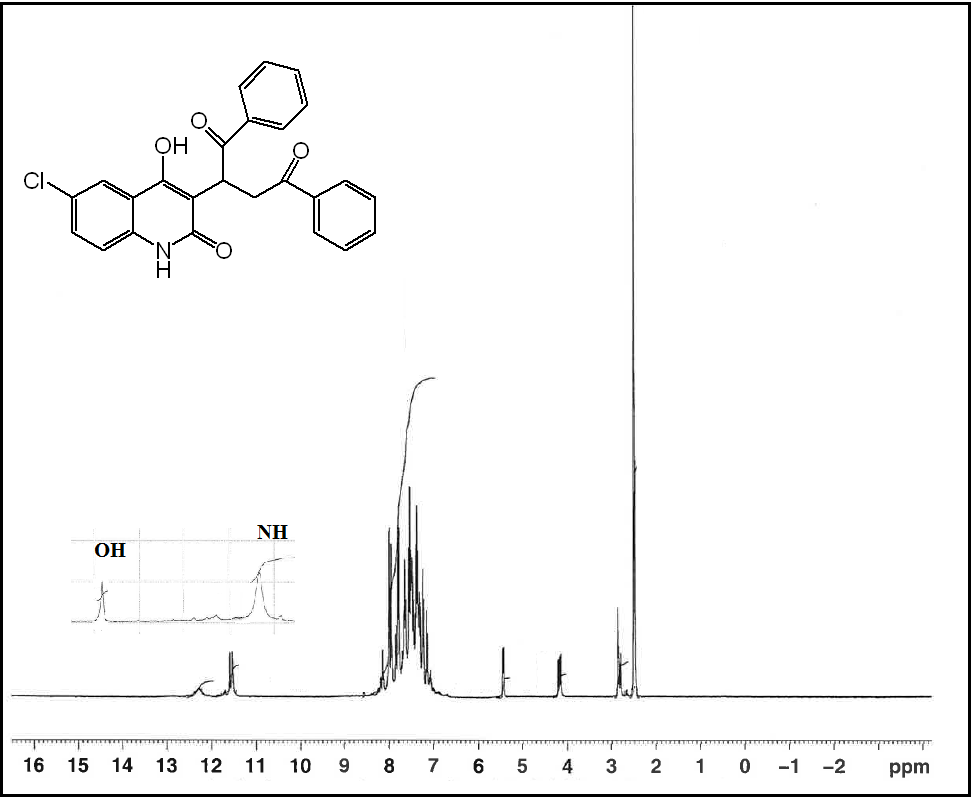
**

**Figure 28. ^1^H** NMR spectrum of compound **6b**

**
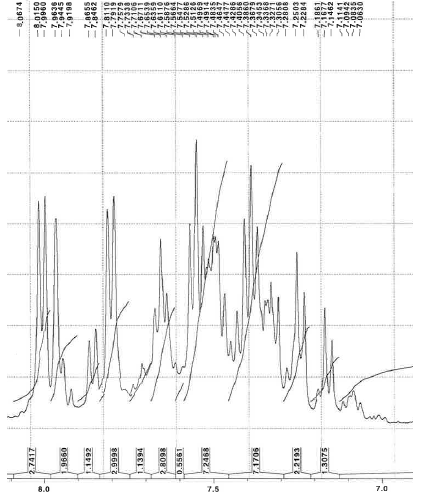
**

**Figure 29.^1^**H NMR spectrum of compound **6b** (from δ = 7 to 8.2)

**
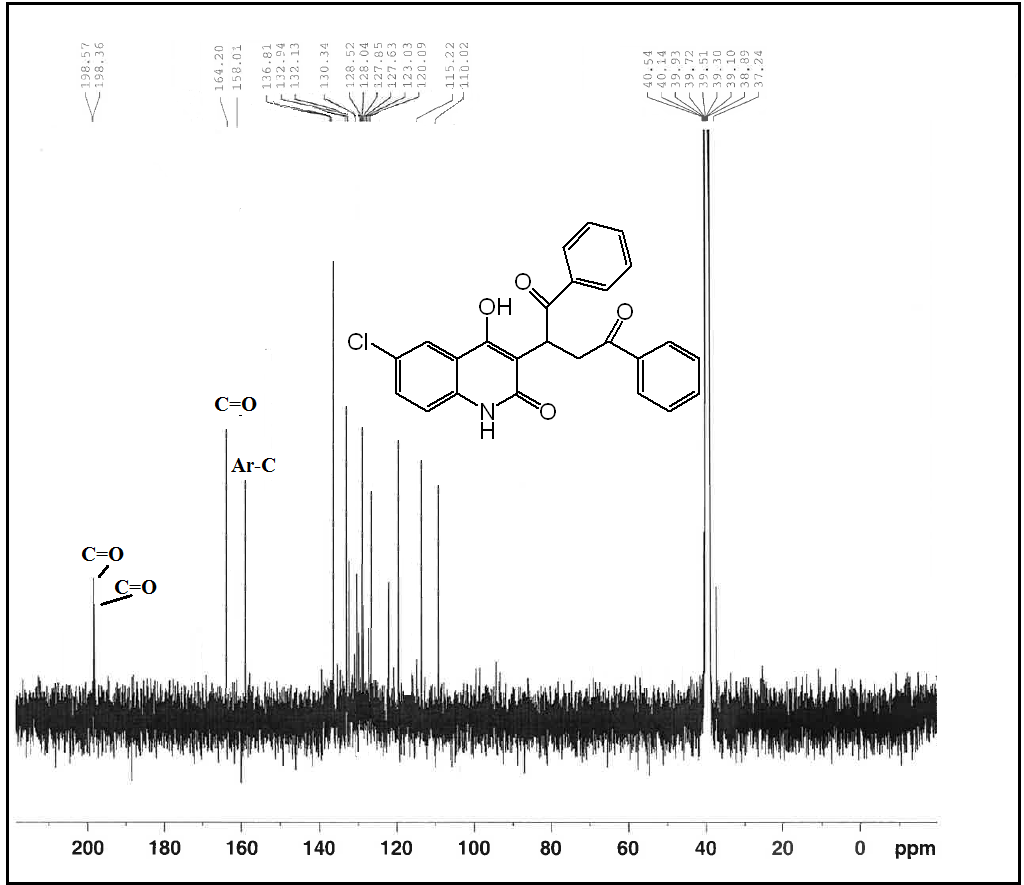
**

**Figure 30. ^13^C** NMR spectrum of compound **6b**

**
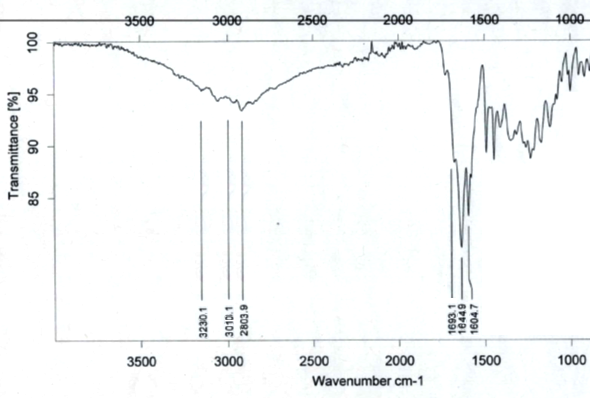
**

**Figure 31.** IR spectrum of compound **6b**

**
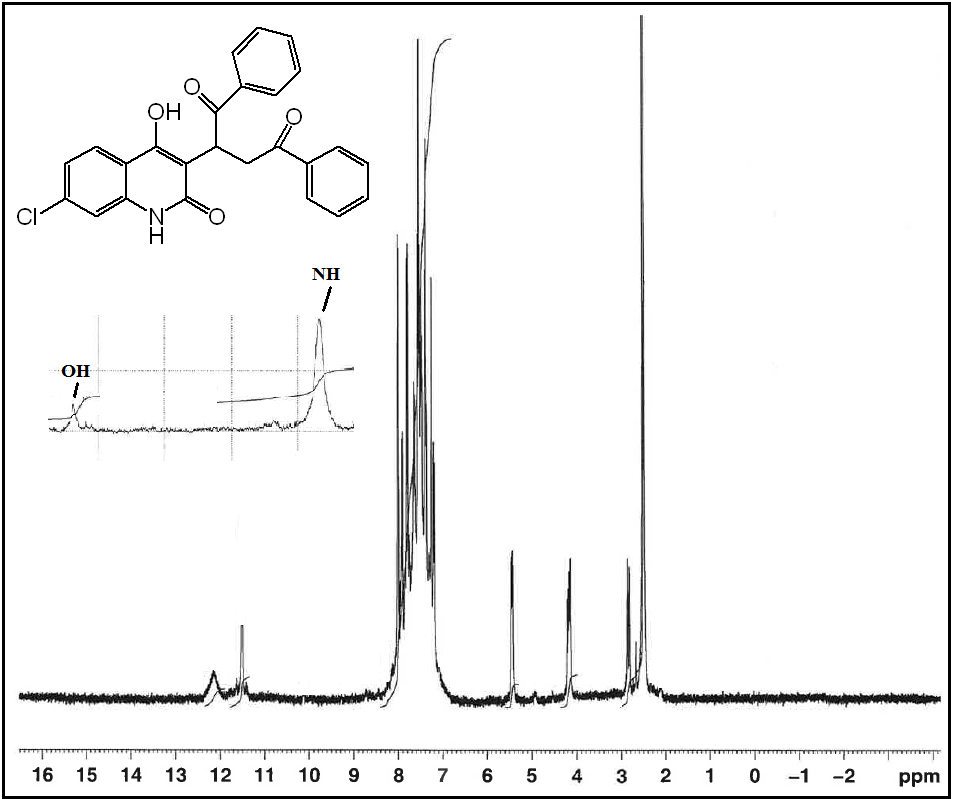
**

**Figure 32. ^1^H** NMR spectrum of compound **6c**

**
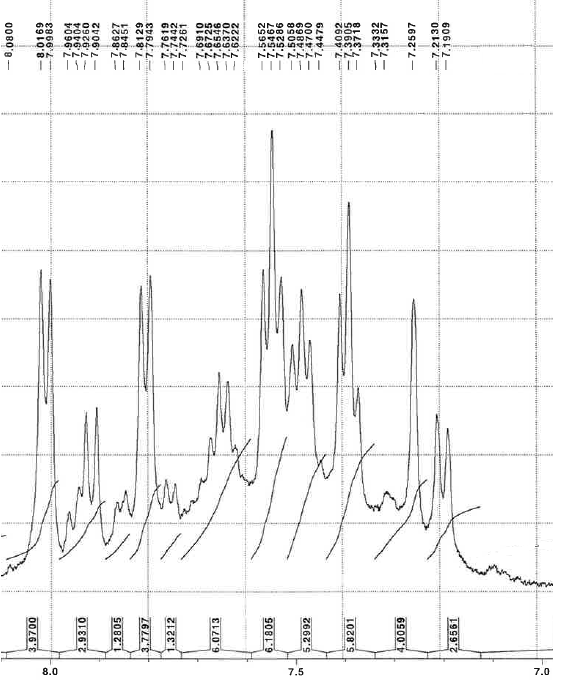
**

**Figure 33.^1^**H NMR spectrum of compound **6c** (from δ = 7 to 8.2)

**
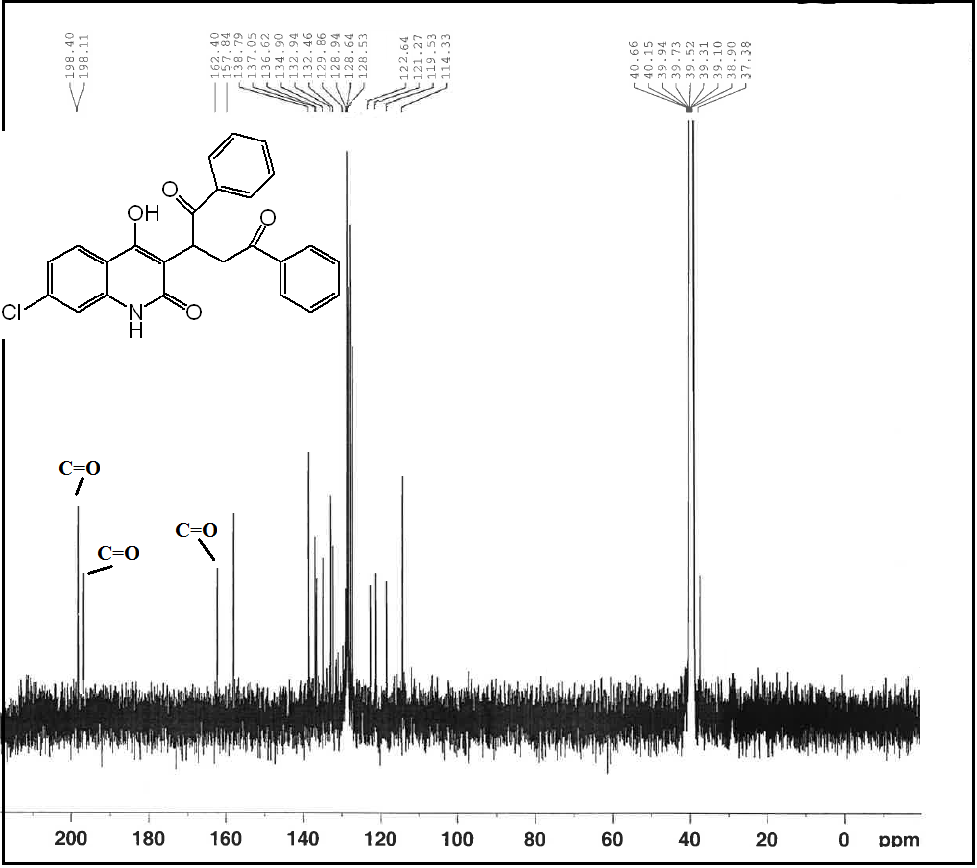
**

**Figure 34. ^13^C** NMR spectrum of compound **6c**.


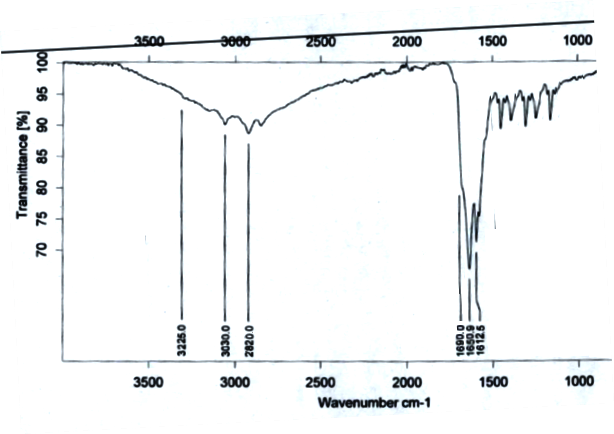


**Figure 35.** IR spectrum of compound **6c**.

**
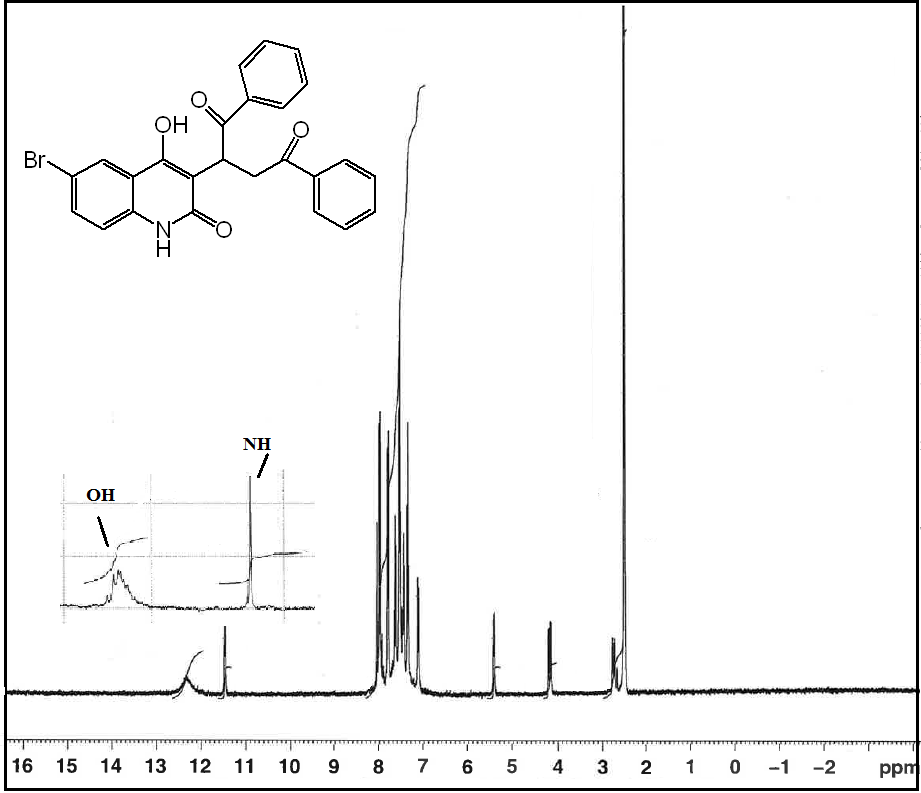
**

**Figure 36. ^1^H** NMR spectrum of compound **6d**.

**
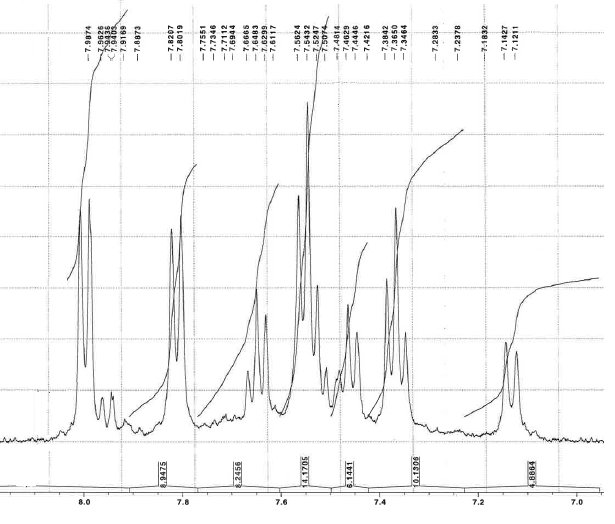
**

**Figure 37.^1^**H NMR spectrum of compound **6d** (from δ = 7 to 8.2)

**
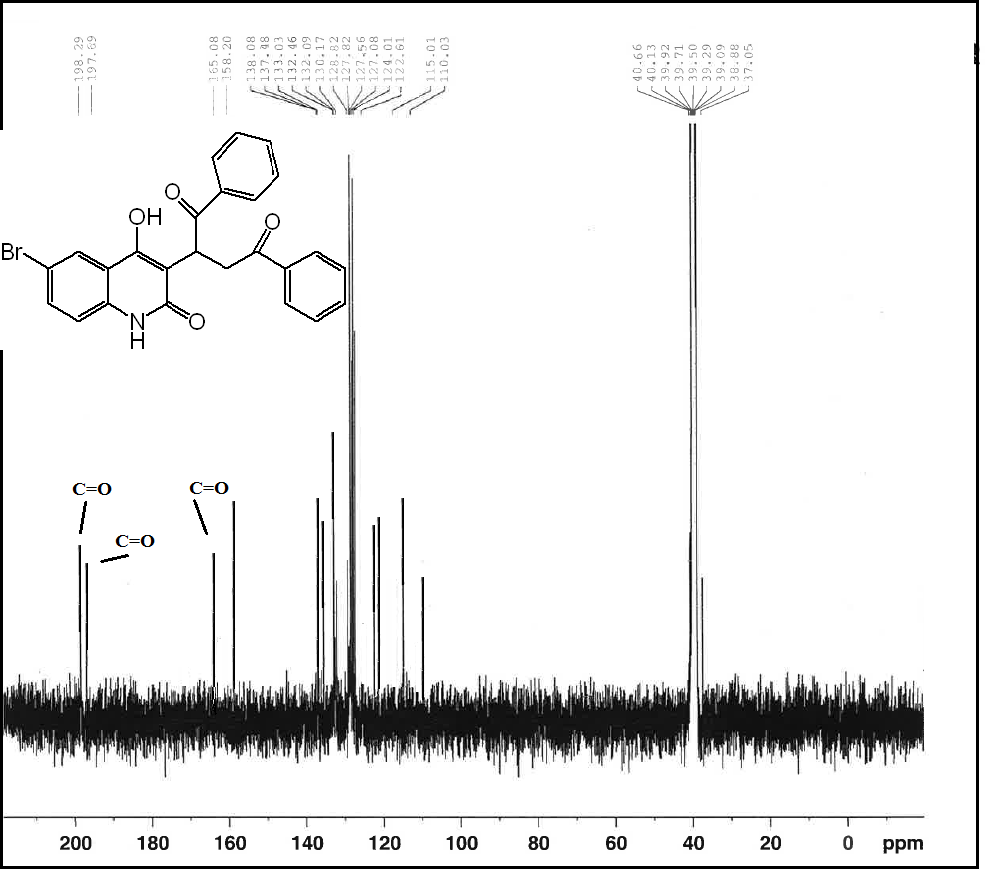
**

**Figure 38. ^13^C** NMR spectrum of compound **6d**.


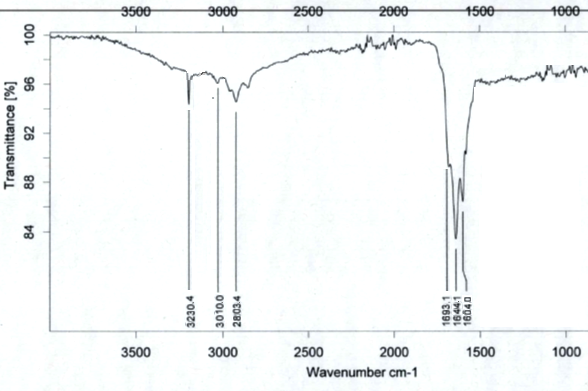


**Figure 39.** IR spectrum of compound **6d**.

**
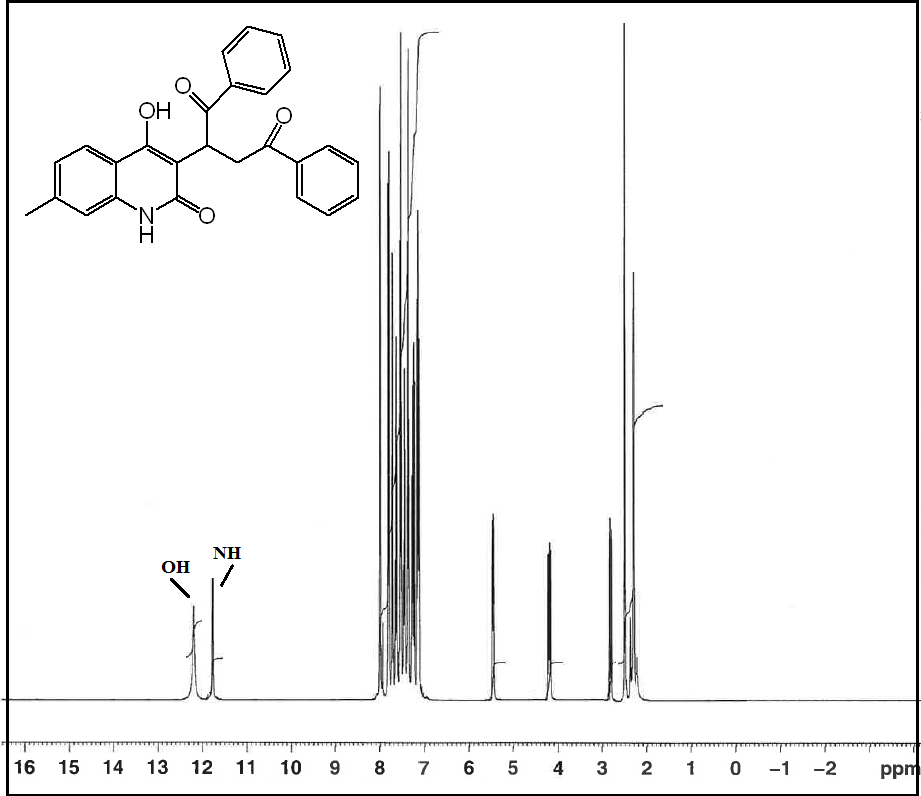
**

**Figure 40. ^1^H** NMR spectrum of compound **6e**.

**
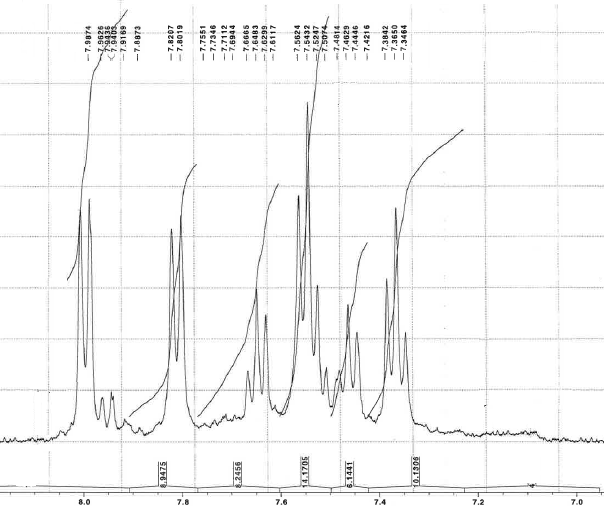
**

**Figure 41.^1^**H NMR spectrum of compound **6e** (from δ = 7 to 8.2).

**
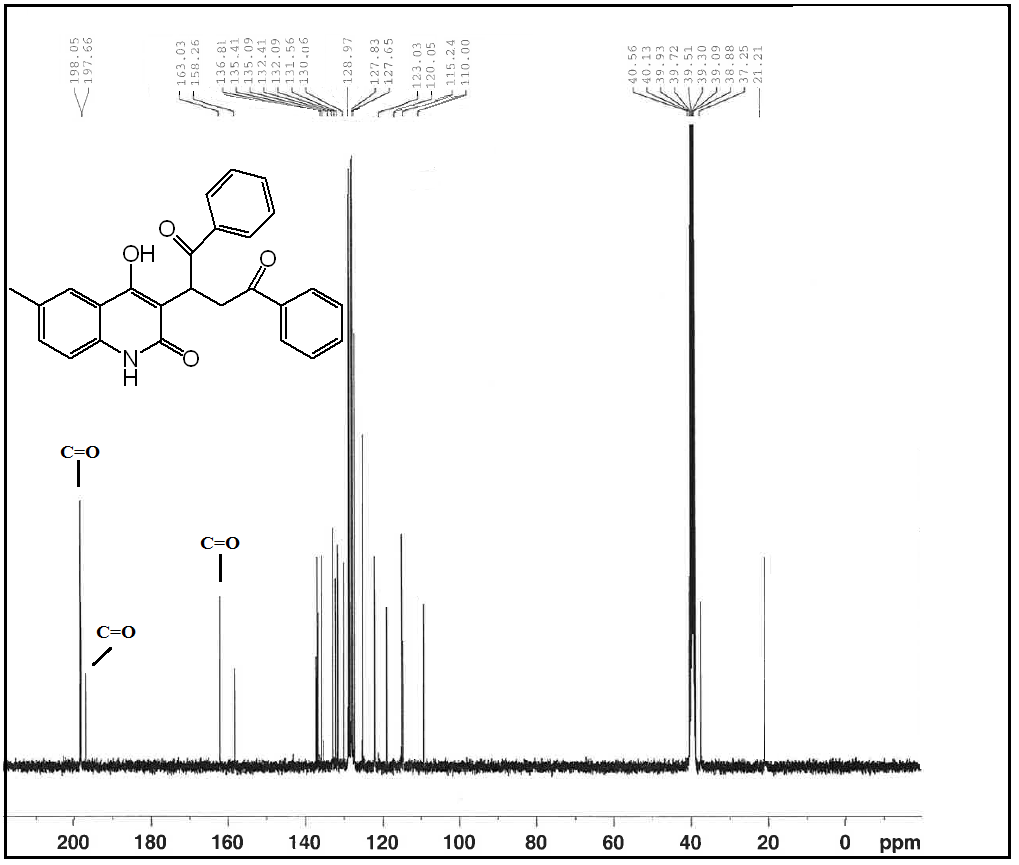
**

**Figure 42. ^13^C** NMR spectrum of compound **6e**.


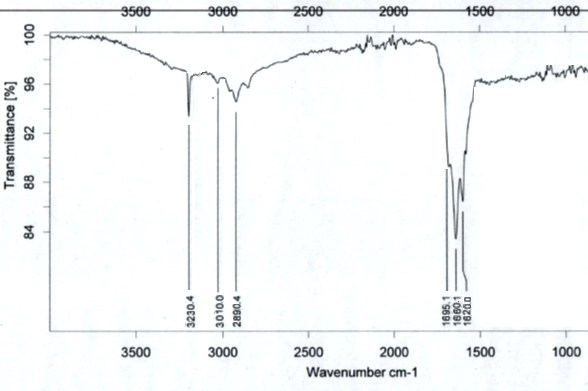


**Figure 43.** IR spectrum of compound **6e**.

**
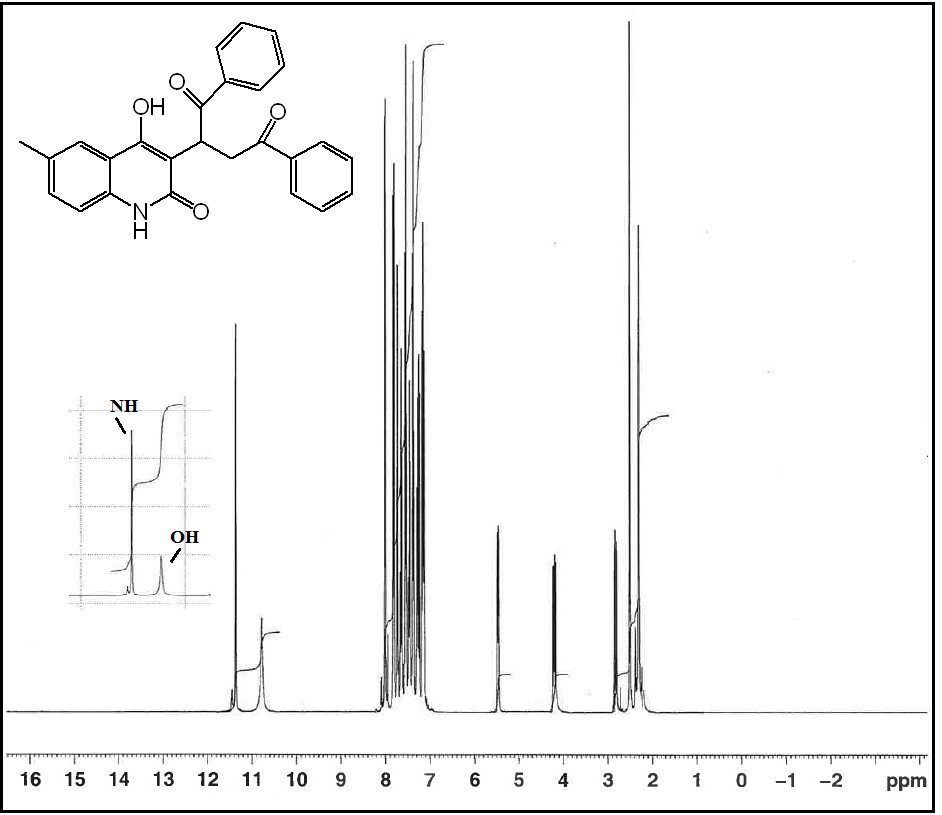
**

**Figure 44. ^1^H** NMR spectrum of compound **6f**.

**
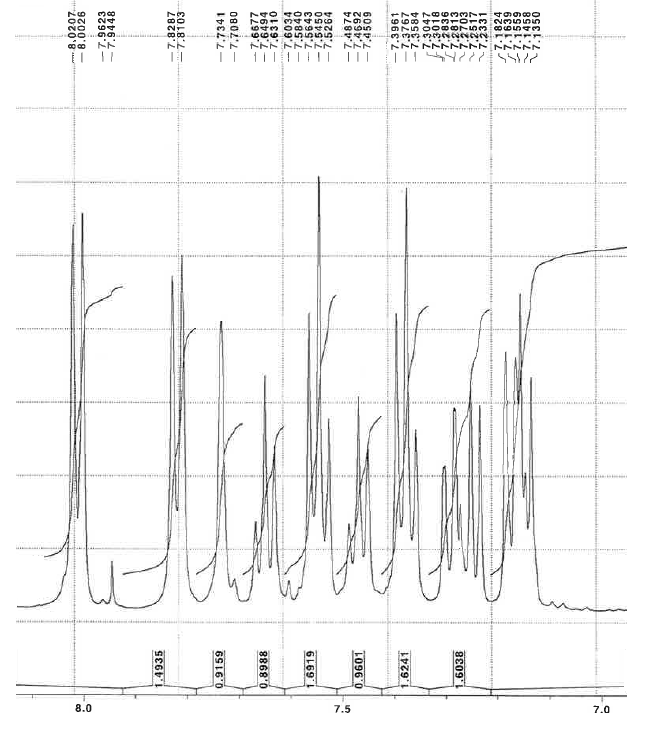
**

**Figure 45.^1^**H NMR spectrum of compound **6f** (from δ = 7 to 8.2).

**
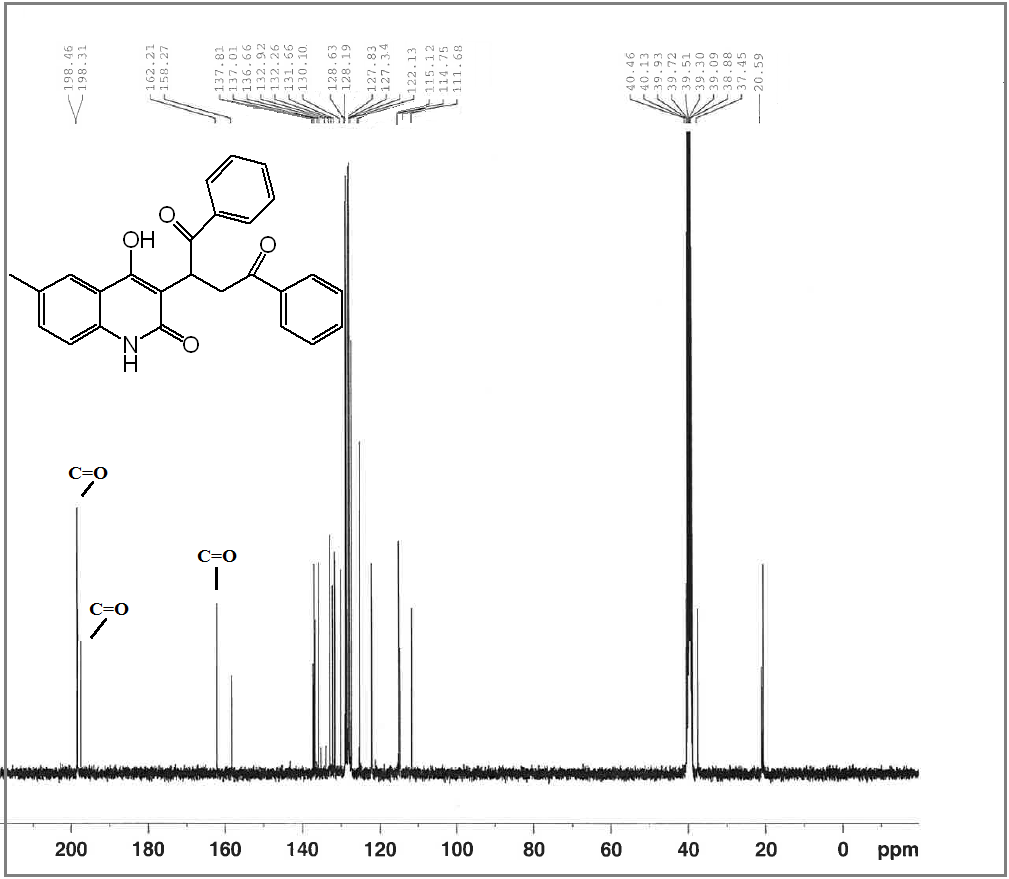
**

**Figure 46. ^13^C** NMR spectrum of compound **6f**.


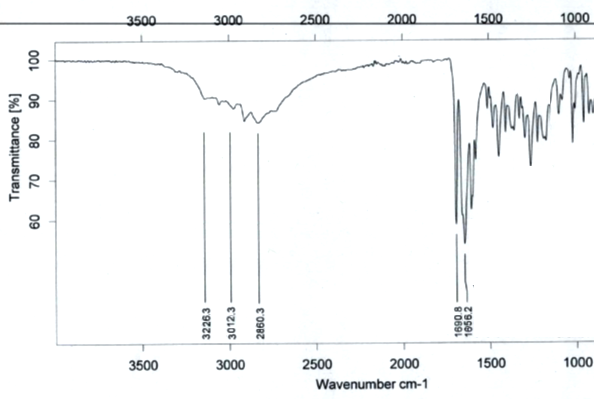


**Figure 47.** IR spectrum of compound **6f**.

**
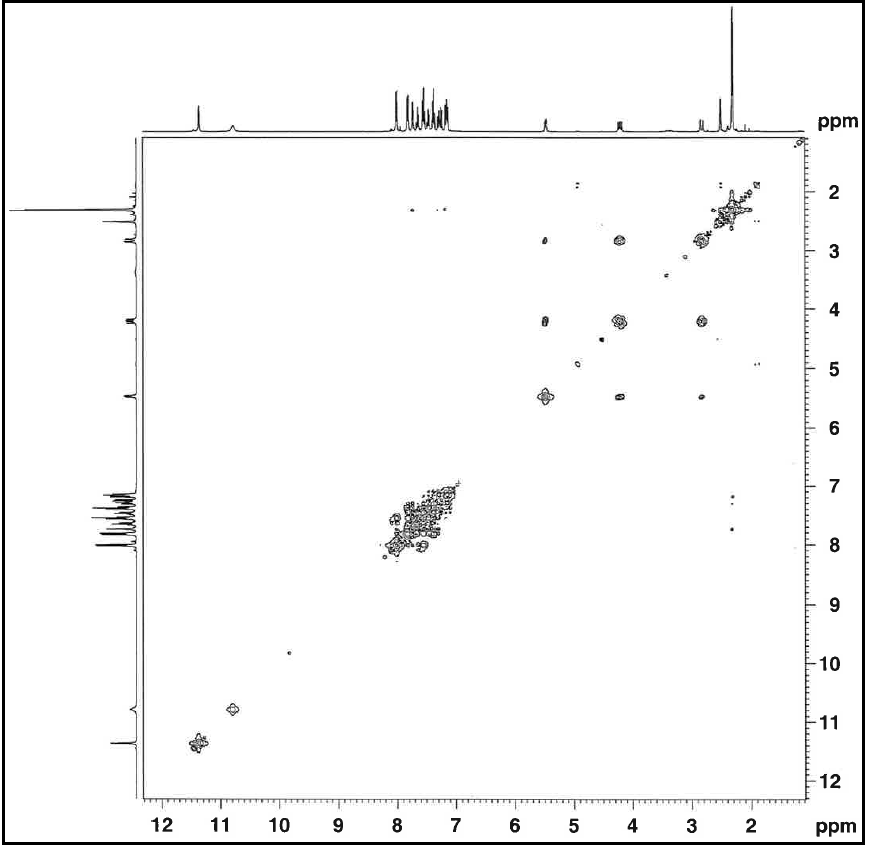
**

**Figure 48. ^1^H-^1^H** COSY NMR spectrum of compound **6f**.

**
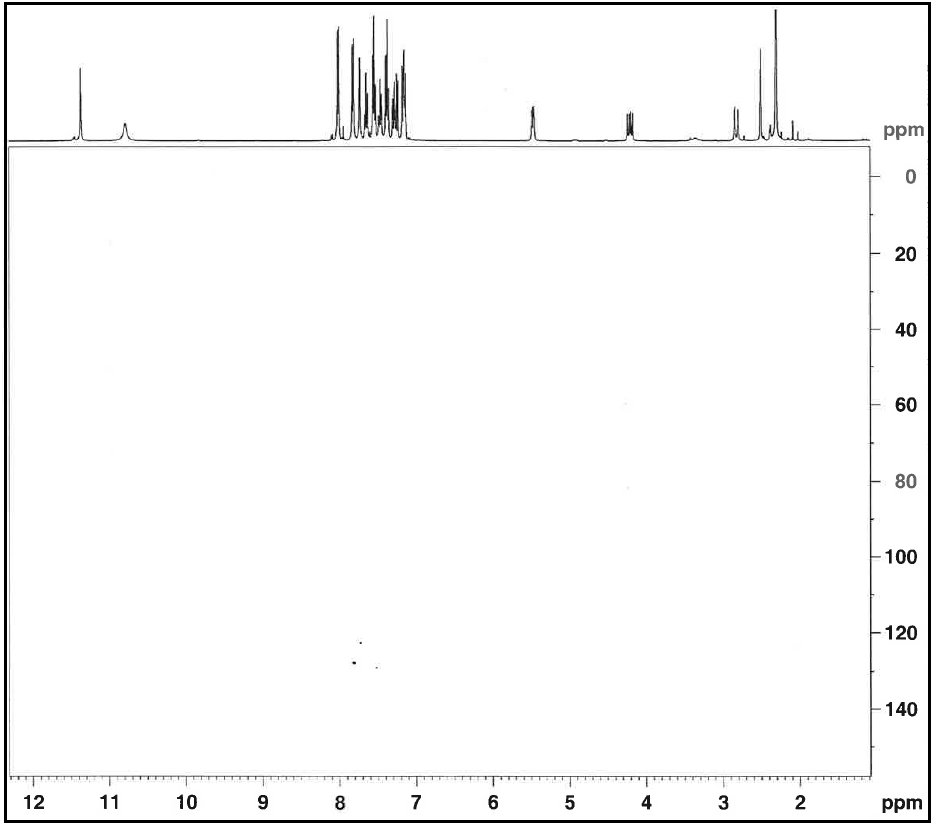
**

**Figure 49. ^1^H-^13^C** HSQC NMR spectrum of compound **6f**.

**
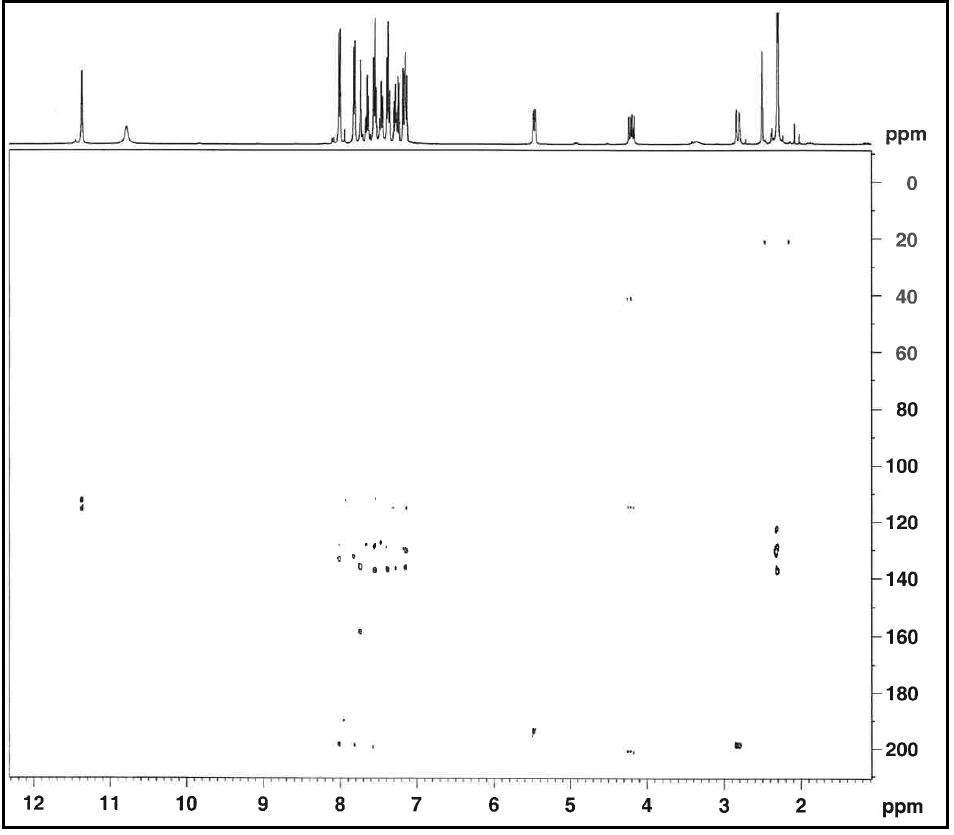
**

**Figure 50. ^1^H-^13^C** HMBC NMR spectrum of compound **6f**.

**
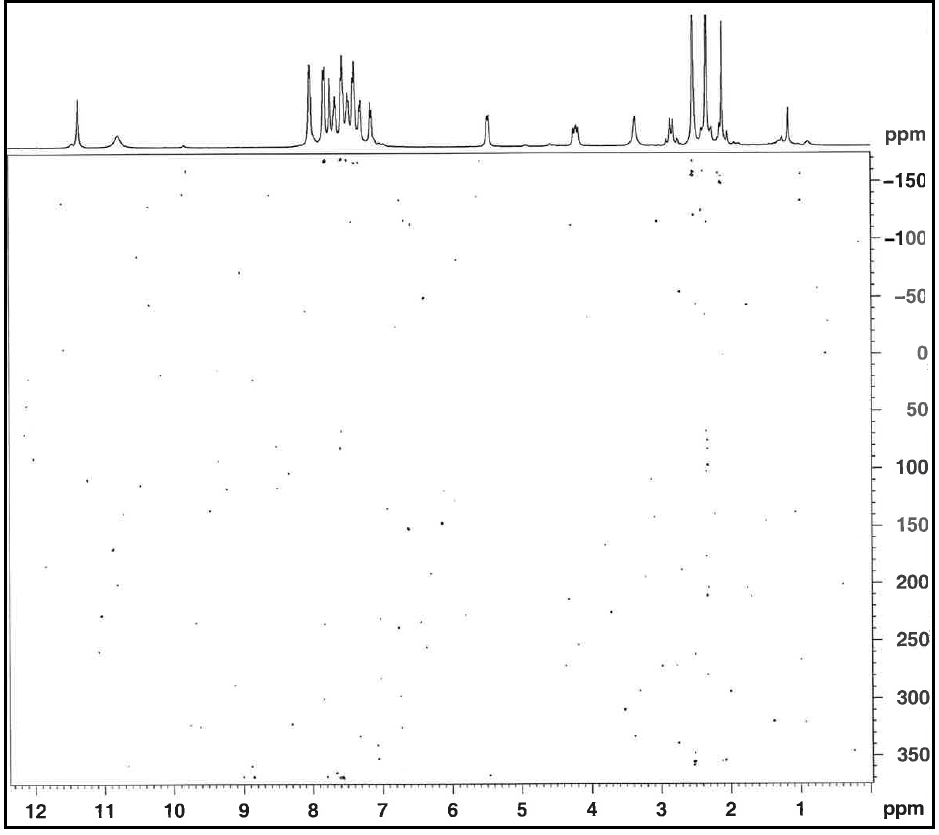
**

**Figure 51. ^1^H-^15^N** HSQC NMR spectrum of compound **6f**.

**
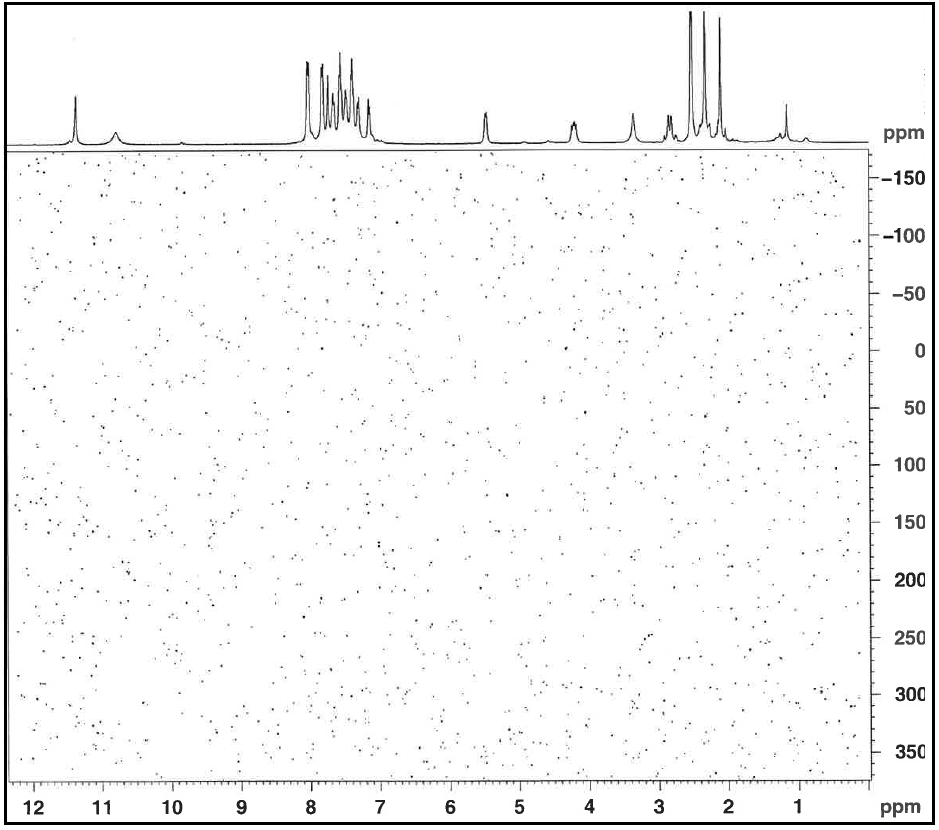
**

**Figure 52. ^1^H-^15^N** HMBC NMR spectrum of compound **6f**.

**
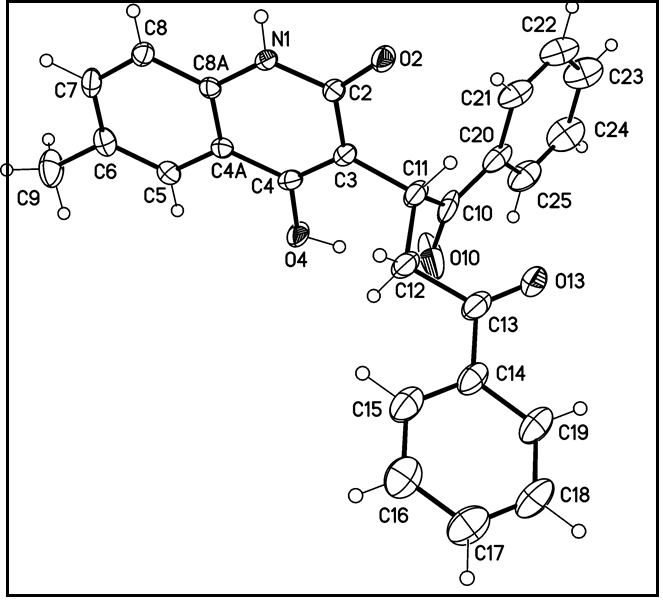
**

**Figure 53. X-ray** structure analysis of compound **6f**

**
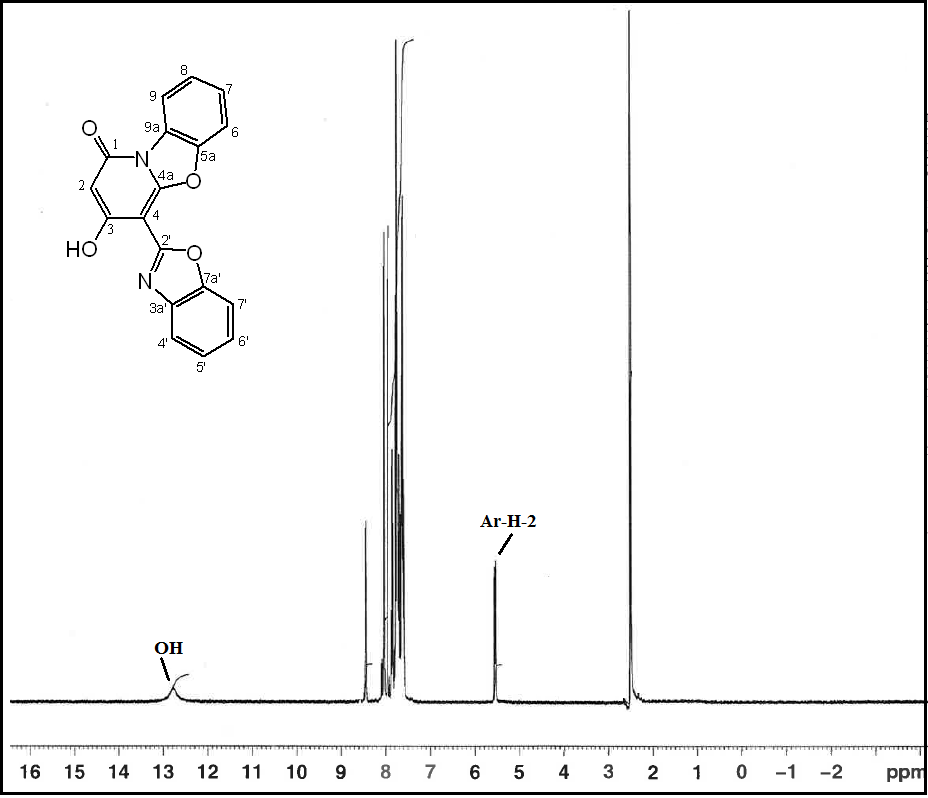
**

**Figure 54. ^1^H** NMR spectrum of compound **11**.

**
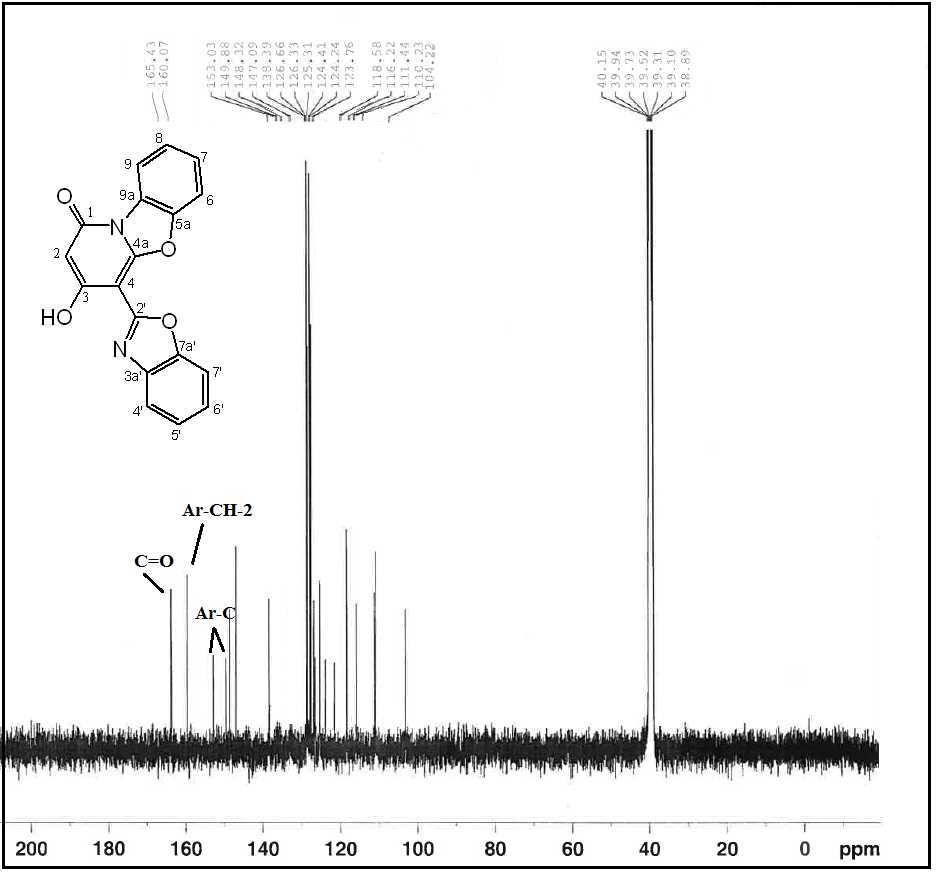
**

**Figure 55. ^13^C** NMR spectrum of compound **11**.

**
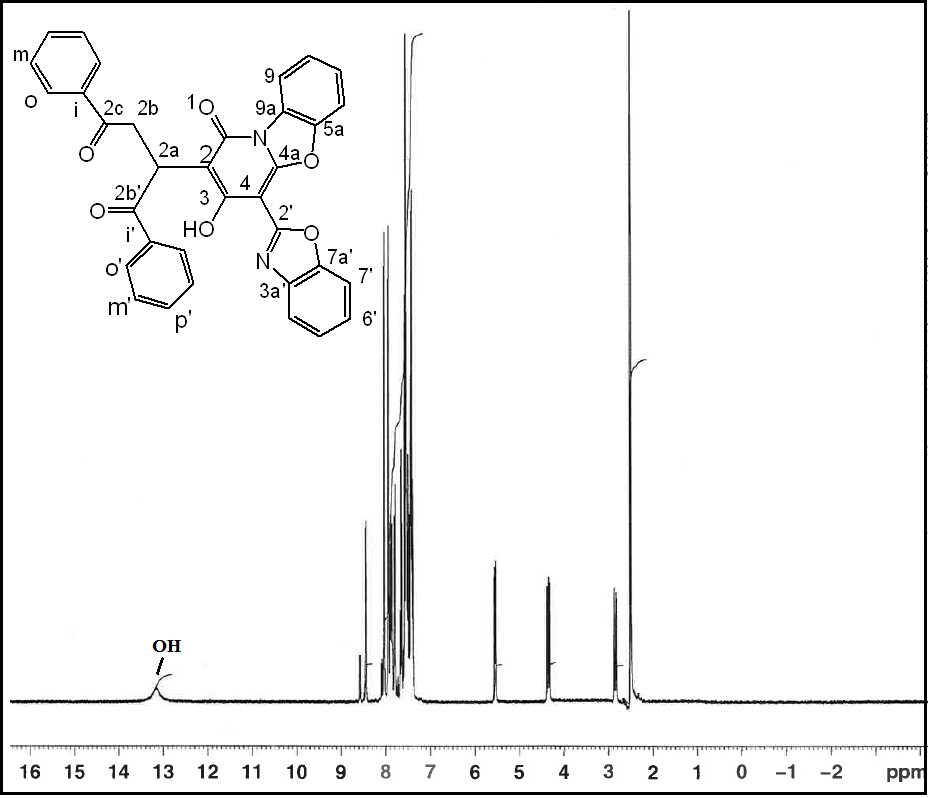
**

**Figure 56. ^1^H** NMR spectrum of compound **12**.

**
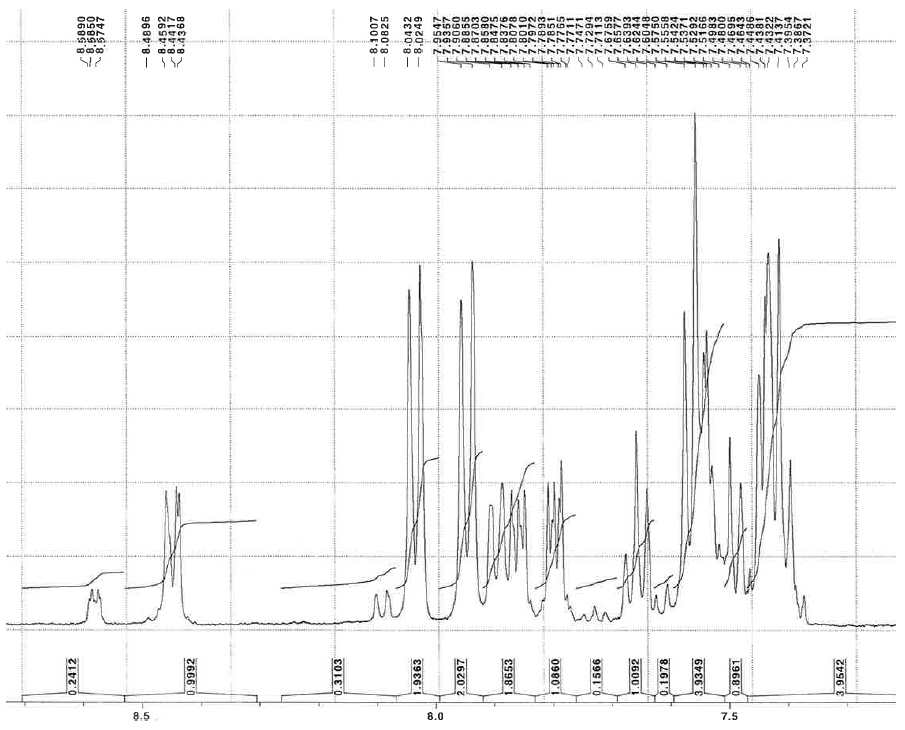
**

**Figure 57.^1^**H NMR spectrum of compound **12** (from δ = 7 to 8.2).

**
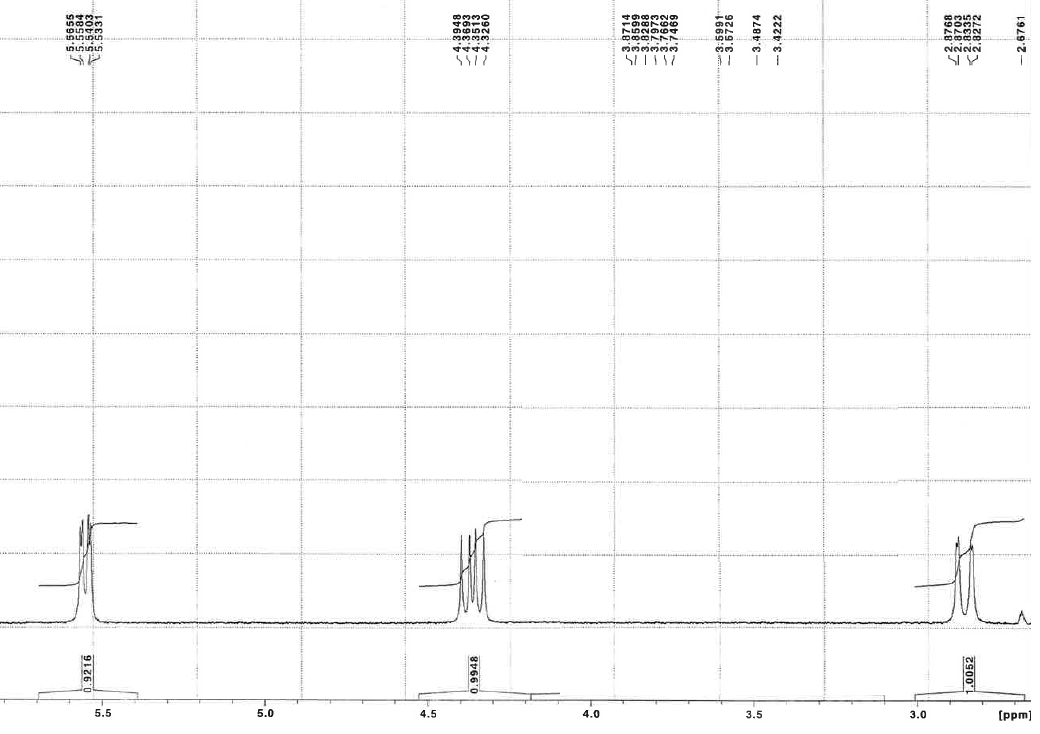
**

**Figure 58.^1^**H NMR spectrum of compound **12** (from δ = 2.5 to 5.8).

**
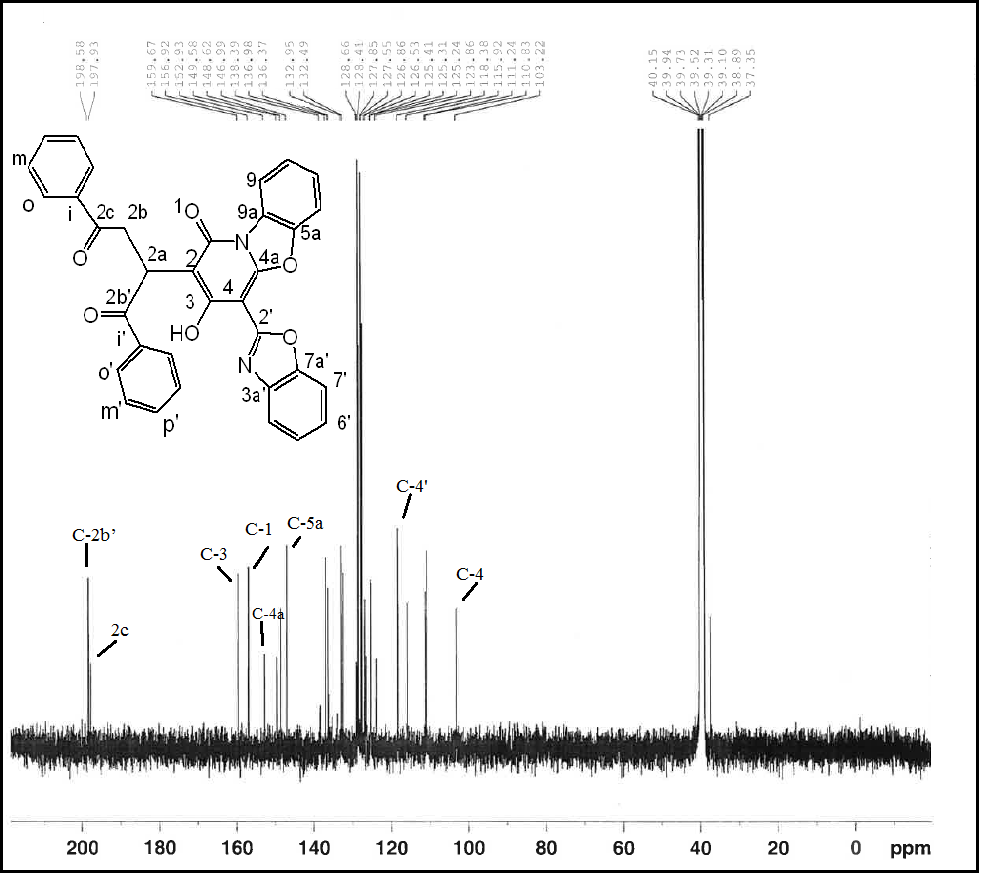
**

**Figure 59. ^13^C** NMR spectrum of compound **12**.


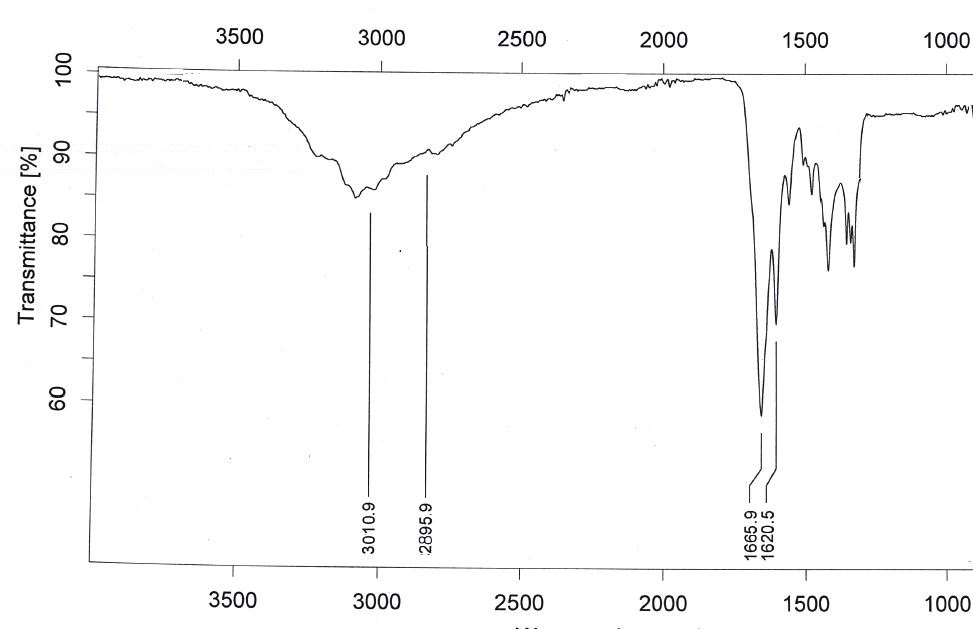


**Figure 60.** IR spectrum of compound **12**.

**
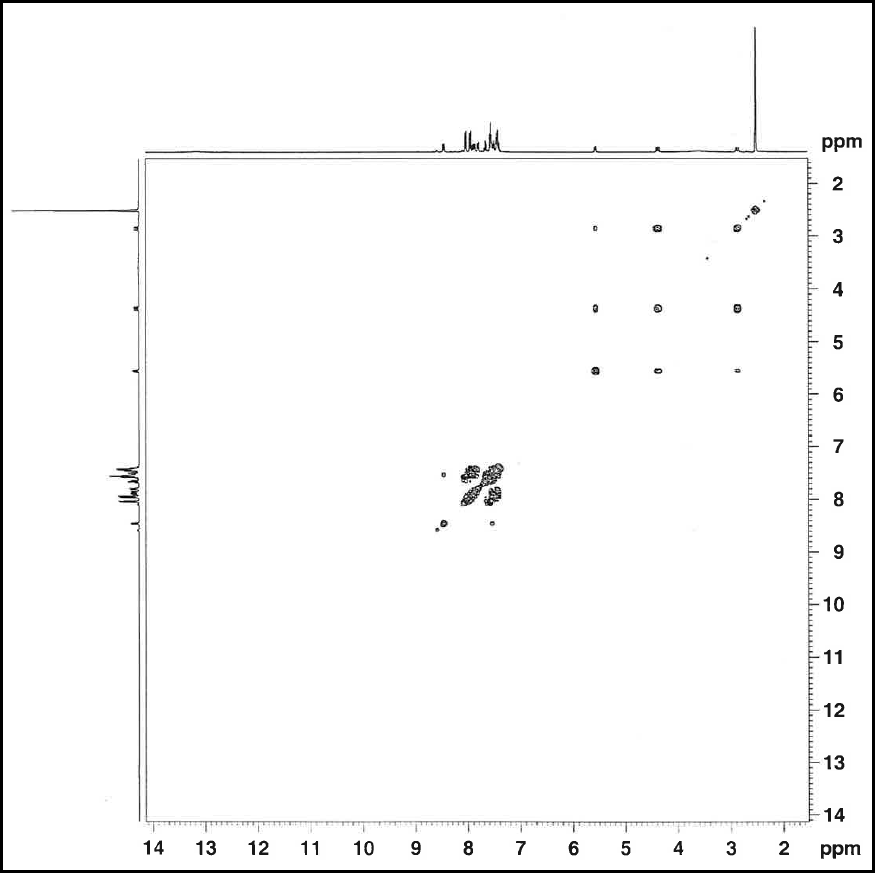
**

**Figure 61. ^1^H-^1^H** COSY NMR spectrum of compound **12**.

**
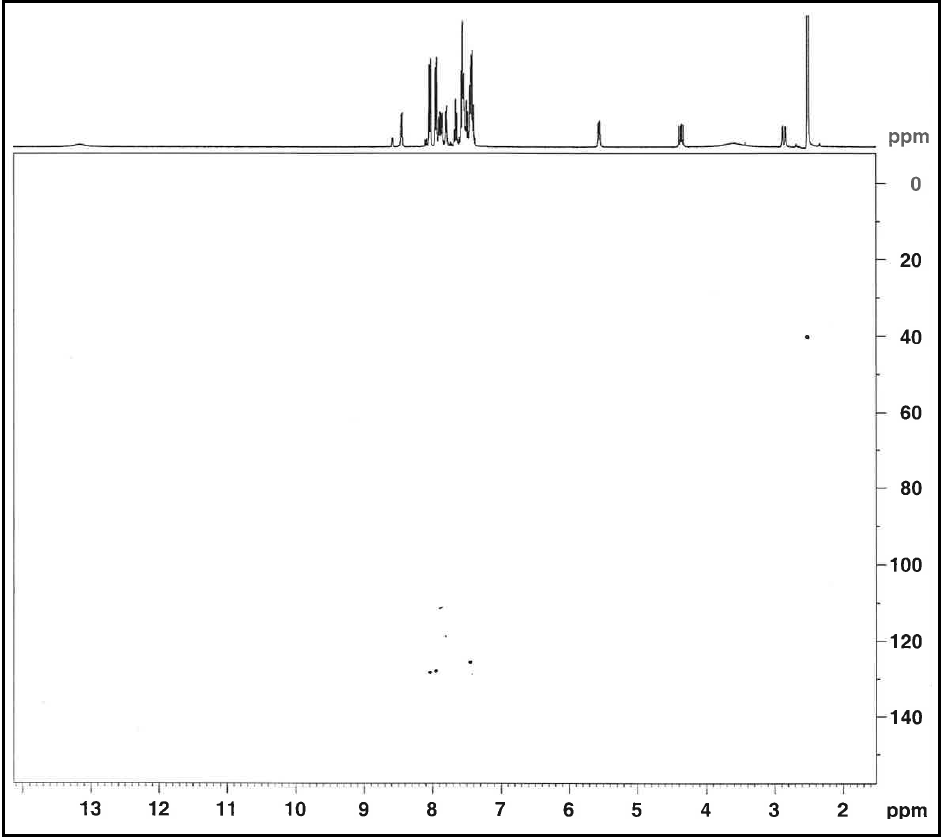
**

**Figure 62. ^1^H-^13^C** HSQC NMR spectrum of compound **12**.

**
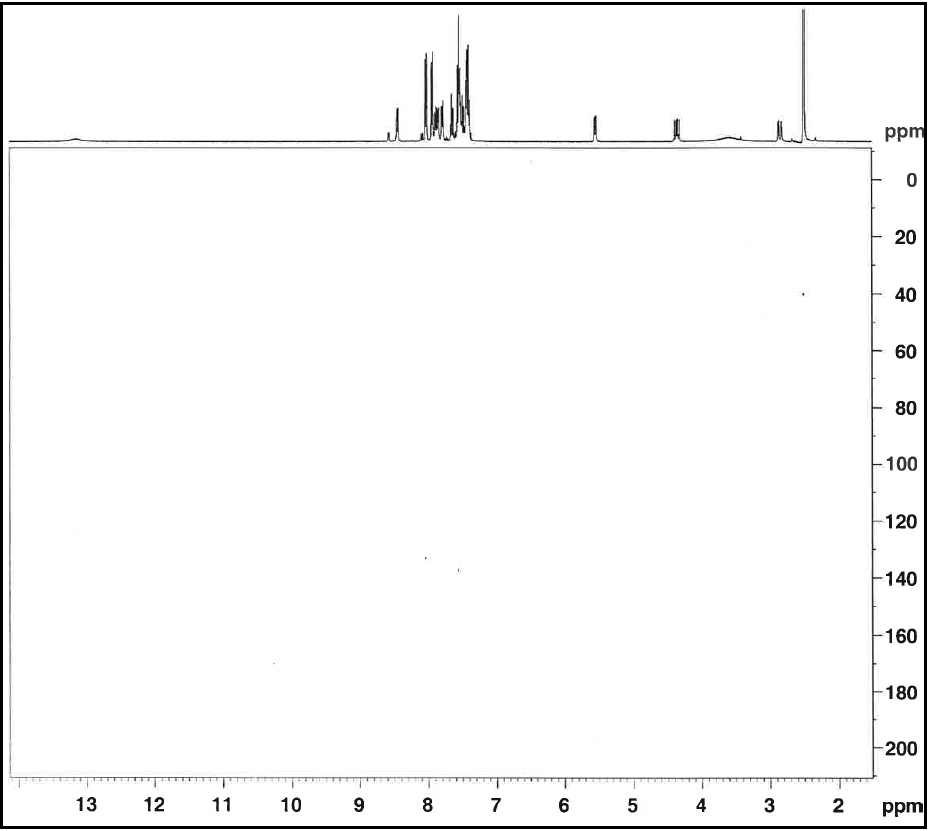
**

**Figure 63. ^1^H-^13^C** HMBC NMR spectrum of compound **12**.

**
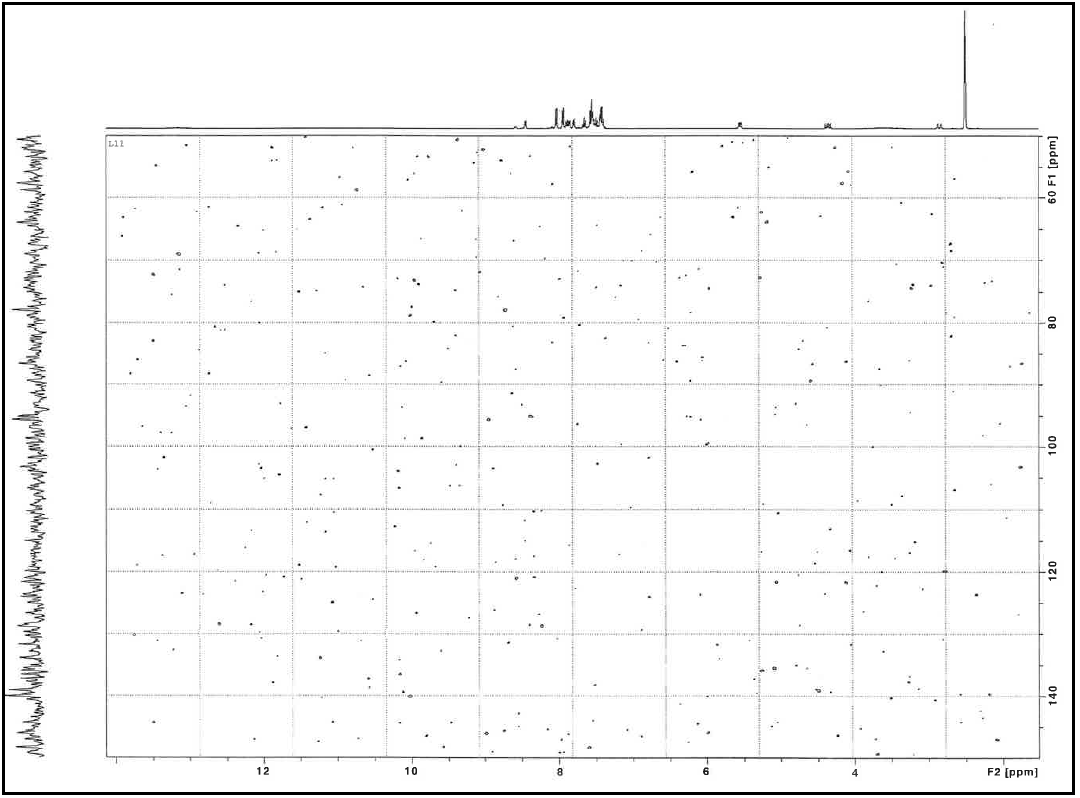
**

**Figure 64. ^1^H-^15^N** HSQC NMR spectrum of compound **12**.

**
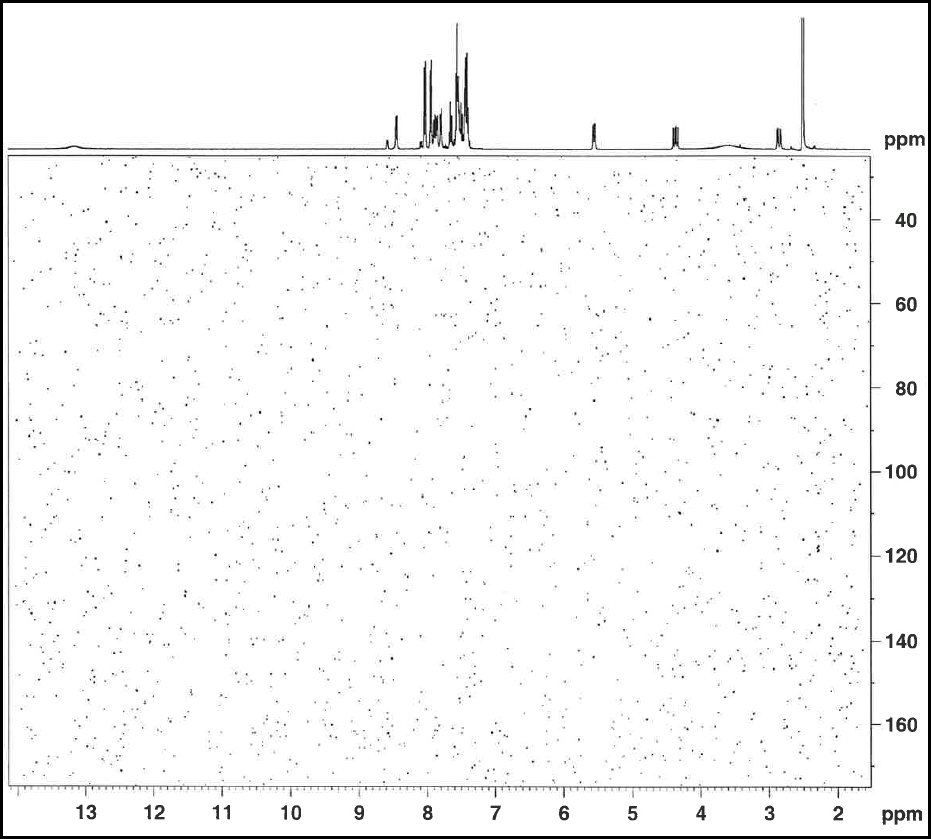
**

**Figure 65. ^1^H-^15^N** HMBC NMR spectrum of compound **12**.

**
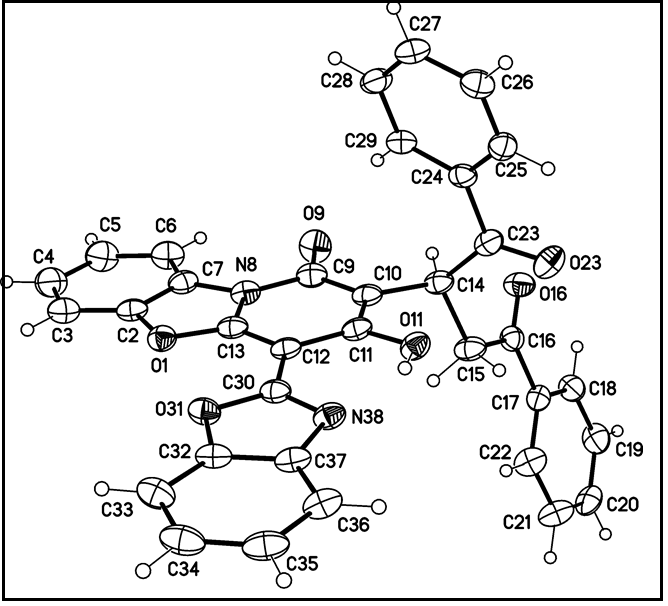
**

**Figure 66. X-ray** structure analysis of compound **12**.

**Table 1.** NMR spectroscopic data of compound **6f** (Bold-faced resonances are tall).

| **^1^H NMR (DMSO-*d*_6_): ^1^H-^1^H COSY: Assignment:** |
| --- |
| 11.35 (s; 1H) NH  10.77 (b; 1H) OH  8.01 (dd, *J* = 8.5, 1.3; 2H) 7.55 H-*o’*  7.81 (d, *J* = 7.3; 2H) 7.38 H-*o*  7.73 (bs; 1H) *7.29, 2.31* H-5  7.65 (t, *J* = 7.4; 1H) 7.55 H-*p’*  7.55 (“t”, *J* = 7.6; 2H) 8.01, 7.65 H-*m’*  7.47 (t, *J* = 7.3; 1H) 7.38 H-*p*  7.38 (“t”, *J* = 7.6; 2H) 7.81, 7.47 H-*m*  7.29 (dd, *J =* 8.4, 1.3; 1H) *7.73,* 7.14, *2.31* H-7  7.14 (d, *J* = 8.3; 1H) 7.29 H-8  5.46 (dd, *J* = 9.7, 3.1; 1H) 4.19, 2.82 H-α  4.19 (dd, *J* = 17.3, 9.8; 1H) 5.46, 2.82 H-α’  2.82 (dd, *J* = 17.3, 3.1; 1H) 5.46, 4.19 H-α’  2.31 (s; 3H) *7.73, 7.29* H-6a |
| **^15^N NMR (DMSO-*d*_6_): HSQC: HMBC: Assignment:** |
| 142.1 11.35 7.14 N-1 |
| **^13^C NMR (DMSO-*d*_6_): HSQC: HMBC: Assignment:** |
| 198.46, 198.31 8.01, 7.81, 7.38, 5.46, 4.19, 2.82 C-β,β’  162.21 5.46, *2.82* C-2  158.27 7.75, 7.14, 5.46 C-4  137.81, 137.01 7.55, *7.47,* 7.38 C-*i, i’*  136.66 *11.35,* 7.73, 7.29, *2.31* C-8a  132.92 7.65 8.01 C-*p’*  132.26 7.47 7.81 C-*p*  131.66 7.29 7.73 C-7  130.10 7.14, 2.31 C-6  **128.63** 7.55 7.55 C-*m’*  **128.19** 7.38 7.38 C-*m*  **127.83** 8.01 8.01, 7.65 C-o’  **127.34** 7.81 7.81, 7.47 C-*o*  122.13 7.73 2.31 C-5  115.12 7.14 C-8  114.75 11.35, 7.14 C-4a  111.68 5.46, 4.19, 2.82 C-3  40.46 5.46 4.19, 2.82 C-α  37.45 4.19, 2.82 5.46 C-α’  20.59 2.31 7.74, 7.29 C-6a |

**Table 2.** NMR spectroscopic data of compound **12** (Bold-faced resonances are tall.)

| **^1^H NMR (DMSO-*d_6_*): ^1^H-^1^H COSY: Assignment:** |
| --- |
| 13.16 (b; 1H) OH  8.45 (dd, *J* = 7.0, 2.0; 1H) 7.52 H-9  8.03 (d, *J* = 7.3; 2H) 7.56 H-*o/o’*  7.95 (d, *J* = 7.6; 2H) 7.41 H-*o’/o*  7.90 (bd, *J* = 8.2; 1H) 7.54 H-6  7.86 (m; 1H) 7.43 H-7’  7.80 (m; 1H) 7.43 H-4’  7.66 (t, *J* = 7.3; 1H) 7.56 H-*p/p’*  7.56 (“t”: *J* = 7.6; 2H) 8.03, 7.66 H-*m/m’*  7.54 (m; 1H) 7.90 H-7  7.52 (m; 1H) 8.45 H-8  7.49 (t, *J* = 7.3; 1H) 7.41 H-*p’/p*  7.43 (m; 2H) 7.86, 7.80 H-5’, 6’  7.41 (“t”, *J* = 7.3; 2H) 7.95, 7.49 H-*m’/m*  5.55 (dd, *J* = 10.1, 2.9; 1H) 4.36, 2.85 H-2a  4.36 (dd, *J* = 17.4, 10.2; 1H) 5.55, 2.85 H-2b  2.85 (dd, *J* = 17.2, 2.6; 1H) 5.55, 4.36 H-2b |
| **^13^C NMR (DMSO-*d*_6_): ^1^H-^13^C HSQC: ^1^H-^13^C HMBC: Assignment:** |
| 198.58 8.03 C-2c/2b’  197.93 7.95 C-2b’/2c  159.67 C-3  156.92 5.55 C-1  152.93 C-4a  149.58 C-2’  148.62 7.43 C-7a’  146.99 C-5a  138.39 C-3a’  136.98, 136.37 7.56, 7.41 C-*i, i’*  132.94 7.66 8.03 C-*p/p’*  132.49 7.49 7.95 C-*p’/p*  **128.66** 7.56 7.56 C-*m/m’*  **128.41** 7.41 7.41 C-*m’/m*  **127.85** 8.03 8.03, 7.66 C-*o/o’*  **127.55** 7.95 7.95, 7.49 C-*o’/o*  126.86, 126.53 7.54 8.45, 7.90, 7.86, 7.80 C-7, 9a  125.41, 125.31, 125.24 7.52, 7.43 C-5’, 6’, 8  123.86 C-2  118.38 7.80 C-4’  115.92 8.45 C-9  111.24 7.90 C-6 |
| 110.83 7.86 7.43 C-7’  103.22 C-4  39.5 5.55 4.36 C-2a  37.35 4.36, 2.85 C-2b |
