# Supplementary material for: Synthesis of New Fused Heterocyclic 2-Quinolones and 3-Alkanonyl-4-Hydroxy-2-Quinolones
Source: Molecules. 2019 Oct 21;24(20):3782. doi: 10.3390/molecules24203782 (PMC6832483; doi:10.3390/molecules24203782)
Supplement: Supplementary file 1 [file molecules-24-03782-s001.zip › Molecules_Aly_Quinolones_SI1.docx]

**Novel fused heterocyclic 2-quinolones and 3-alkanonyl-4-hydroxy-2-quinolone derivatives as prospective HIV-1 nonnucleoside reverse transcriptase inhibitors**

**Ashraf A. Aly,^a^* Alaa A. Hassan,^a^ Nasr K. Mohamed,^a^ Lamiaa E. Abd El-Haleem,^a^**

**Stefan Bräse,^b^ Mika Polamo,^c^ Martin Nieger,^c^ Alan B. Brown,^d^ Mahmoud A. A. Ibrahim^a^**


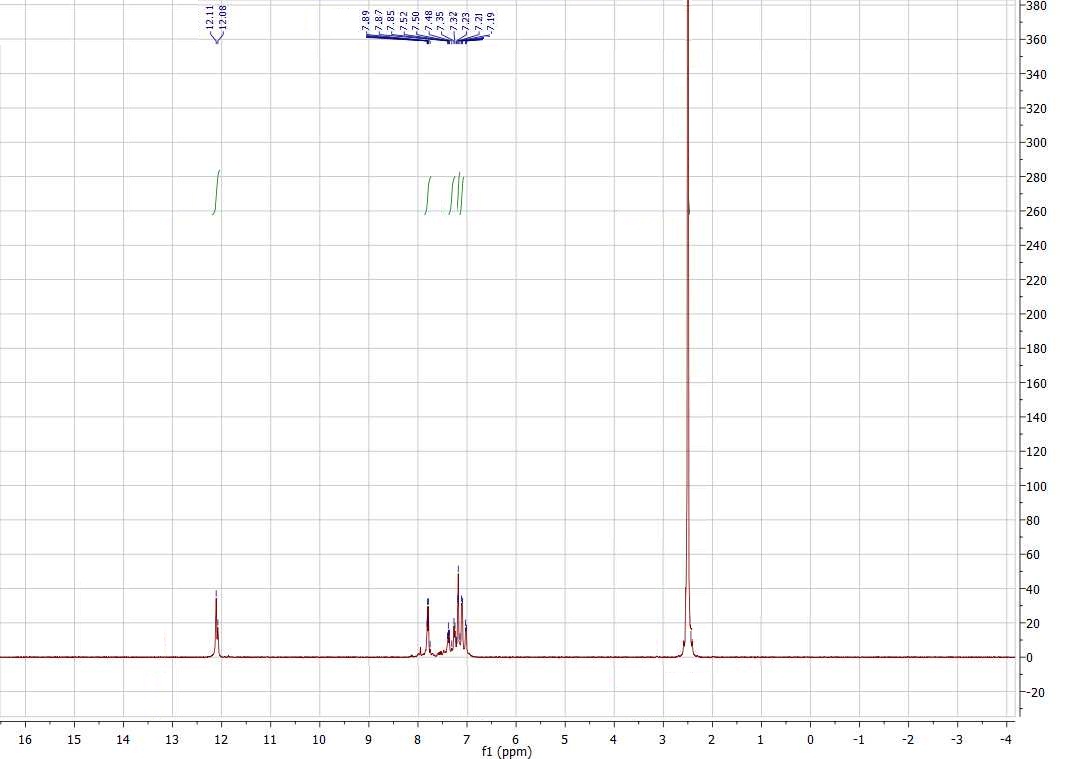


**Figure 1. ^1^**H NMR spectrum of compound **3a**

**
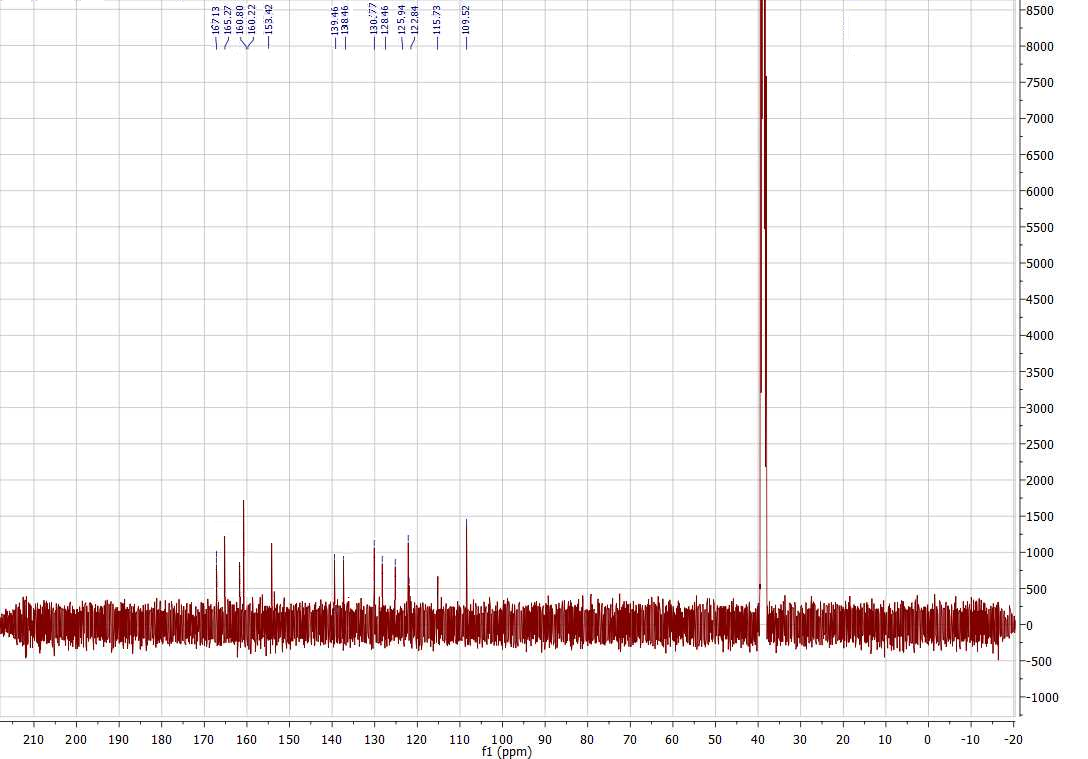
**

**Figure 2. ^13^C** NMR spectrum of compound **3a**

**
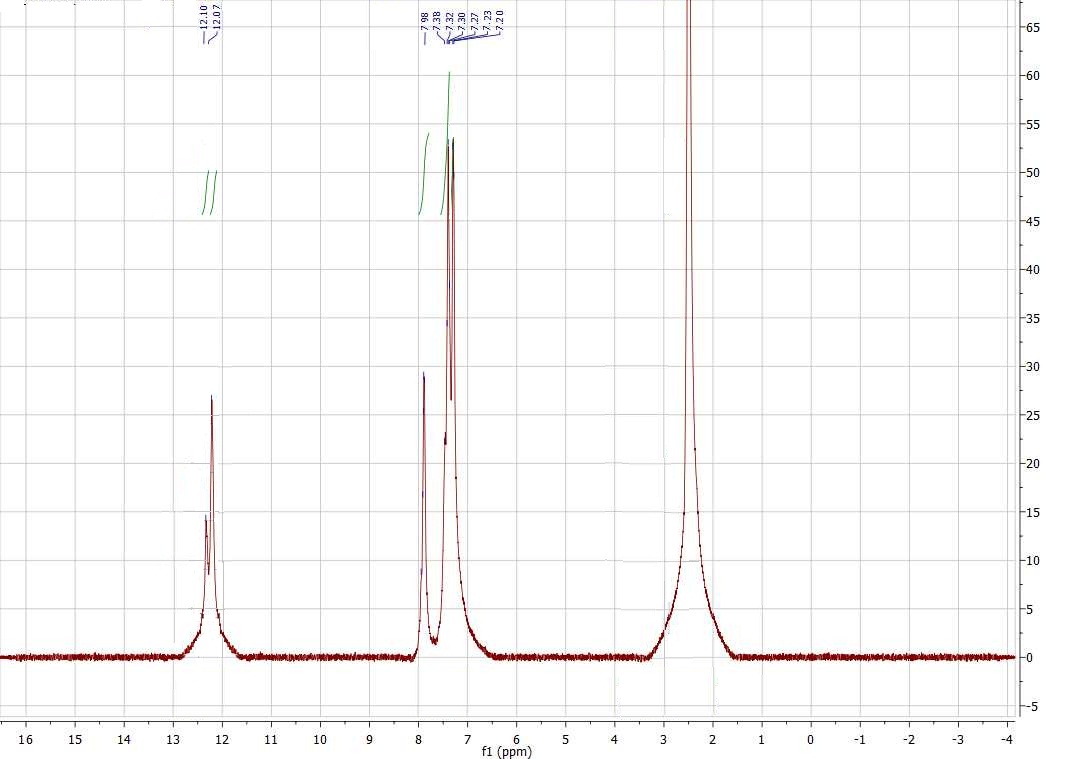
**

**Figure 3. ^1^**H NMR spectrum of compound **3b**

**
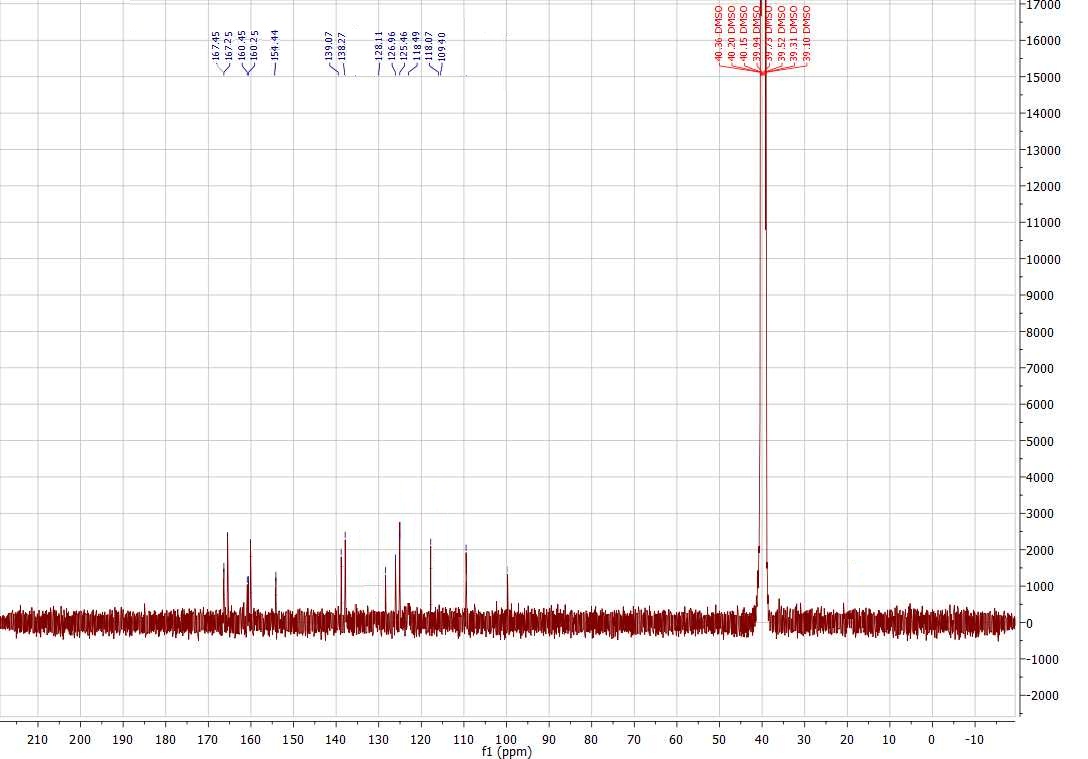
**

**Figure 4. ^13^C** NMR spectrum of compound **3b**

**
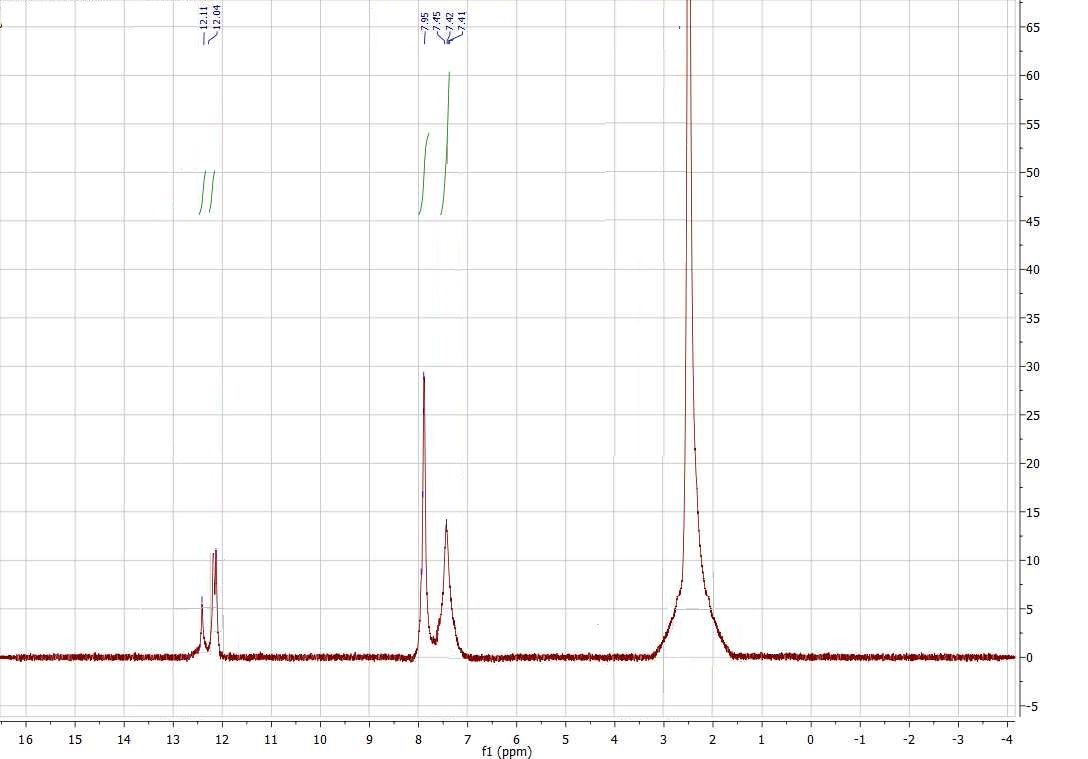
**

**Figure 5. ^1^**H NMR spectrum of compound **3c**

**
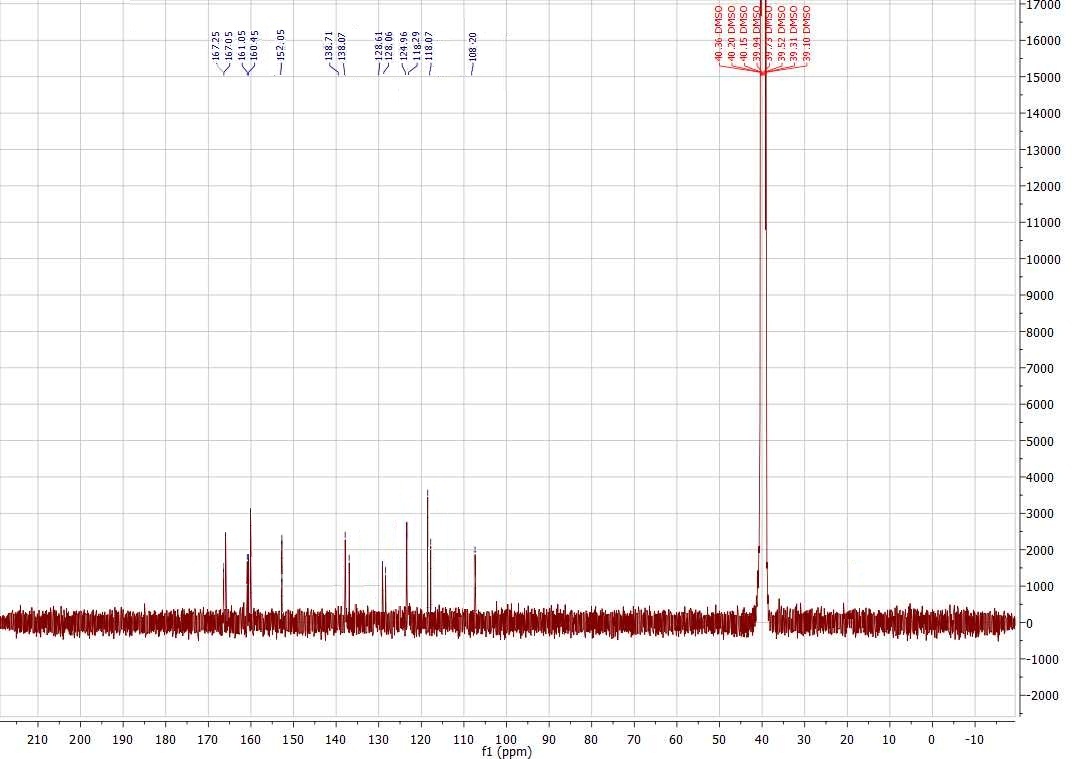
**

**Figure 6. ^13^C** NMR spectrum of compound **3c**

**
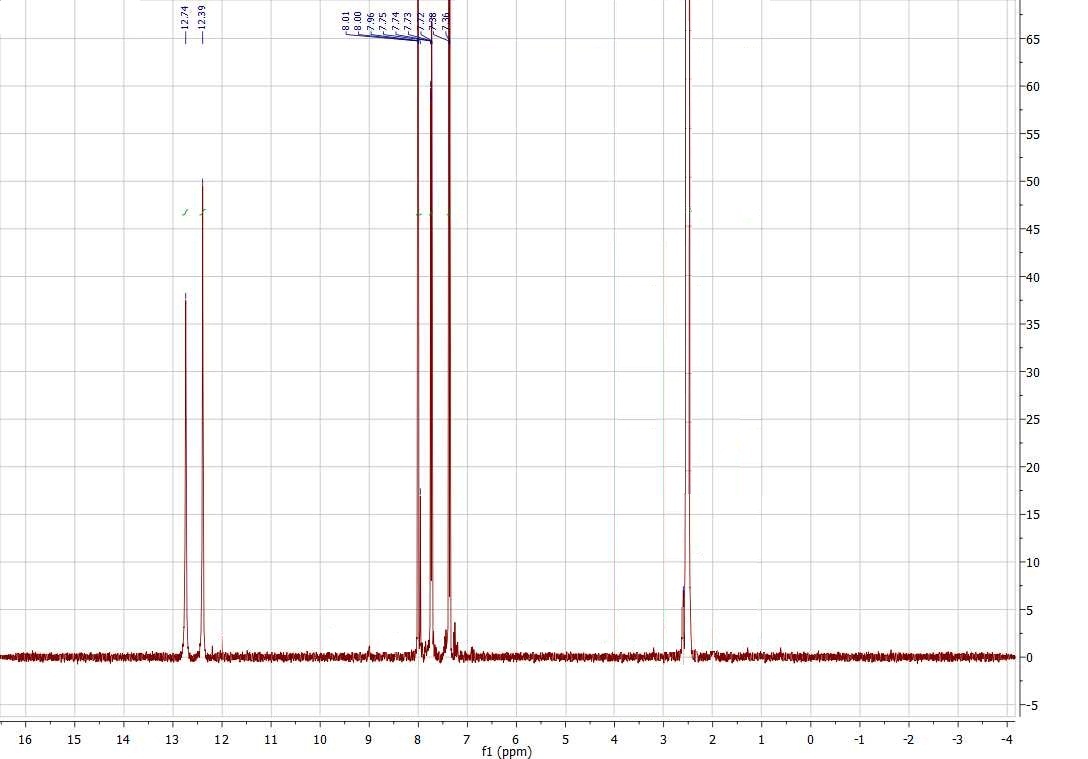
**

**Figure 7. ^1^**H NMR spectrum of compound **3d**

**
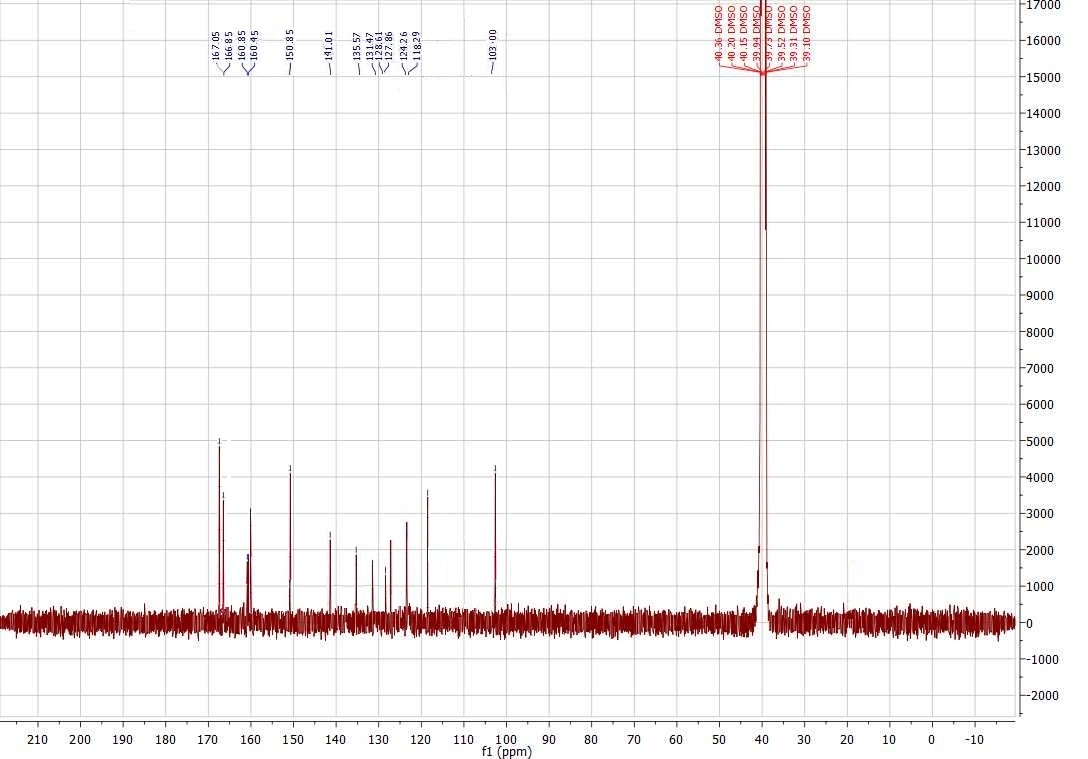
**

**Figure 8. ^13^C** NMR spectrum of compound **3d**

**
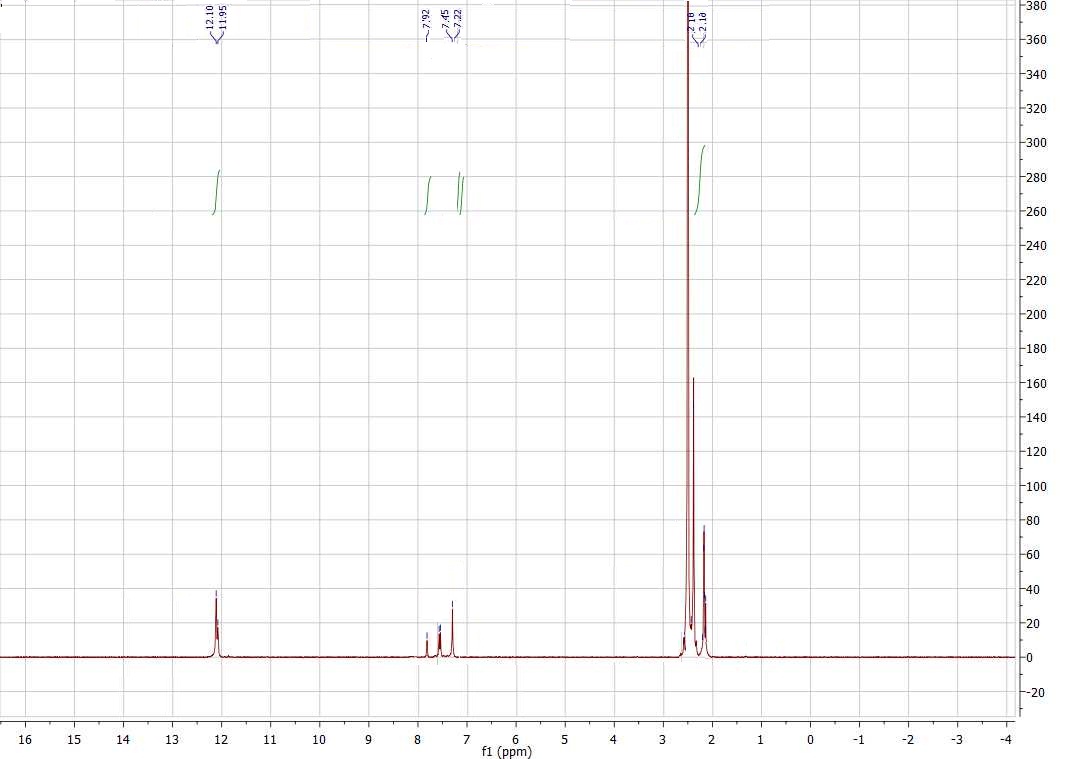
**

**Figure 9. ^1^**H NMR spectrum of compound **3e**

**
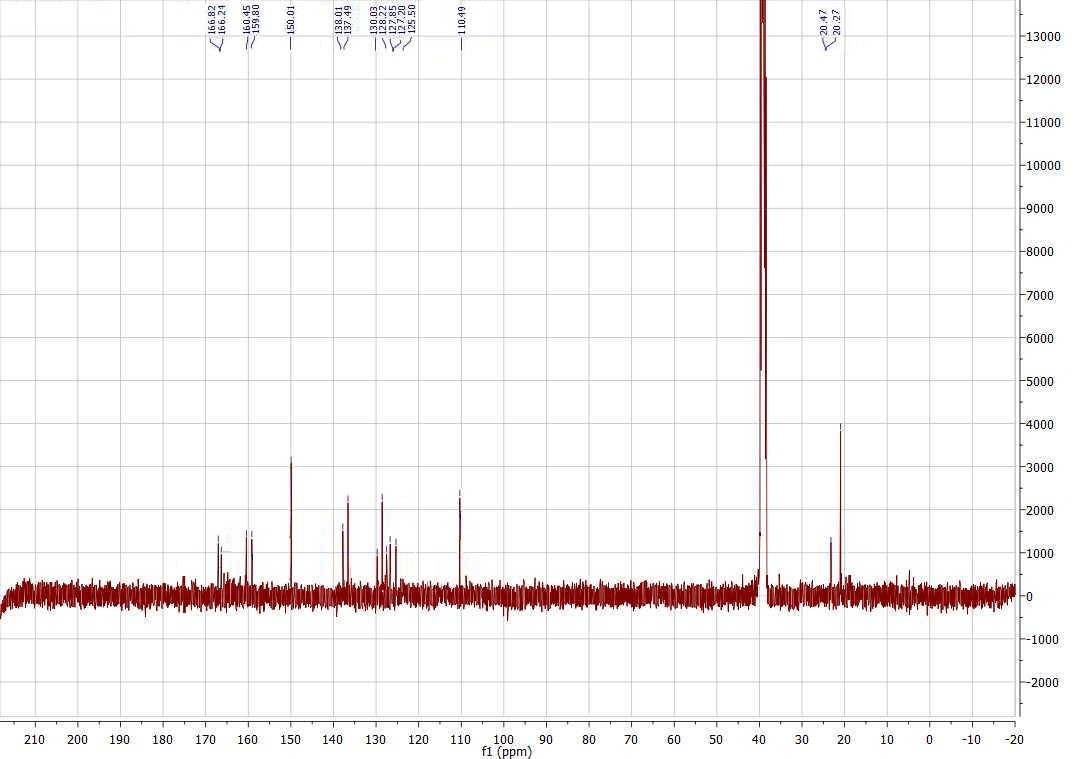
**

**Figure 10. ^13^C** NMR spectrum of compound **3e**

**
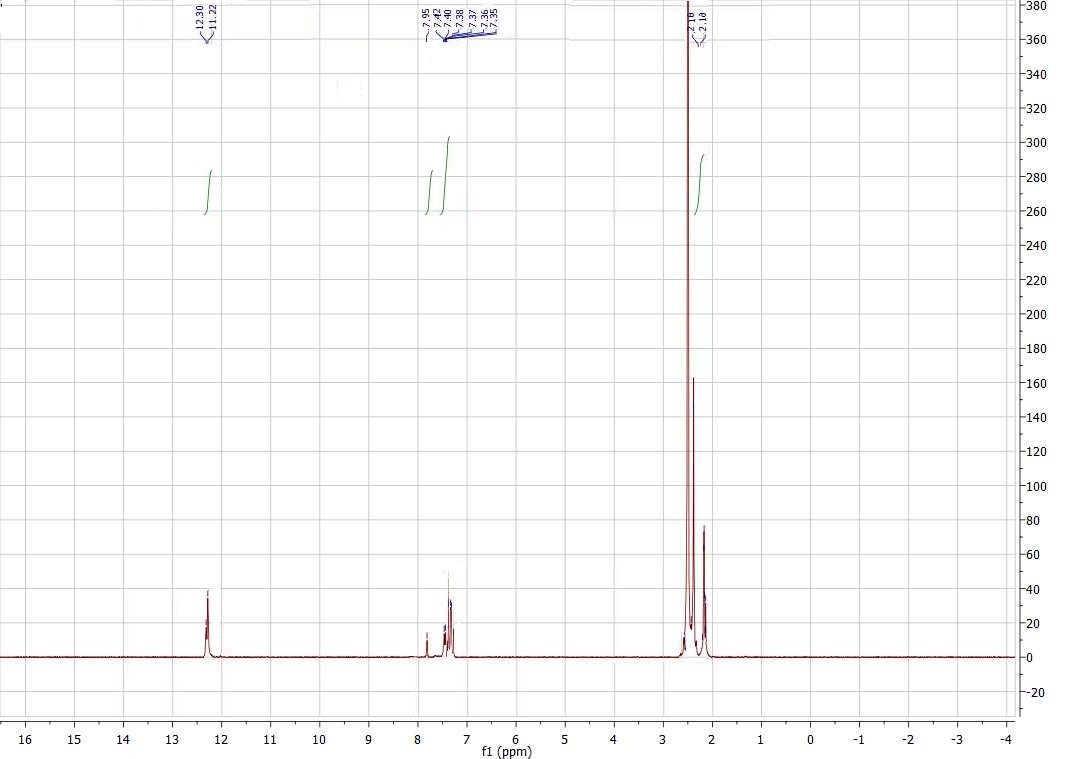
**

**Figure 11. ^1^**H NMR spectrum of compound **3f**

**
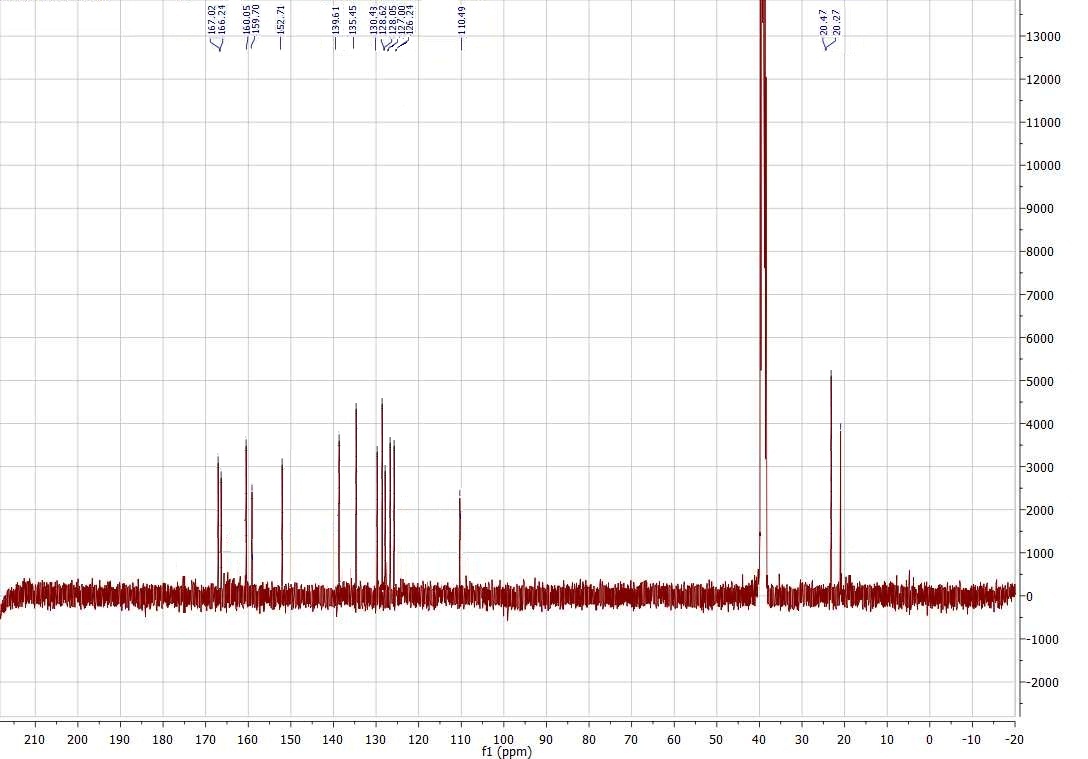
**

**Figure 12. ^13^C** NMR spectrum of compound **3f**

**
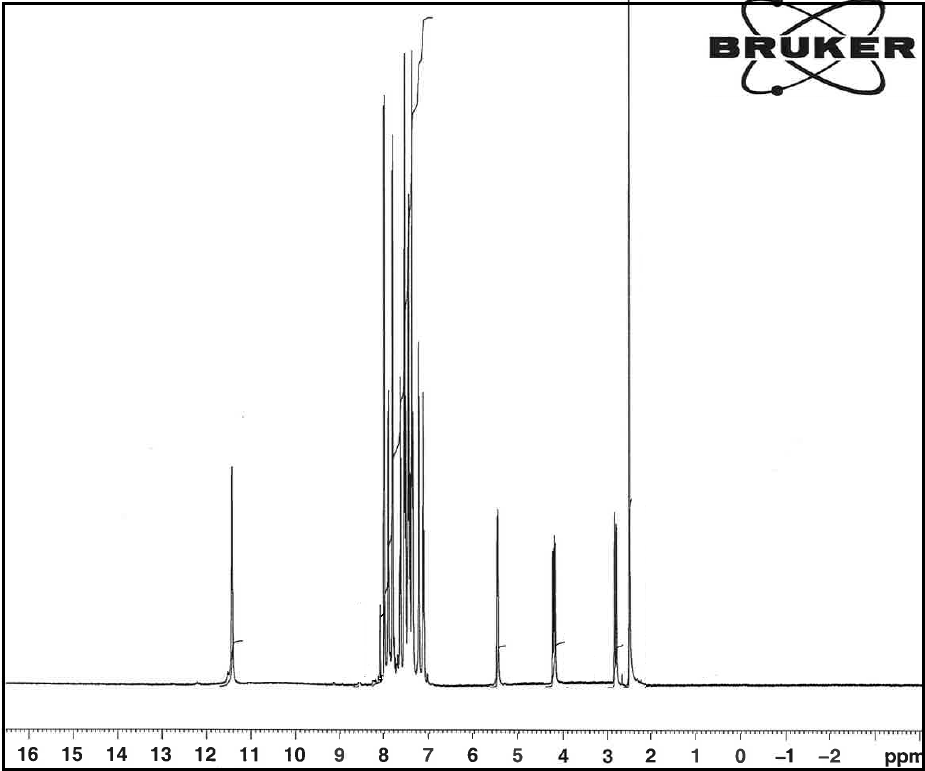
**

**Figure 13. ^1^**H NMR spectrum of compound **6a**

**
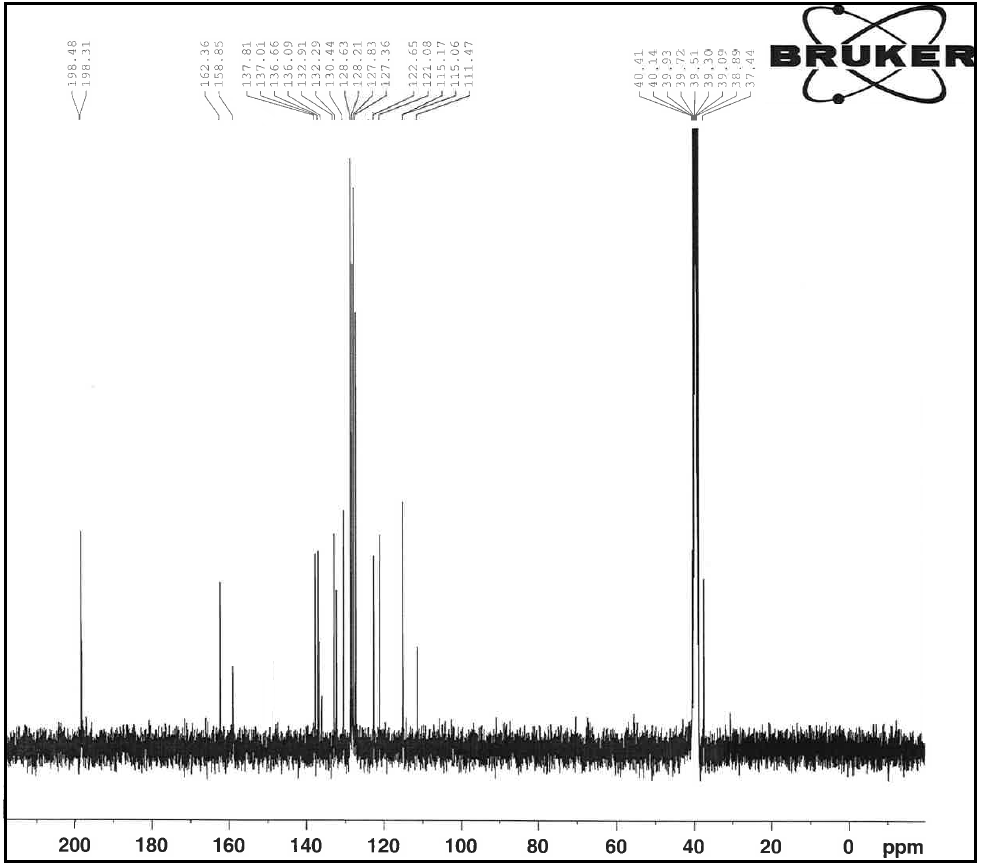
**

**Figure 14. ^13^C** NMR spectrum of compound **6a**

**
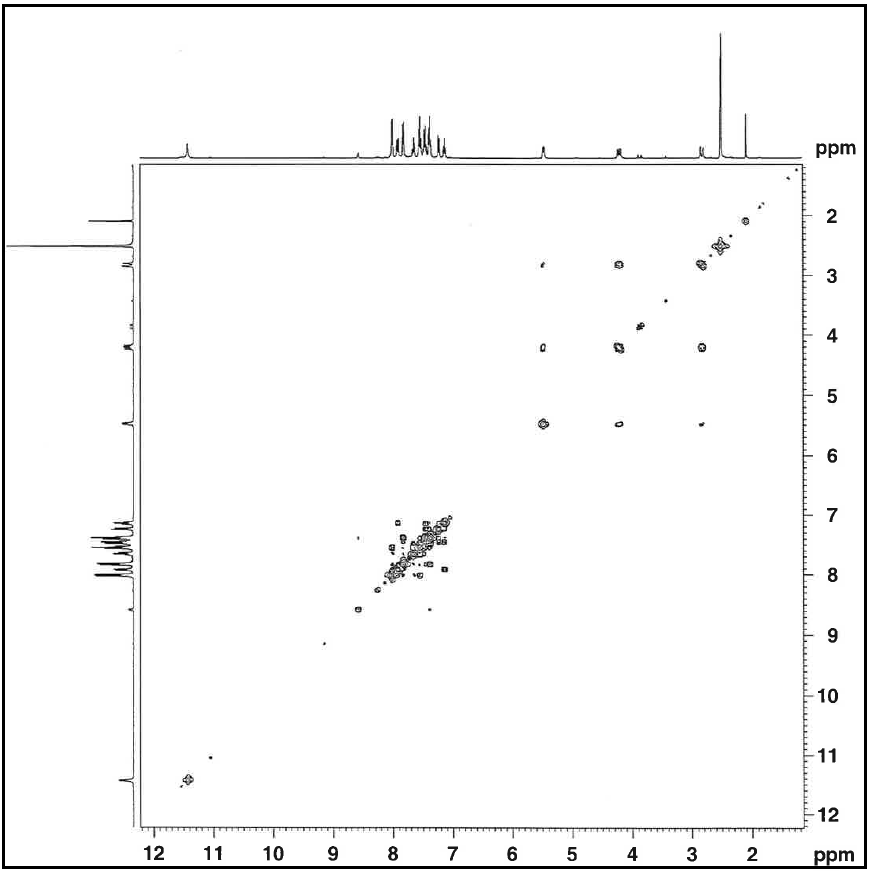
**

**Figure 15. ^1^H-^1^H** COSY NMR spectrum of compound **6a**

**
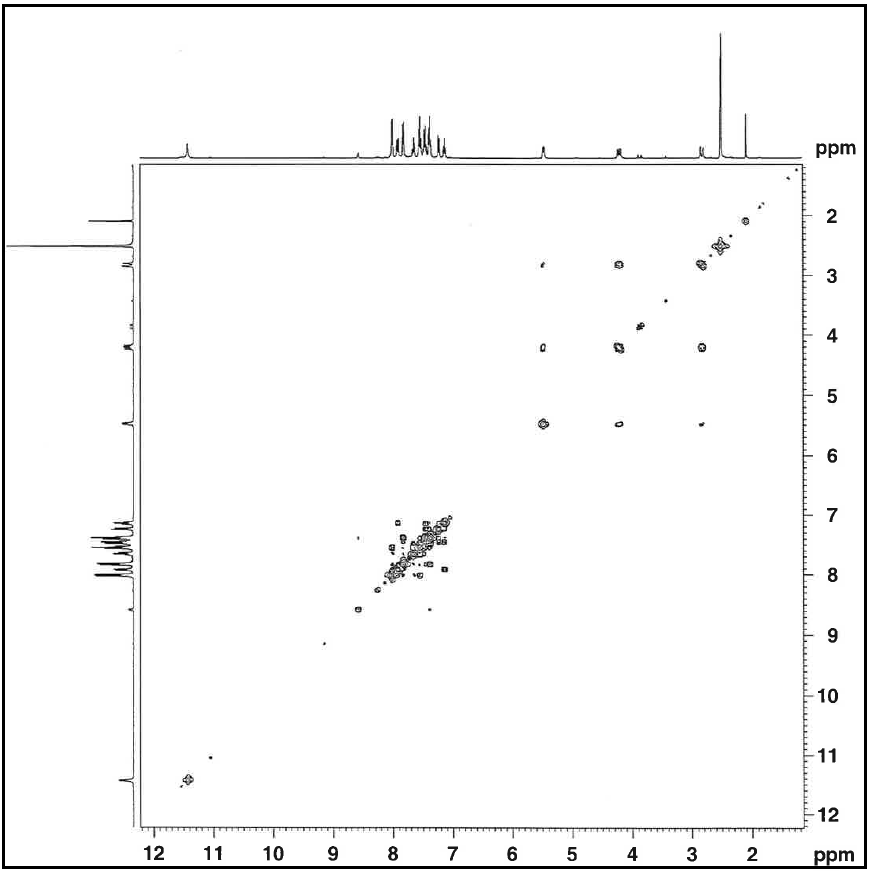
**

**Figure 16. ^1^H-^13^C** HSQC NMR spectrum of compound **6a**

**
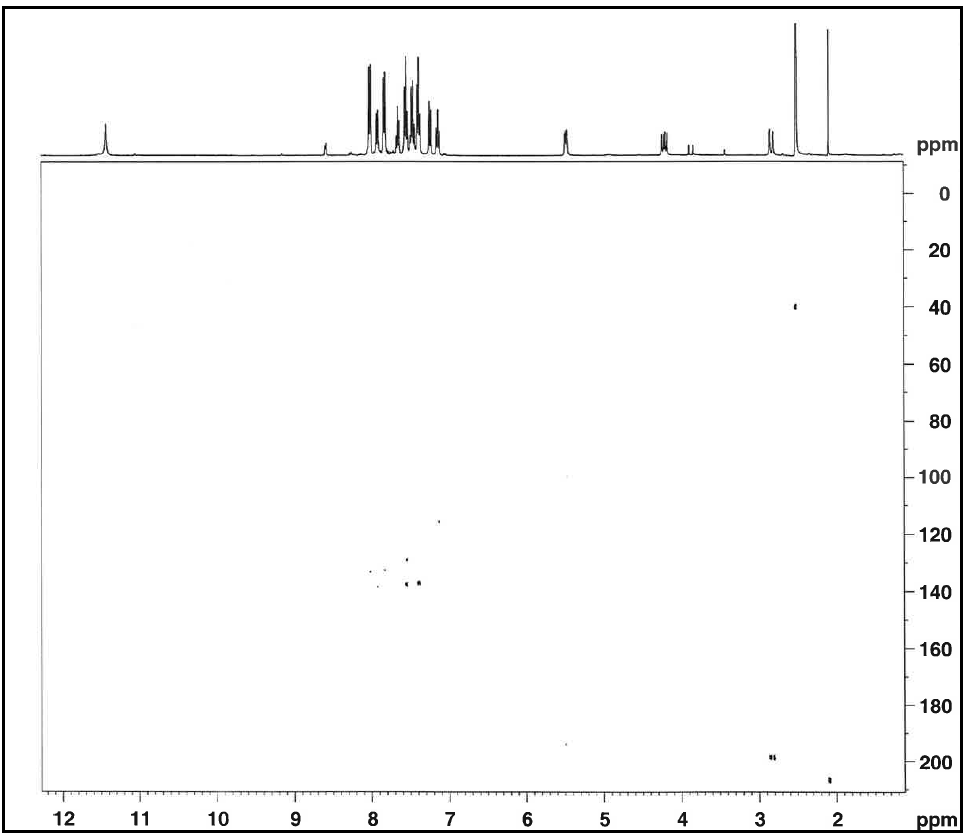
**

**Figure 17. ^1^H-^13^C** HMBC NMR spectrum of compound **6a**

**
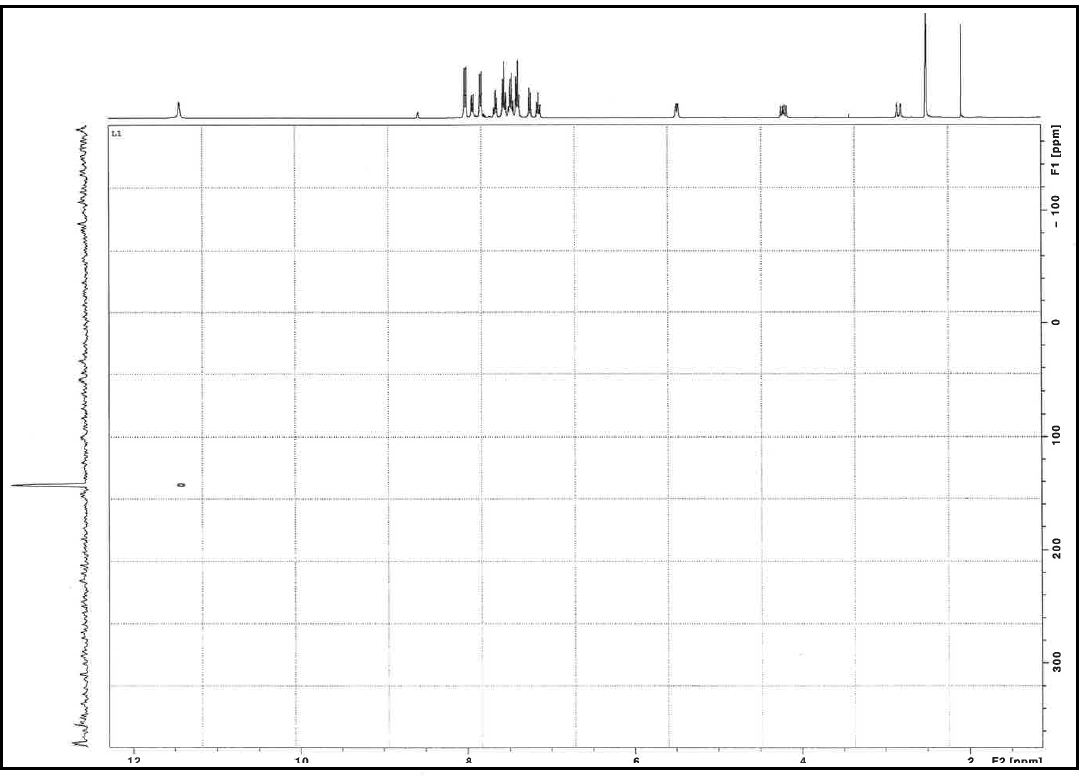
**

**Figure 18. ^1^H-^15^N** HSQC NMR spectrum of compound **6a**

**
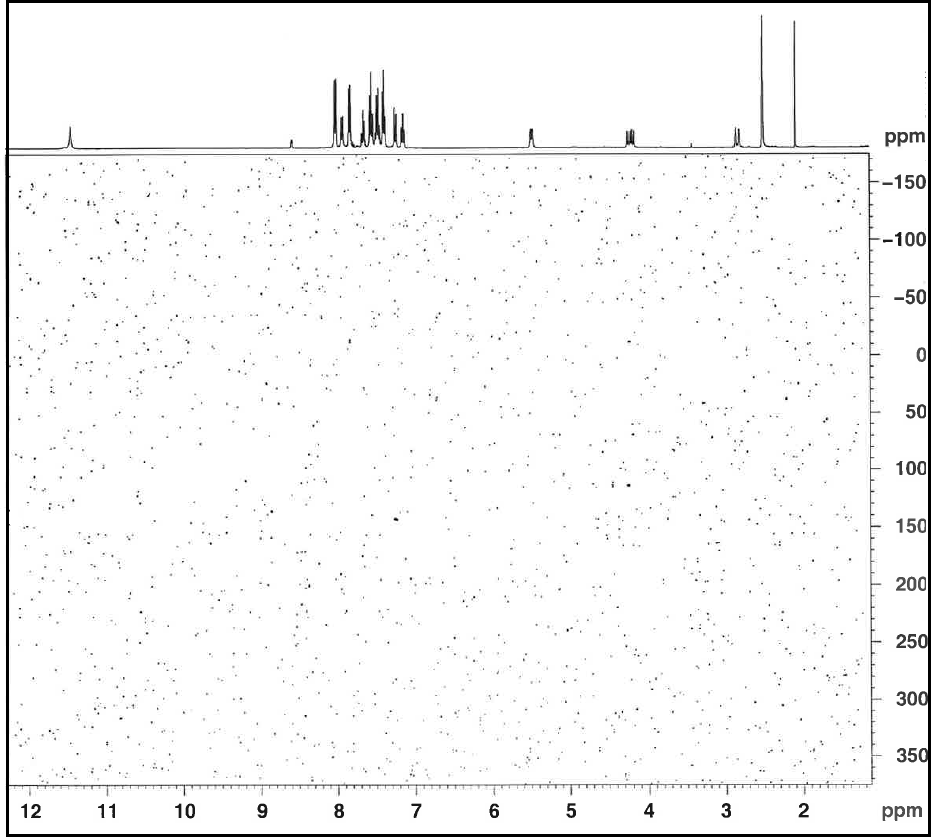
**

**Figure 19. ^1^H-^15^N** HMBC NMR spectrum of compound **6a**

**
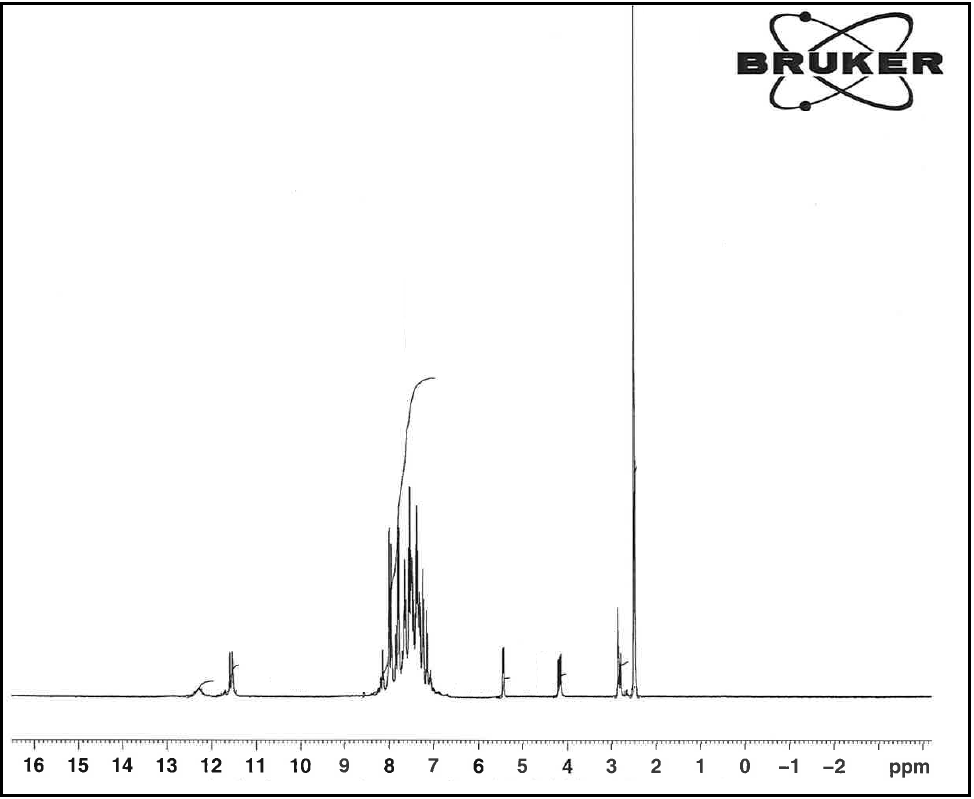
**

**Figure 20. ^1^H** NMR spectrum of compound **6b**

**
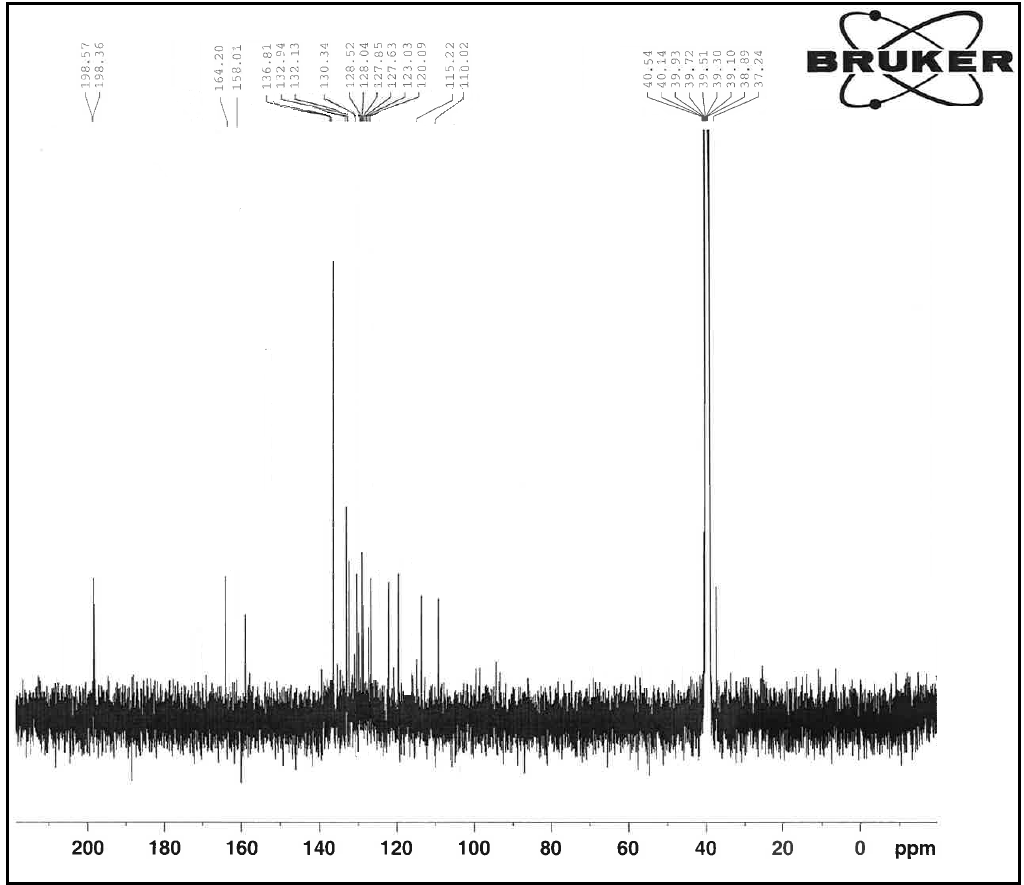
**

**Figure 21. ^13^C** NMR spectrum of compound **6b**

**
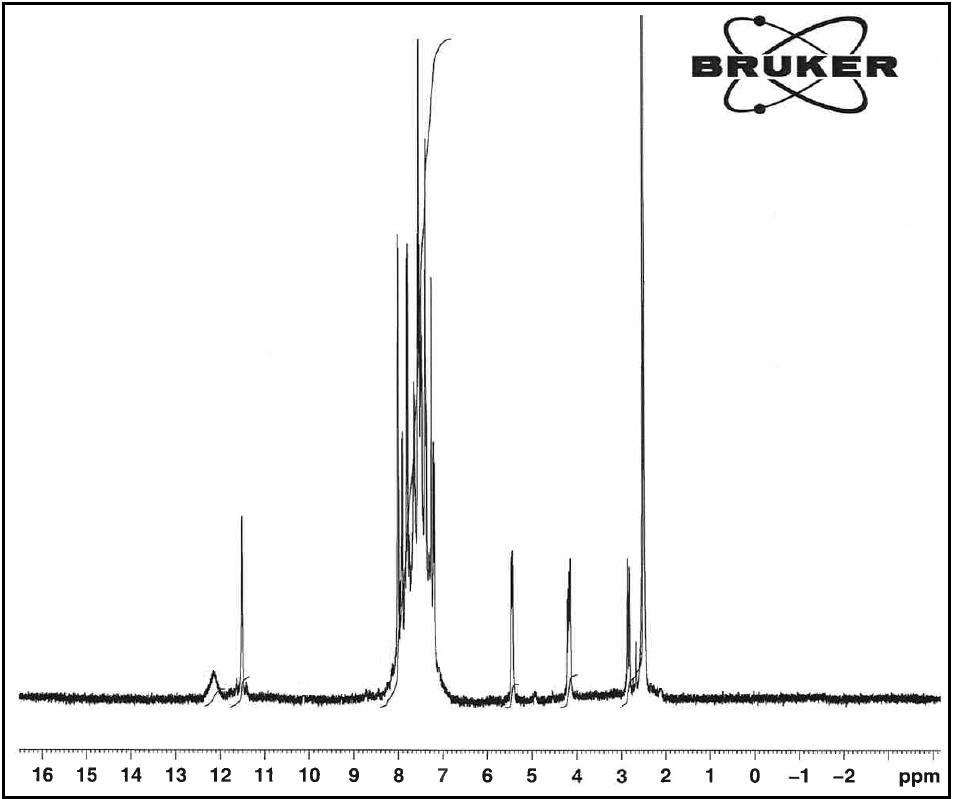
**

**Figure 22. ^1^H** NMR spectrum of compound **6c**

**
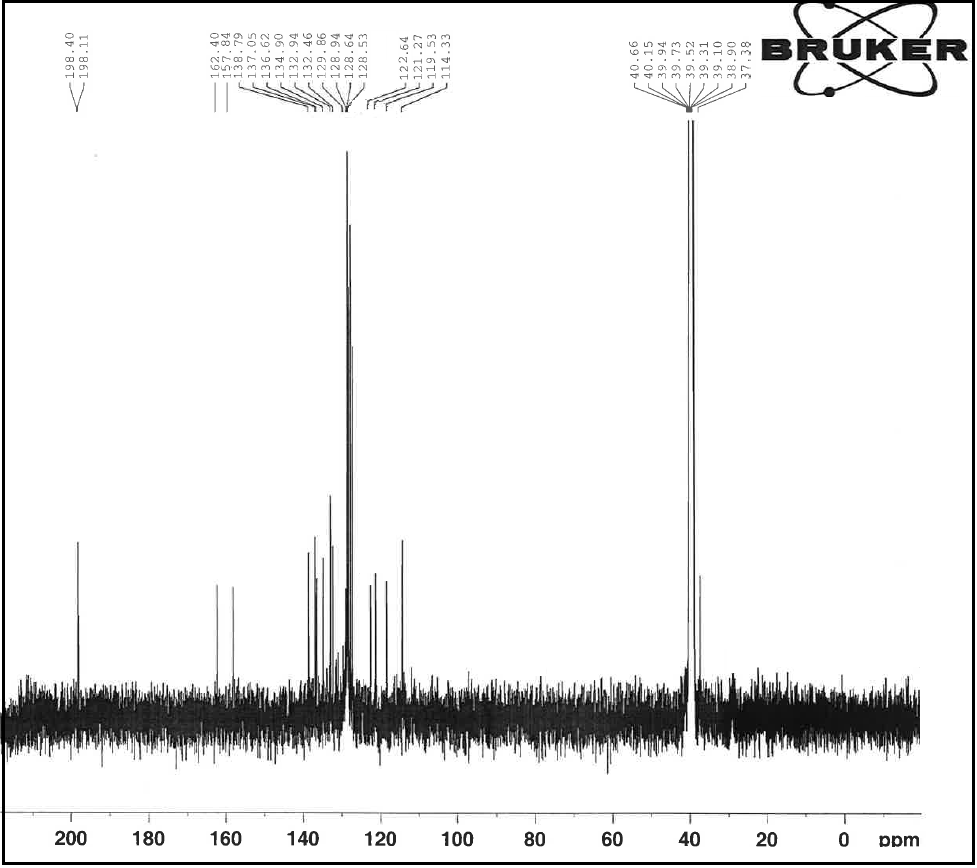
**

**Figure 23. ^13^C** NMR spectrum of compound **6c**

**
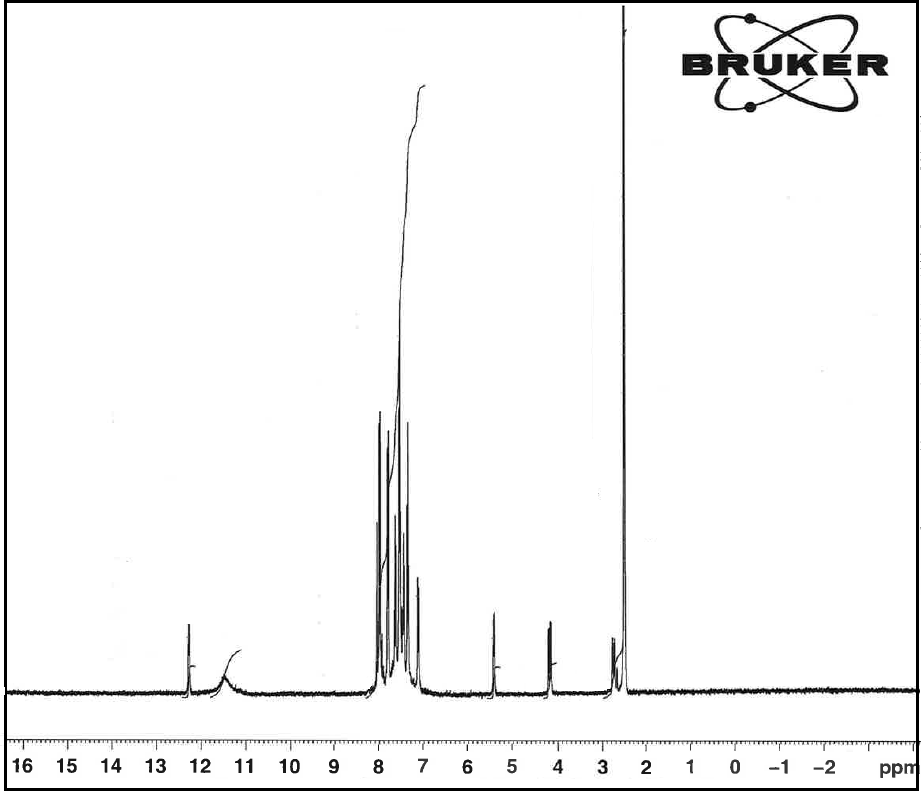
**

**Figure 24. ^1^H** NMR spectrum of compound **6d**

**
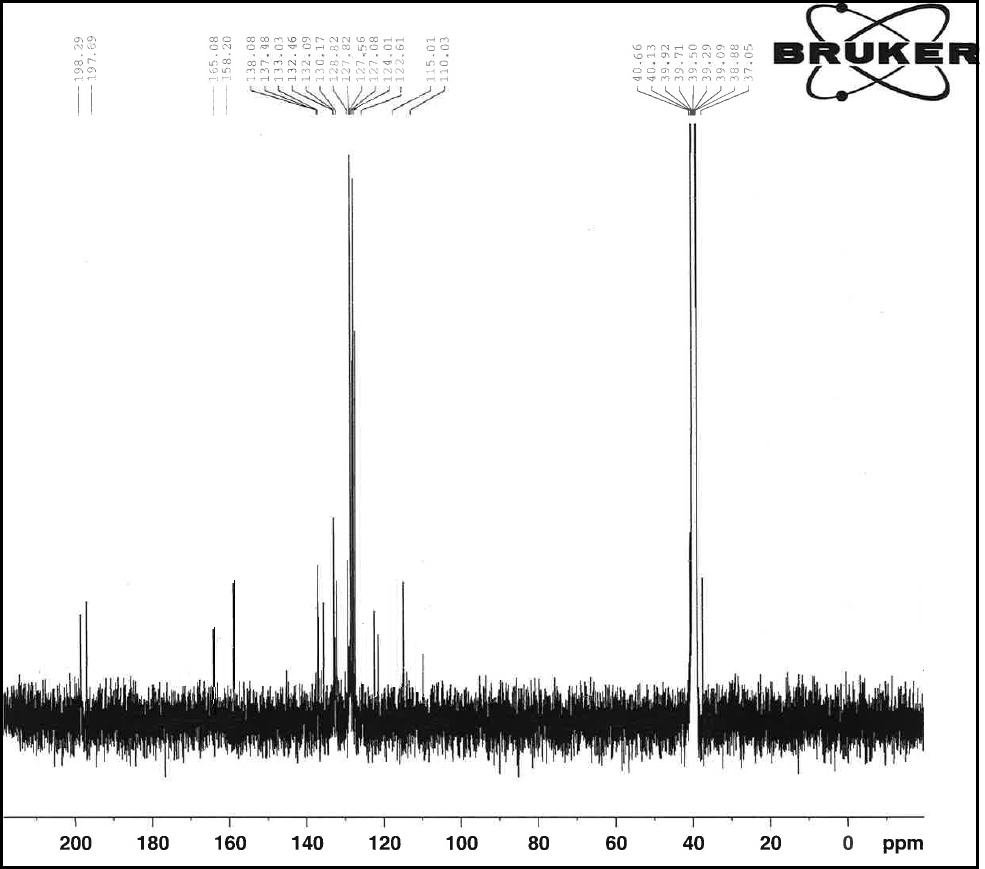
**

**Figure 25. ^13^C** NMR spectrum of compound **6d**

**
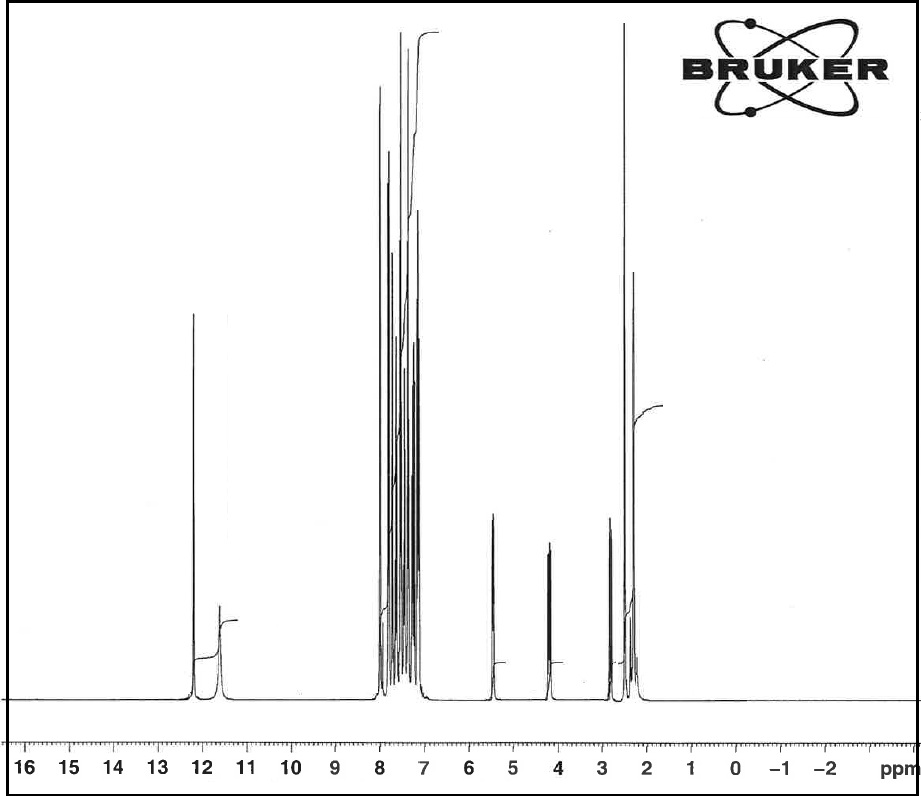
**

**Figure 26. ^1^H** NMR spectrum of compound **6e**

**
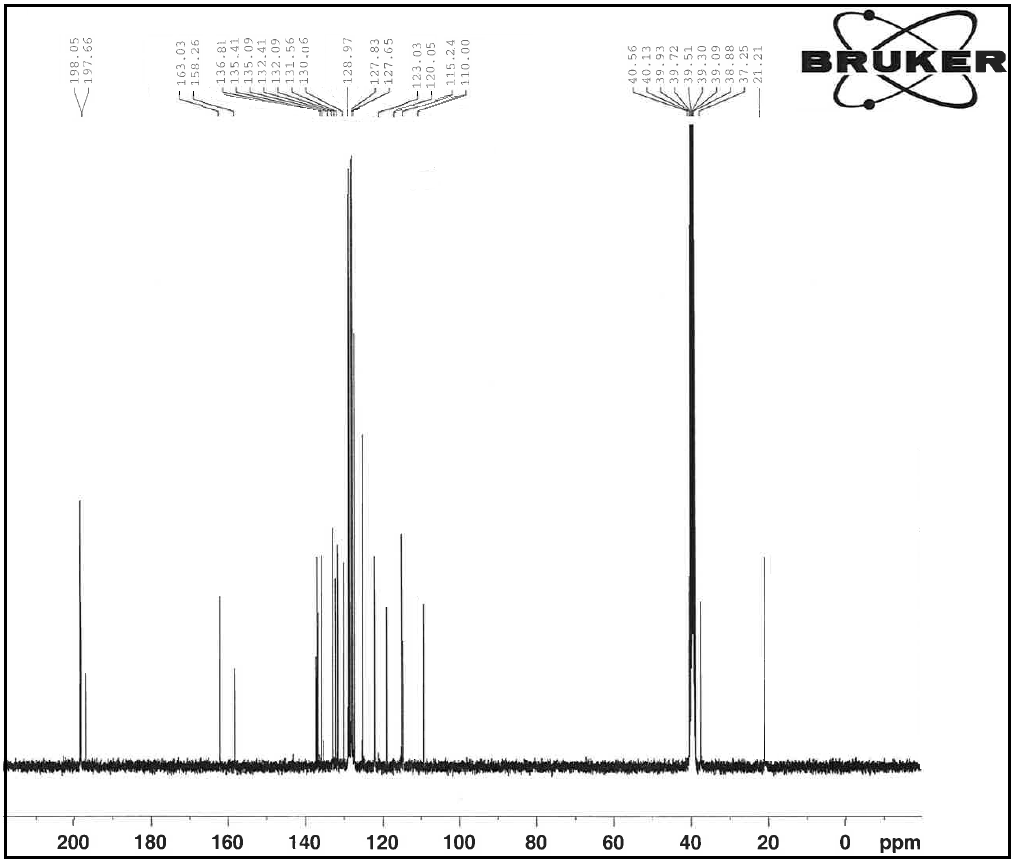
**

**Figure 27. ^13^C** NMR spectrum of compound **6e**

**
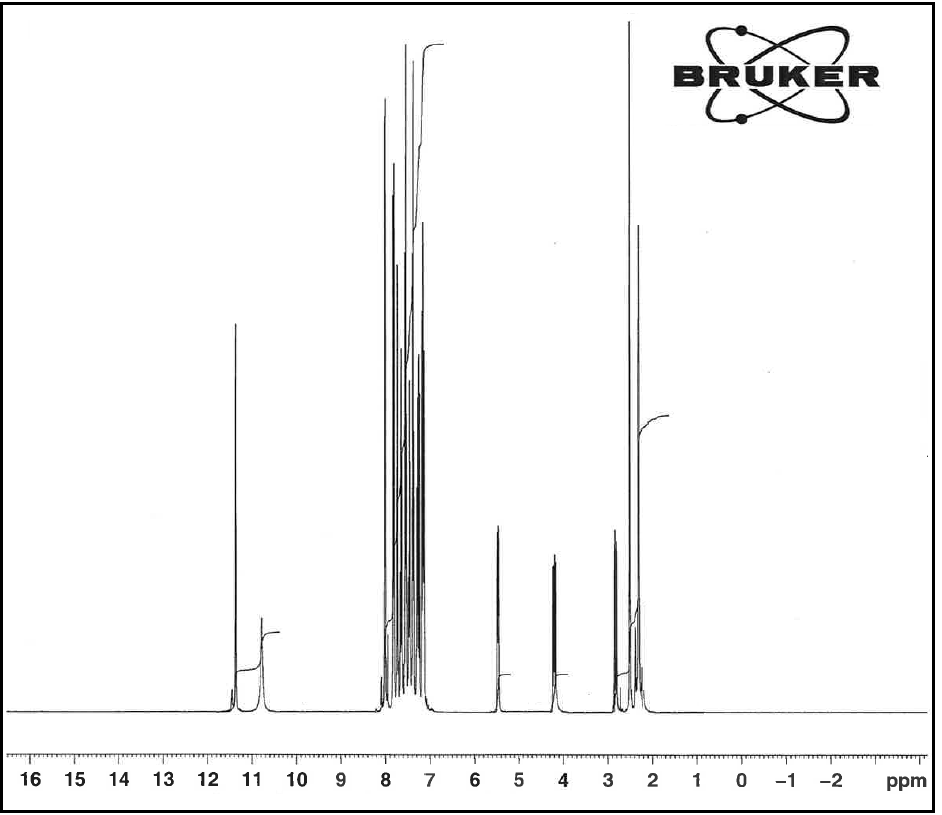
**

**Figure 28. ^1^H** NMR spectrum of compound **6f**

**
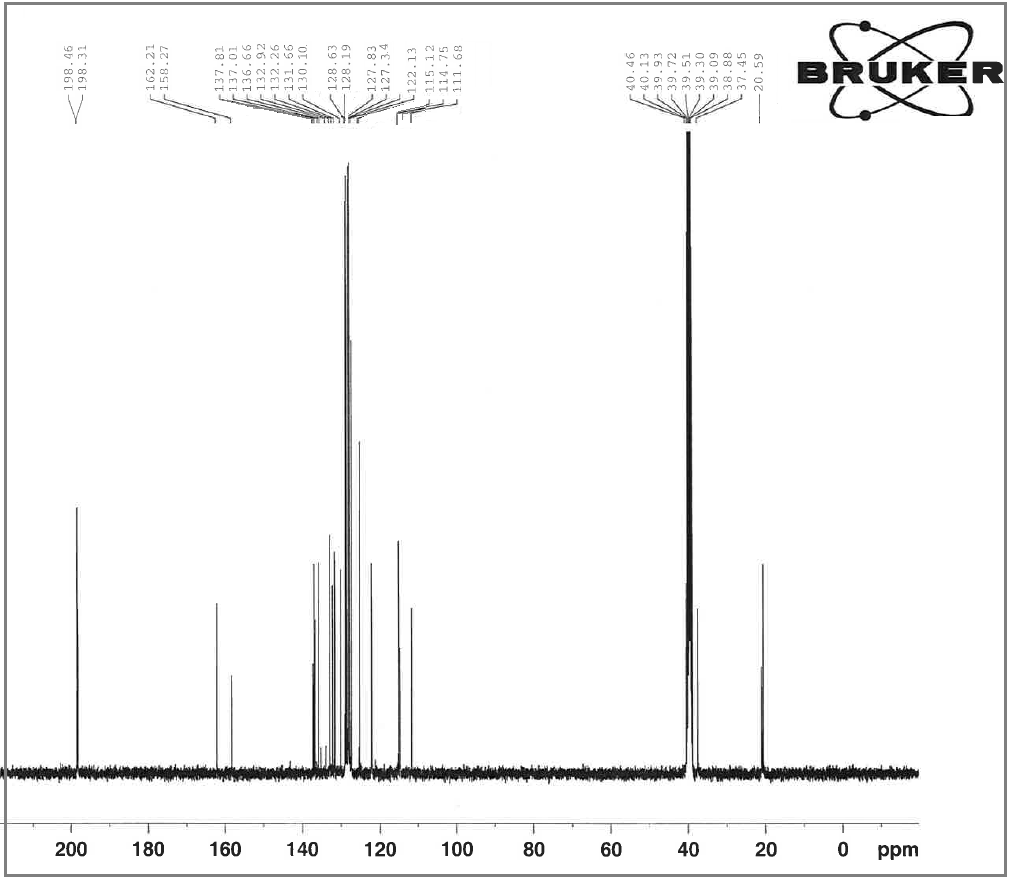
**

**Figure 29. ^13^C** NMR spectrum of compound **6f**

**
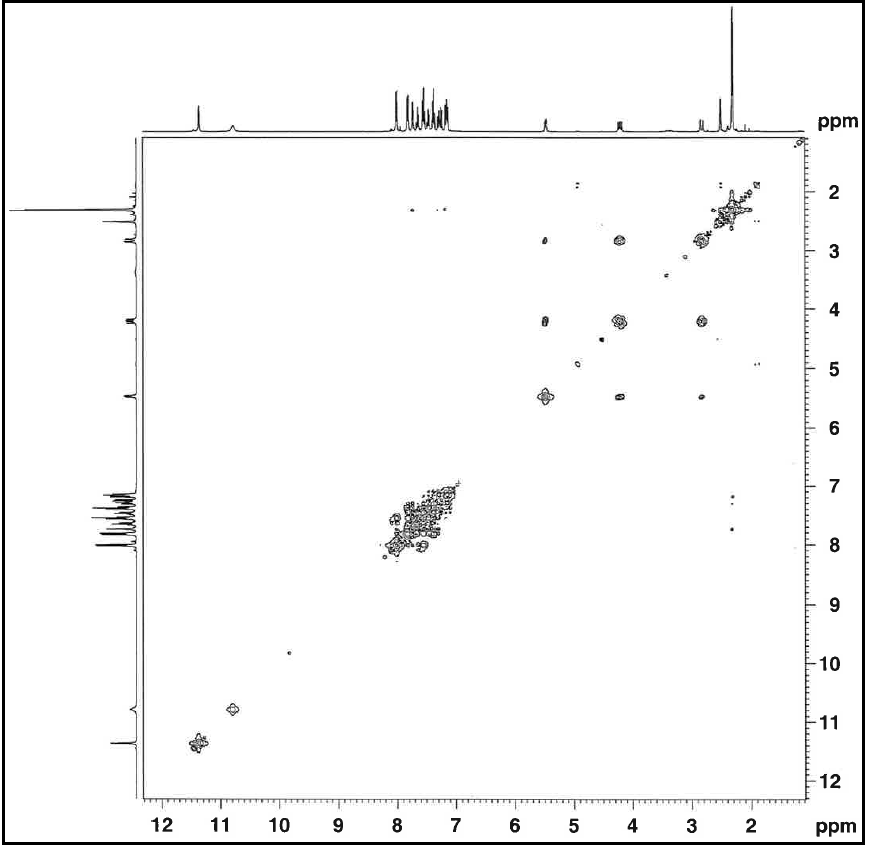
**

**Figure 30. ^1^H-^1^H** COSY NMR spectrum of compound **6f**

**
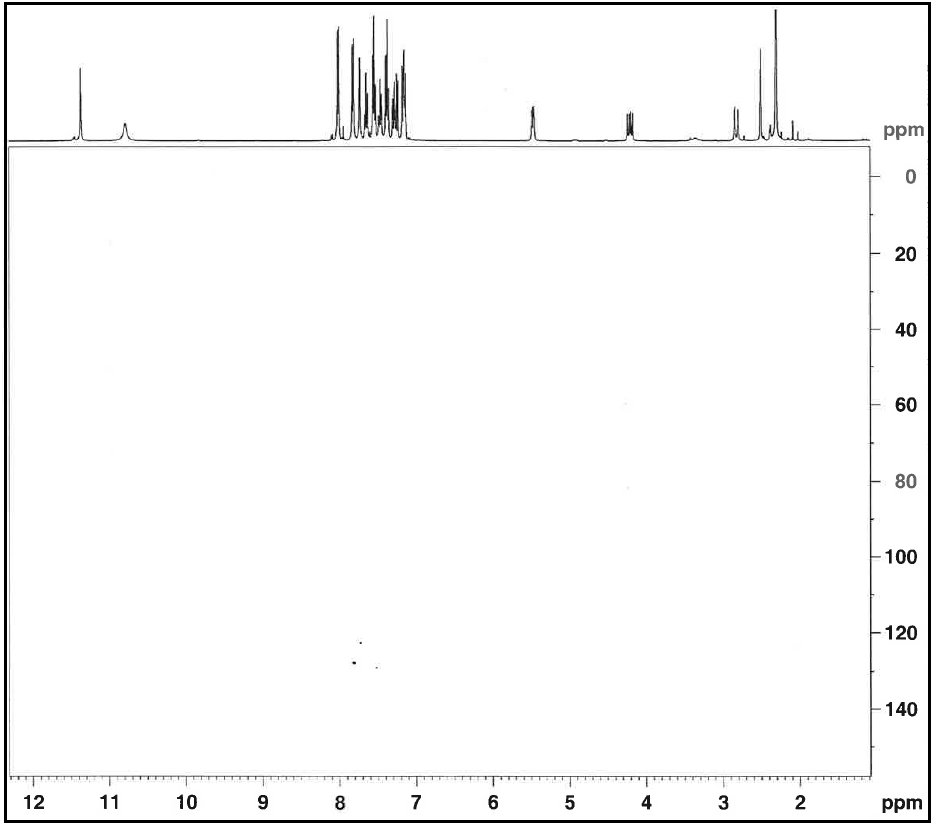
**

**Figure 31. ^1^H-^13^C** HSQC NMR spectrum of compound **6f**

**
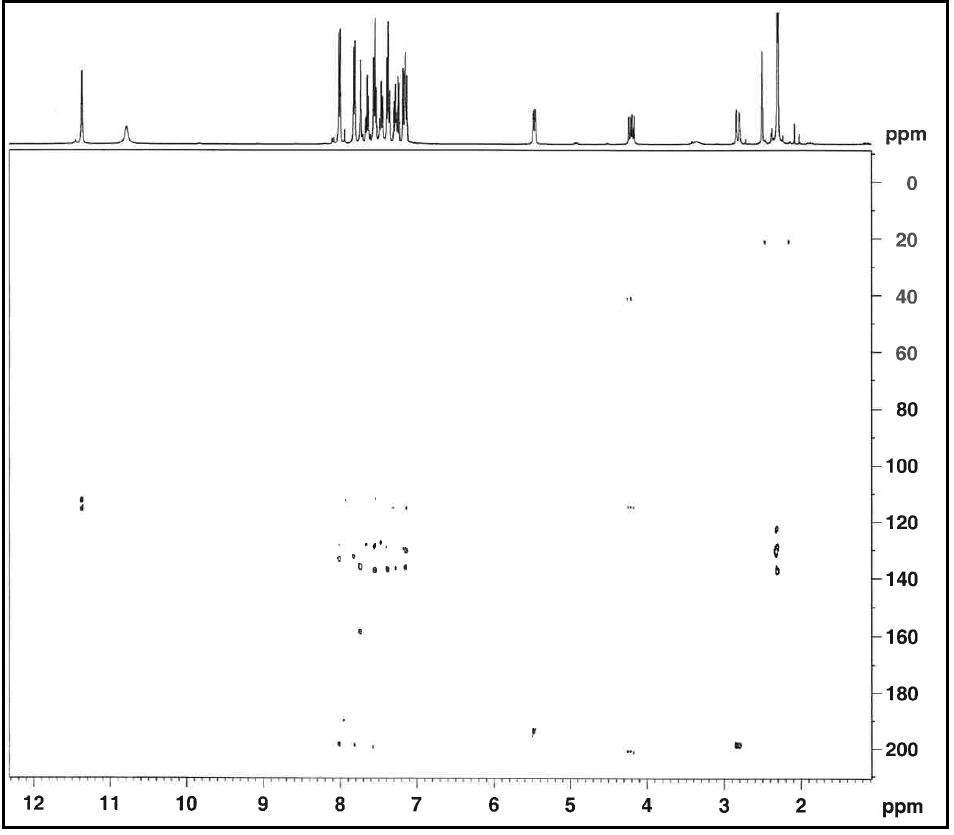
**

**Figure 32. ^1^H-^13^C** HMBC NMR spectrum of compound **6f**

**
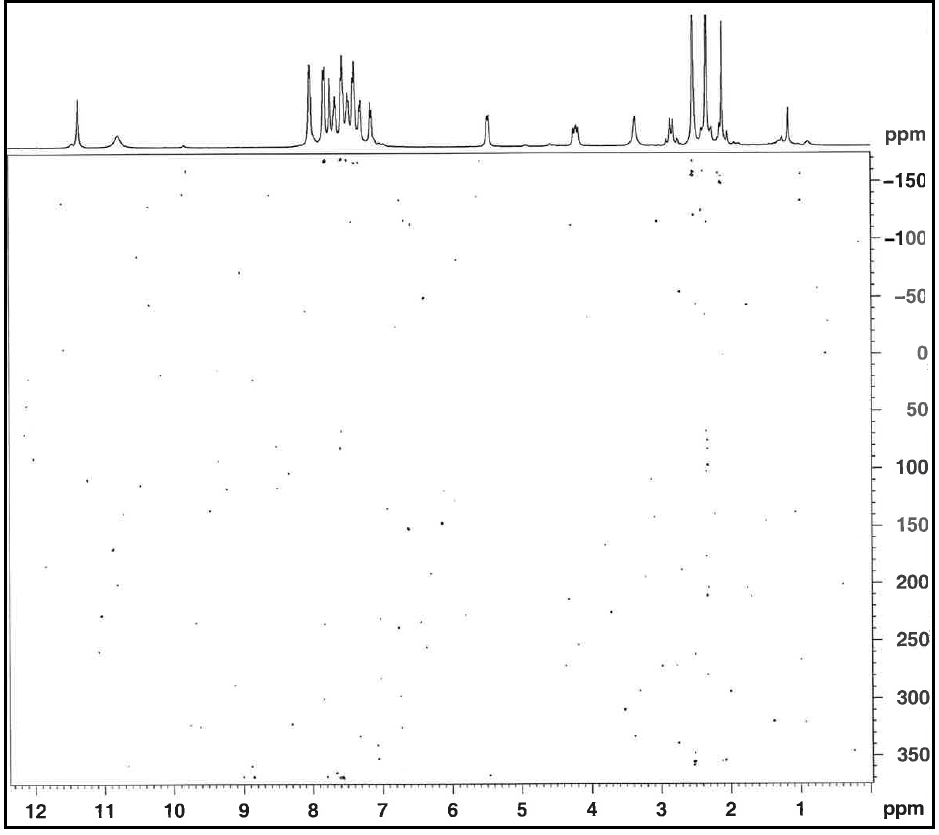
**

**Figure 33. ^1^H-^15^N** HSQC NMR spectrum of compound **6f**

**
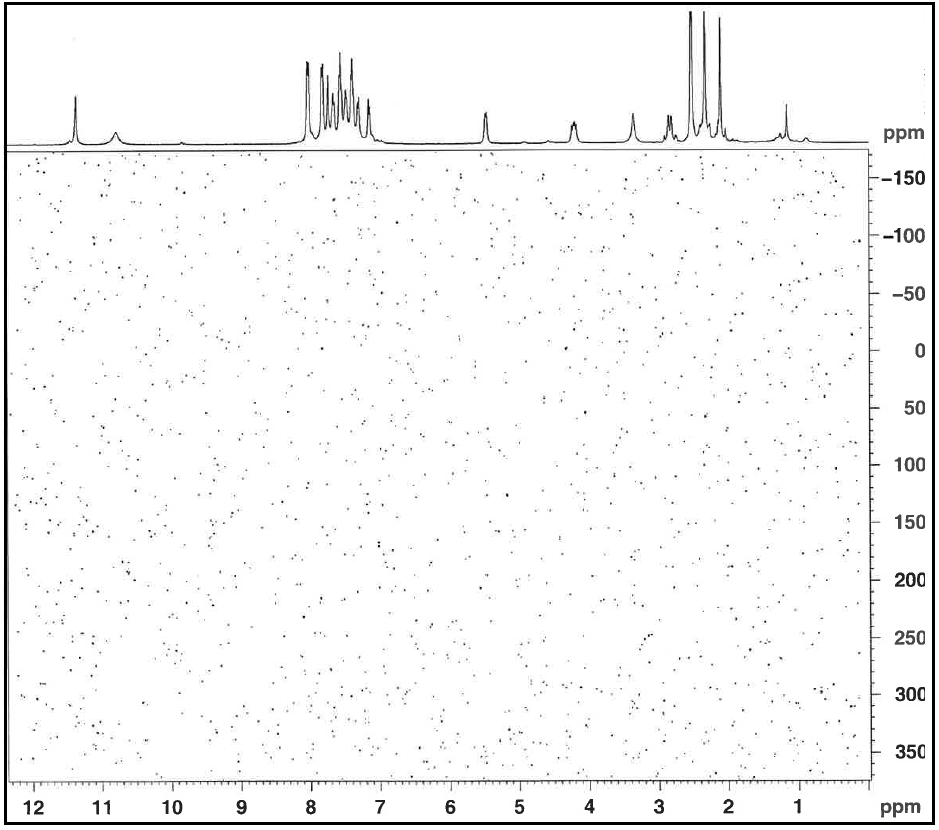
**

**Figure 34. ^1^H-^15^N** HMBC NMR spectrum of compound **6f**

**
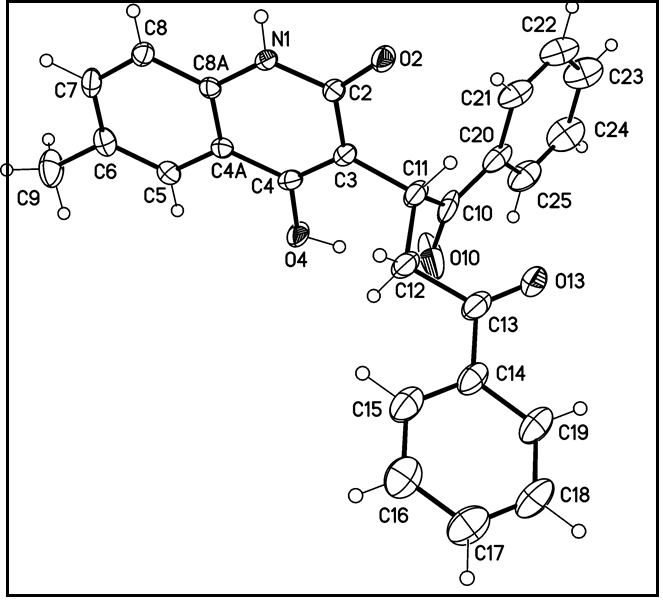
**

**Figure 35. X-ray** structure analysis of compound **6f**

**
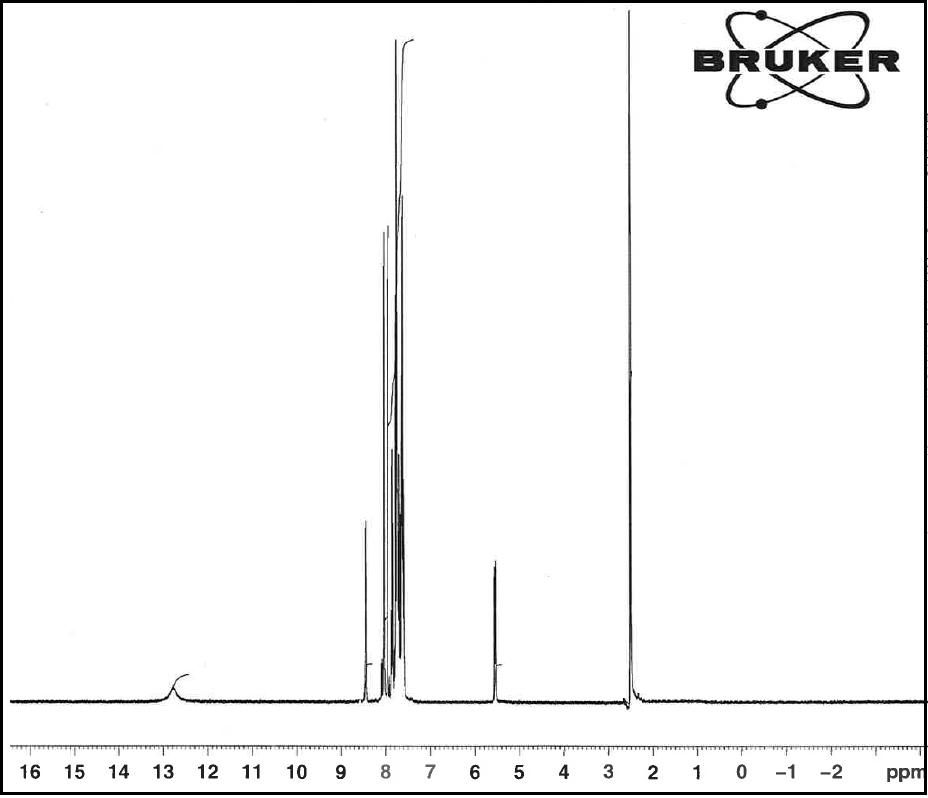
**

**Figure 36. ^1^H** NMR spectrum of compound **11**

**
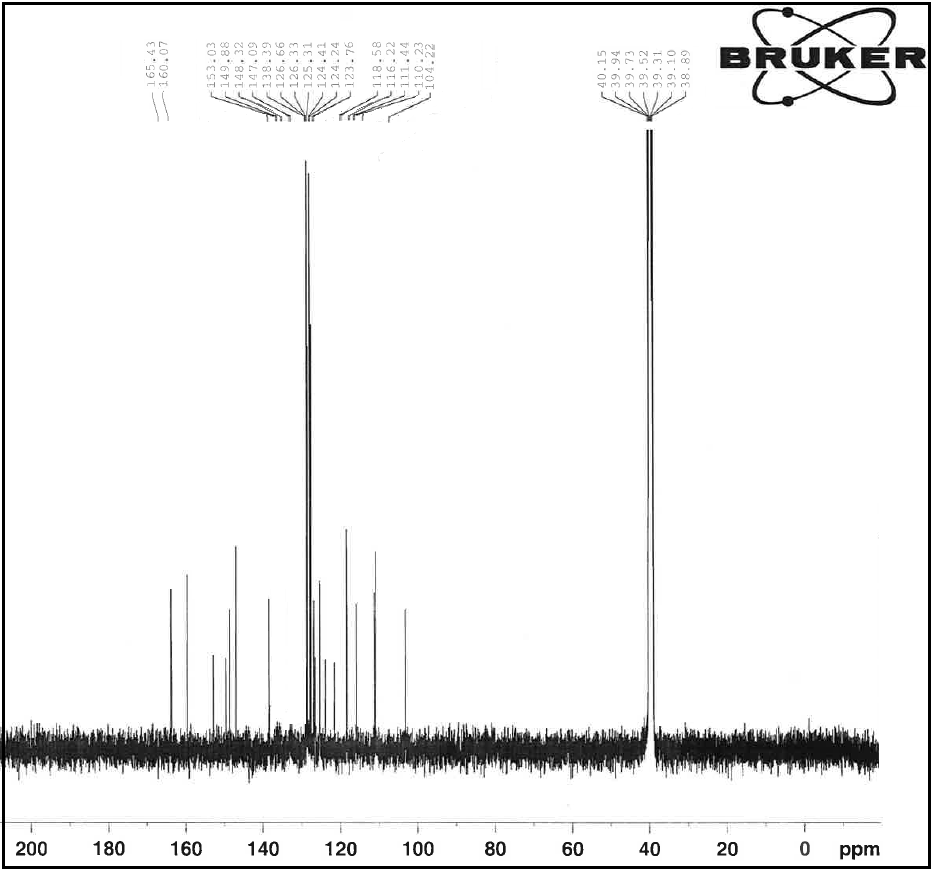
**

**Figure 37. ^13^C** NMR spectrum of compound **11**

**
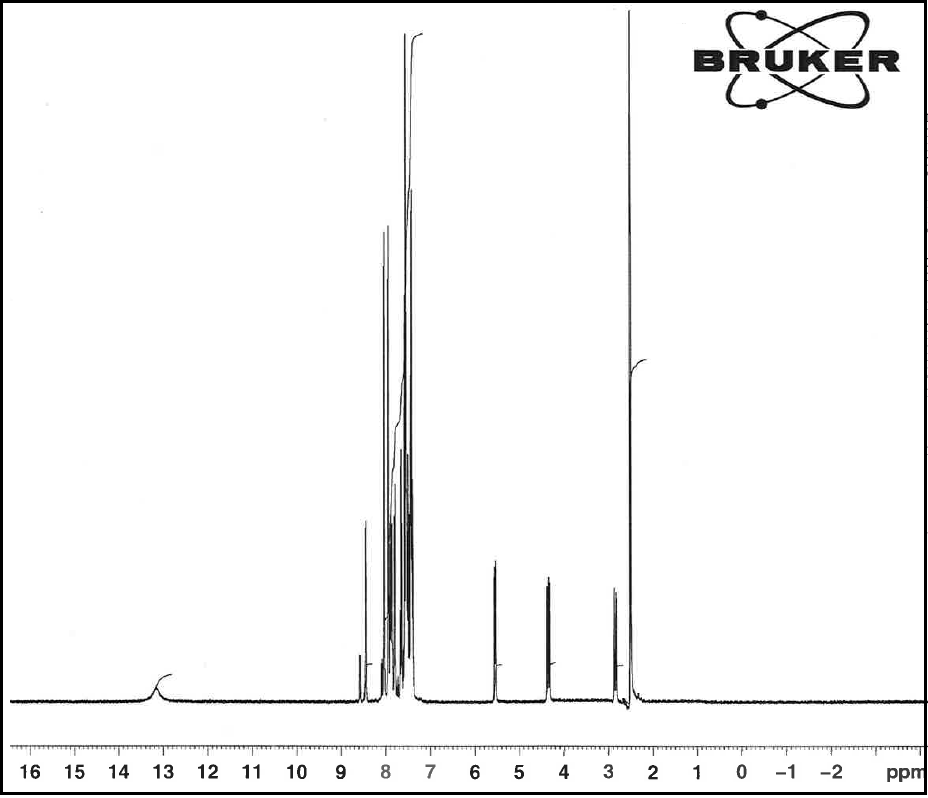
**

**Figure 38. ^1^H** NMR spectrum of compound **12**

**
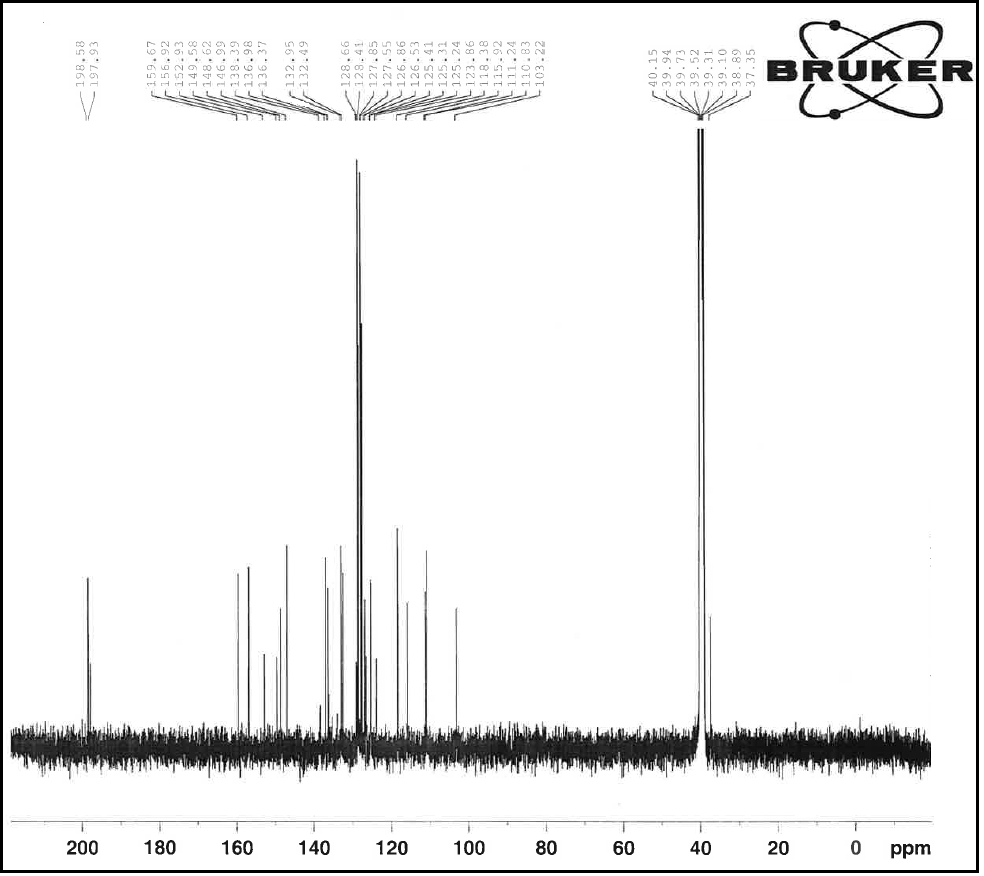
**

**Figure 39. ^13^C** NMR spectrum of compound **12**

**
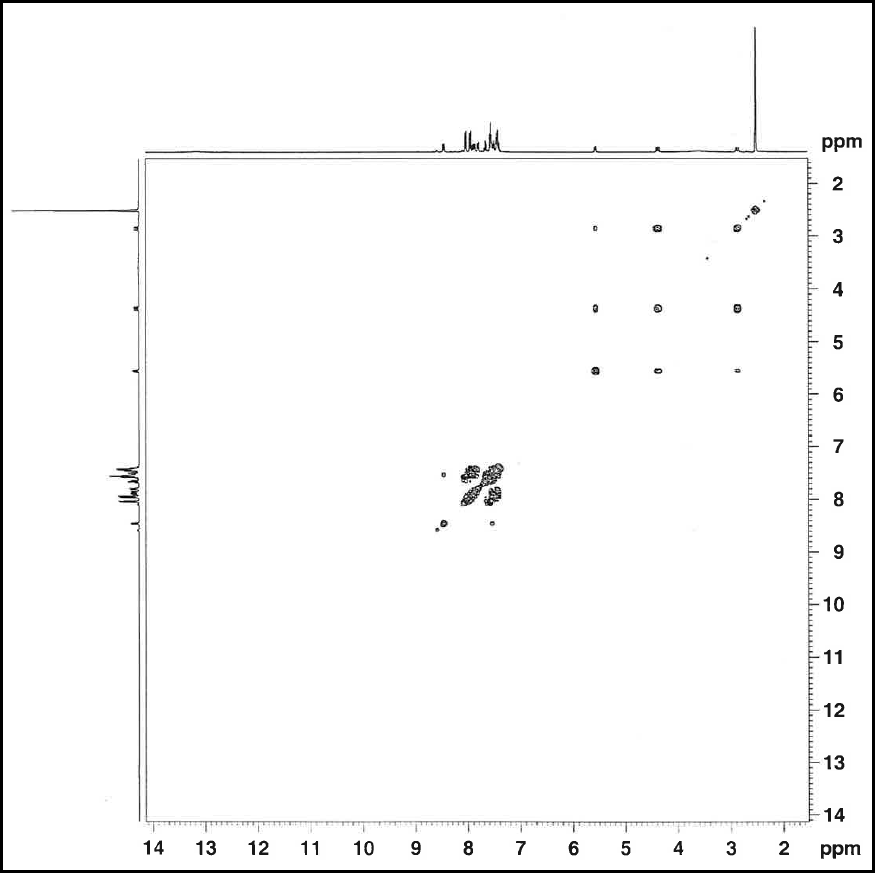
**

**Figure 40. ^1^H-^1^H** COSY NMR spectrum of compound **12**

**
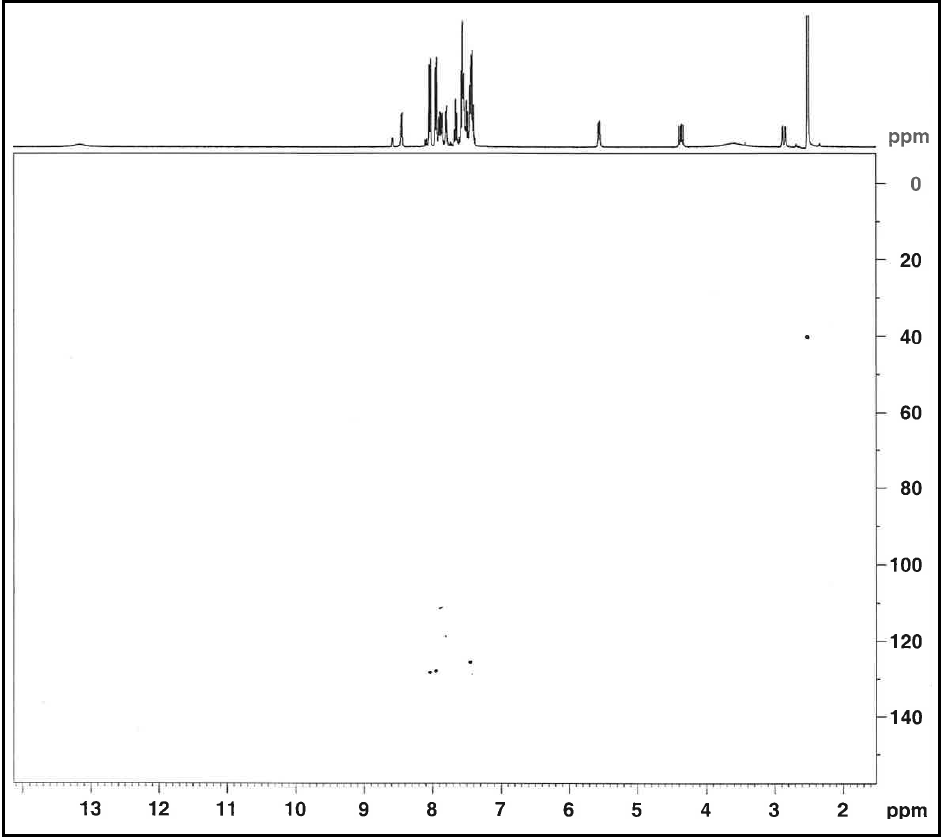
**

**Figure 41. ^1^H-^13^C** HSQC NMR spectrum of compound **12**

**
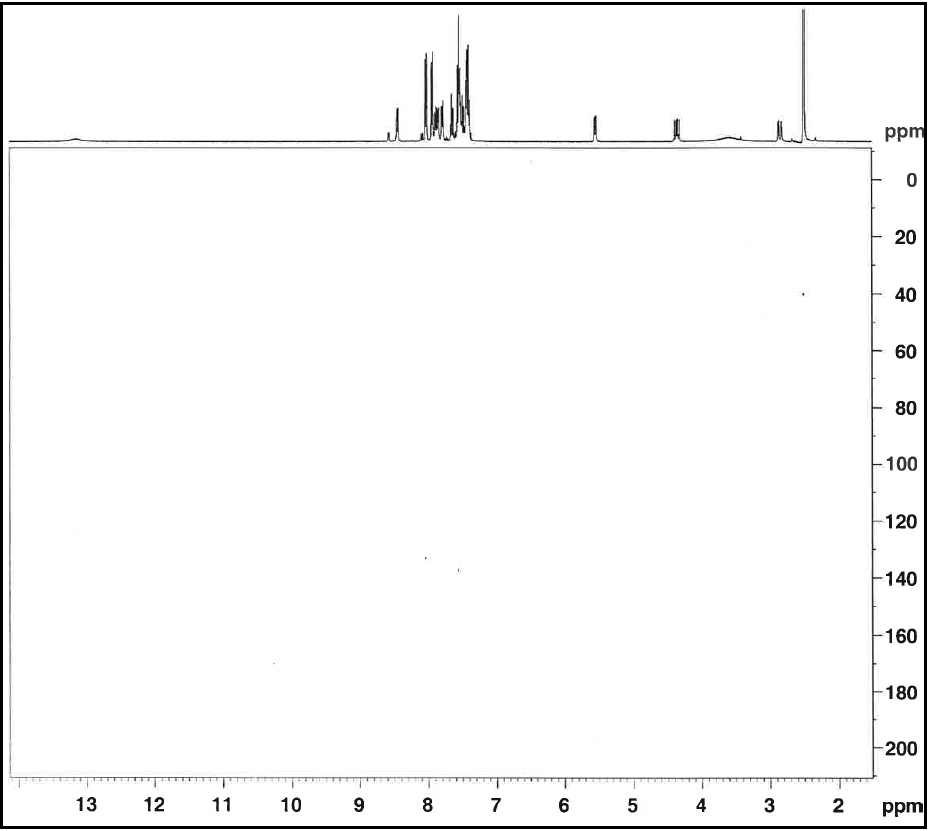
**

**Figure 42. ^1^H-^13^C** HMBC NMR spectrum of compound **12**

**
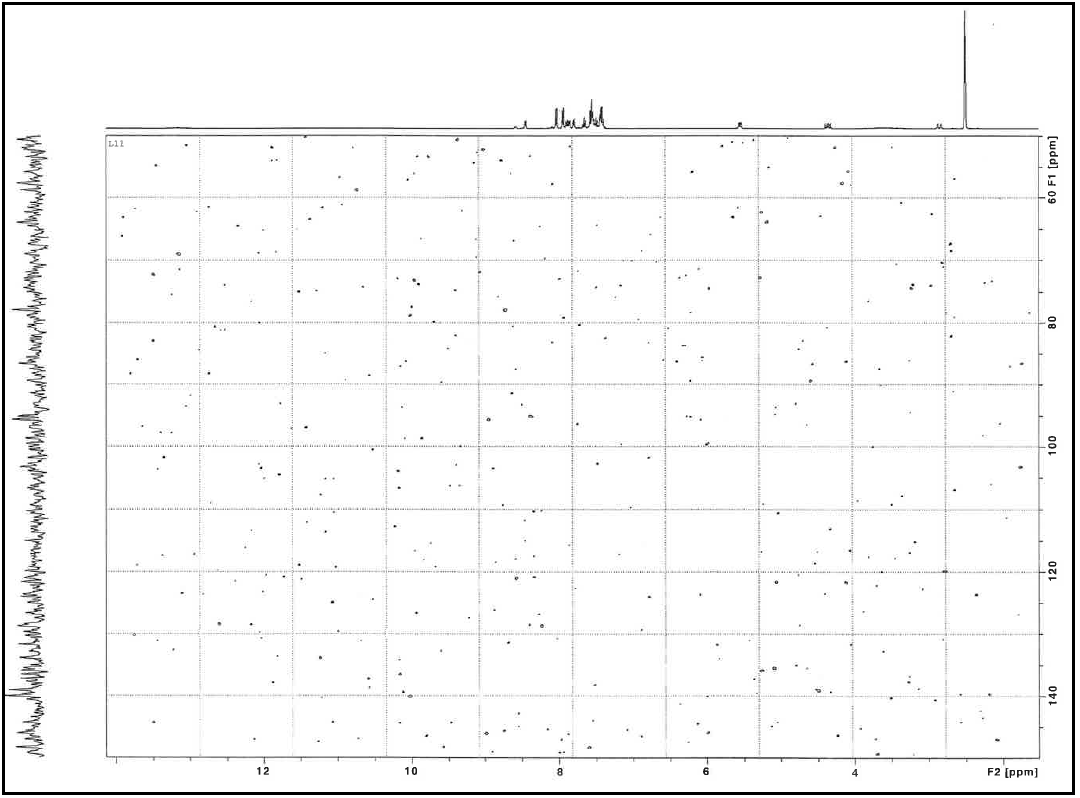
**

**Figure 43. ^1^H-^15^N** HSQC NMR spectrum of compound **12**

**
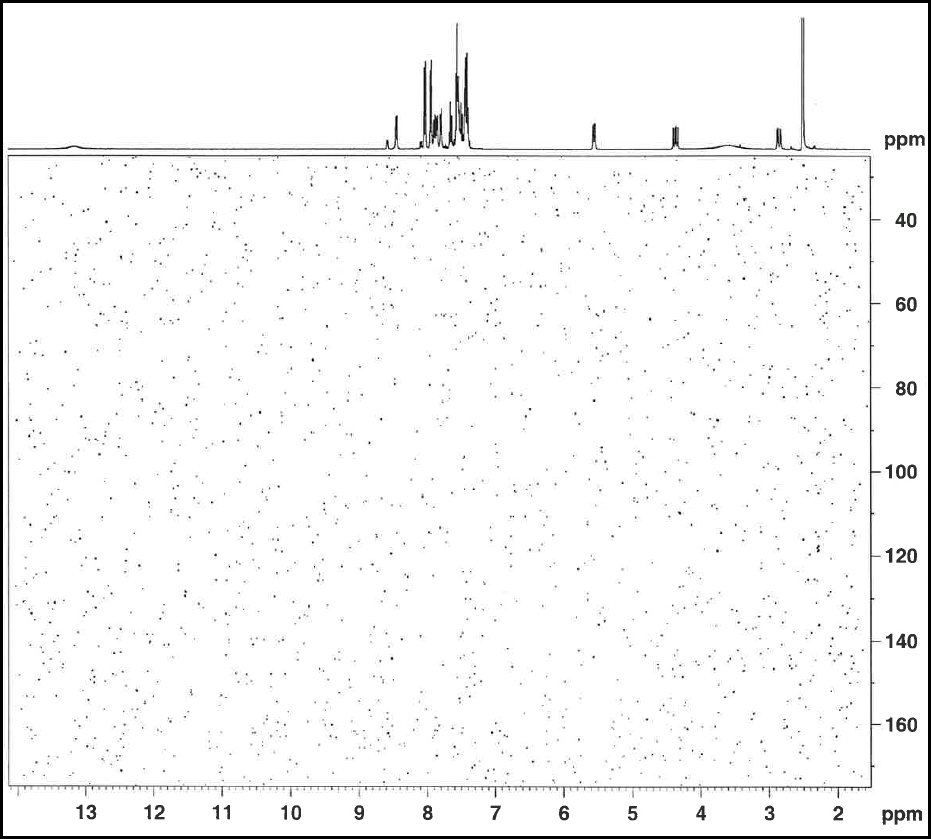
**

**Figure 45. ^1^H-^15^N** HMBC NMR spectrum of compound **12**

**
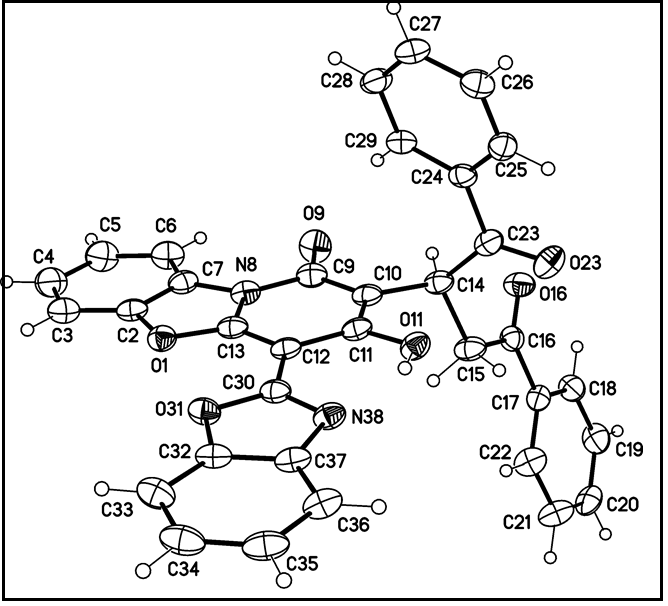
**

**Figure 46. X-ray** structure analysis of compound **12**
