# Supplementary material for: Synthesis of New Fused Heterocyclic 2-Quinolones and 3-Alkanonyl-4-Hydroxy-2-Quinolones
Source: Molecules. 2019 Oct 21;24(20):3782. doi: 10.3390/molecules24203782 (PMC6832483; doi:10.3390/molecules24203782)
Supplement: Supplementary file 1 [file molecules-24-03782-s001.zip › Molecules_Aly_Quinolones_SI1_X-ray file.docx]

Crystal Structure Determinations

The single-crystal X-ray diffraction study were carried out on a Bruker D8 Venture diffractometer with Photon100 detector at 123(2) K using Cu-Kα radiation (*λ* = 1.54178 Å. Direct methods (SHELXS-97 for **6a**) [G. M. Sheldrick, *Acta Crystallogr.* 2008, **A64**, 112-122; doi.org/10.1107/S0108767307043930] or dual space / intrinsic methods (SHELXT for **12**) [G. M. Sheldrick, *Acta Crystallogr.* 2015, **A71**, 3-8; doi.org/10.1107/S2053273314026370] were used for structure solution and refinement was carried out using SHELXL-2014 (full-matrix least-squares on *F^2^*) [G. M. Sheldrick, *Acta Crystallogr.* 2015, **C71**, 3-8; doi.org/10.1107/S2053229614024218]. Hydrogen atoms were localized by difference electron density determination and refined using a riding model (H(N, O) free). Semi-empirical absorption corrections were applied.

**6a**: colourless crystals, C_26_H_21_NO_4_, *M*_r_ = 411.44, crystal size 0.16 × 0.08 × 0.06 mm, triclinic, space group *P-1* (No. 2), *a* = 11.1823(3) Å, *b* = 14.3827(4) Å, *c* = 15.3460(4) Å, *α* = 67.695(1)°, *β* = 70.666(1)°, *γ* = 68.389(1)°, *V* = 2069.77(10) Å^3^, *Z* = 4, *ρ* = 1.320 Mg/m^-3^, *µ*(Cu-K_α_) = 0.72 mm^-1^, *F*(000) = 864, *2θ*_max_ = 144.2°, 28579 reflections, of which 8117 were independent (*R*_int_ = 0.035), 577 parameters, 5 restraints, *R*_1_ = 0.043 (for 6749 I > 2σ(I)), w*R*_2_ = 0.112 (all data), *S* = 1.01, largest diff. peak / hole = 0.37 / -0.50 e Å^-3^.

**12**: colourless crystals, C_34_H_2_N_2_O_6_, *M*_r_ = 554.53, crystal size 0.22 × 0.09 × 0.03 mm, triclinic, space group *P-1* (No. 2), *a* = 8.2672(2) Å, *b* = 11.3140(3) Å, *c* = 13.7326(3) Å, *α* = 91.721(1)°, *β* = 96.833(1)°, *γ* = 92.198(1)°, *V* = 1273.64(5) Å^3^, *Z* = 2, *ρ* = 1.446 Mg/m^-3^, *µ*(Cu-K_α_) = 0.82 mm^-1^, *F*(000) = 576, *2θ*_max_ = 144.4°, 18981 reflections, of which 4987 were independent (*R*_int_ = 0.037), 382 parameters, 1 restraint, *R*_1_ = 0.048 (for 4212 I > 2σ(I)), w*R*_2_ = 0.127 (all data), *S* = 1.03, largest diff. peak / hole = 0.35 / -0.25 e Å^-3^.

CCDC 1913026 (**6a**), and 1913027 (**12**) contain the supplementary crystallographic data for this paper. These data can be obtained free of charge from The Cambridge Crystallographic Data Centre via [www.ccdc.cam.ac.uk/data_request/cif](http://www.ccdc.cam.ac.uk/data_request/cif).

a) SHELXS: G. M. Sheldrick, *Acta Crystallogr.* 2008, **A64**, 112-122; doi.org/10.1107/S0108767307043930.

b) SHELXT; G. M. Sheldrick, *Acta Crystallogr.* 2015, **A71**, 3-8; doi.org/10.1107/S2053273314026370.

c) SHELXL-2014; G. M. Sheldrick, *Acta Crystallogr.* 2015, **C71**, 3-8; doi.org/10.1107/S2053229614024218.





Fig. 1x. Molecular structure of **6a** (displacement parameters are drawn at 50 % probability level, both crystallographic independent molecules shown).





Fig. 2x. Molecular structure of **12** (displacement parameters are drawn at 50 % probability level).
