# Supplementary material for: Synthesis of New Fused Heterocyclic 2-Quinolones and 3-Alkanonyl-4-Hydroxy-2-Quinolones
Source: Molecules. 2019 Oct 21;24(20):3782. doi: 10.3390/molecules24203782 (PMC6832483; doi:10.3390/molecules24203782)
Supplement: Supplementary file 1 [file molecules-24-03782-s001.zip › Molecules_Aly_Quinolones_SI1_X-ray of SB993.docx]

**
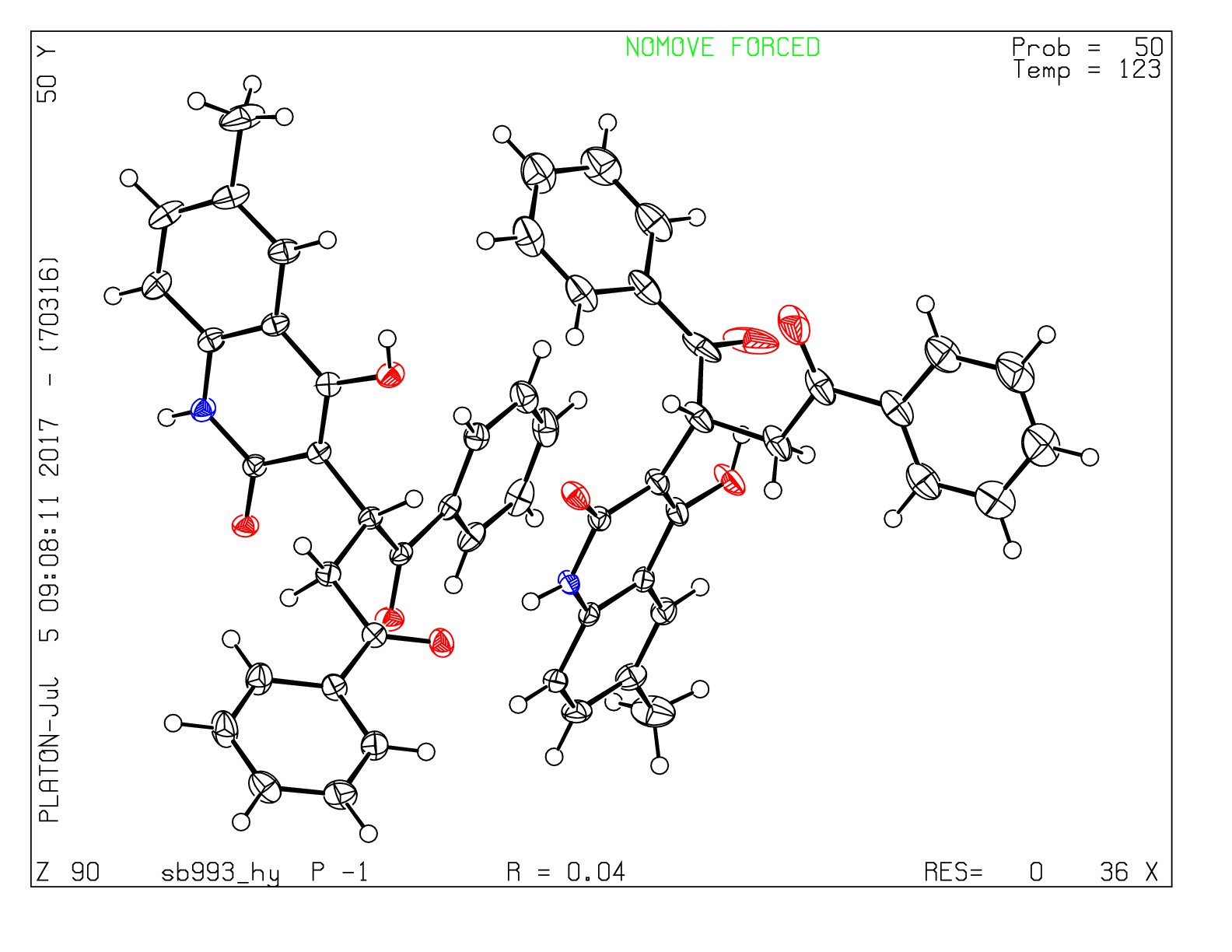
SB993_HY**

**L3 (L4 identical)**


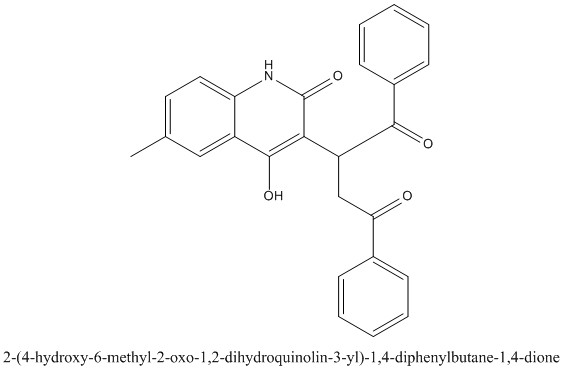


**2-(4-hydroxy-6-methyl-2-oxo-1,2-dihydroquinolin-3-yl)-1,4-diphenylbutane-1,4-dione – SB993_HY**

***Crystal data***

| **C26H21NO4** | ***Z* = 4** |
| --- | --- |
| ***Mr* = 411.44** | ***F*(000) = 864** |
| **Triclinic, *P*-1 (no.2)** | ***D*x = 1.320 Mg m-3** |
| ***a* = 11.1823 (3) Å** | **Cu *K*α radiation,  = 1.54178 Å** |
| ***b* = 14.3827 (4) Å** | **Cell parameters from 9863 reflections** |
| ***c* = 15.3460 (4) Å** | ** = 3.1–72.1°** |
| **α = 67.695 (1)°** | ** = 0.72 mm-1** |
| **β = 70.666 (1)°** | ***T* = 123 K** |
| **γ = 68.389 (1)°** | **Blocks, colourless** |
| ***V* = 2069.77 (10) Å3** | **0.16 × 0.08 × 0.06 mm** |

***Data collection***

| **Bruker D8 VENTURE diffractometer with Photon100 detector** | **8117 independent reflections** |
| --- | --- |
| **Radiation source: INCOATEC microfocus sealed tube** | **6749 reflections with *I* > 2σ(*I*)** |
| **Detector resolution: 10.4167 pixels mm-1** | ***R*int = 0.035** |
| **rotation in  and , 1°, shutterless scans** | **max = 72.1°, min = 3.2°** |
| **Absorption correction: multi-scan  *SADABS* (Sheldrick, 2014)** | ***h* = -1313** |
| ***T*min = 0.896, *T*max = 0.958** | ***k* = -1717** |
| **28579 measured reflections** | ***l* = -1818** |

***Refinement***

| **Refinement on *F*2** | **Primary atom site location: structure-invariant direct methods** |
| --- | --- |
| **Least-squares matrix: full** | **Secondary atom site location: difference Fourier map** |
| ***R*[*F*2 > 2σ(*F*2)] = 0.043** | **Hydrogen site location: difference Fourier map** |
| ***wR*(*F*2) = 0.112** | **H atoms treated by a mixture of independent and constrained refinement** |
| ***S* = 1.01** | ***w* = 1/[2(*F*o2) + (0.0508*P*)2 + 1.0588*P*]  where *P* = (*F*o2 + 2*F*c2)/3** |
| **8117 reflections** | **(Δ/σ)max < 0.001** |
| **577 parameters** | **max = 0.37 e Å-3** |
| **5 restraints** | **min = -0.50 e Å-3** |














**L.S. fit of the two crystallographic independent molecules (dihydroquinolin-ring fitted)**





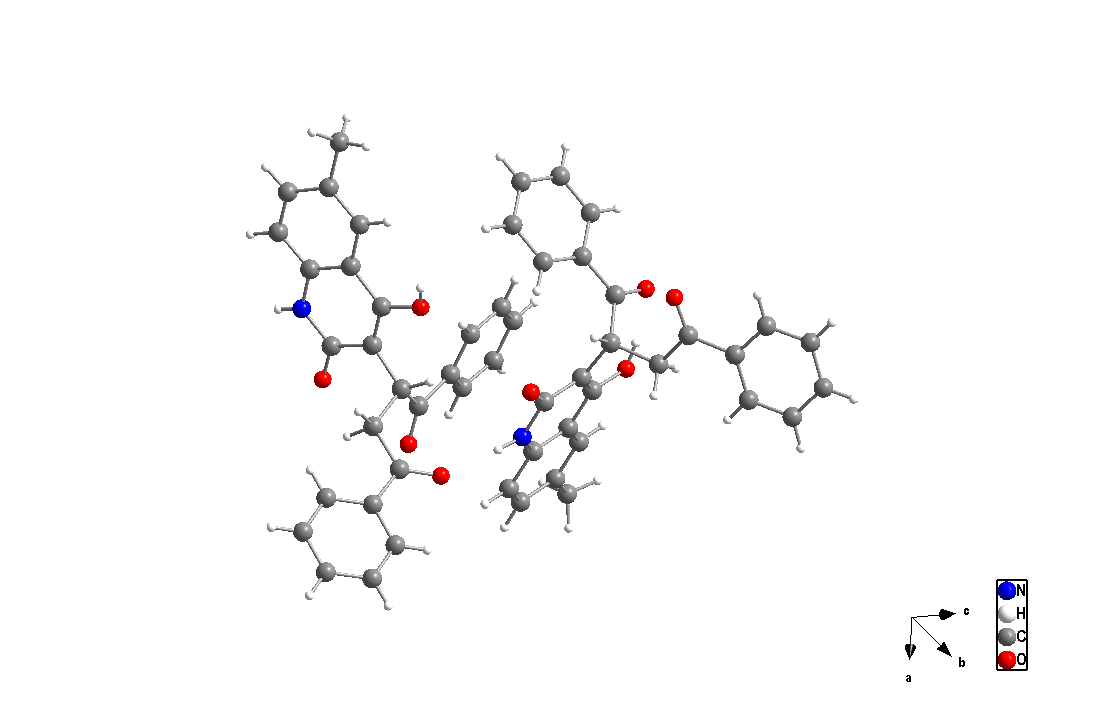


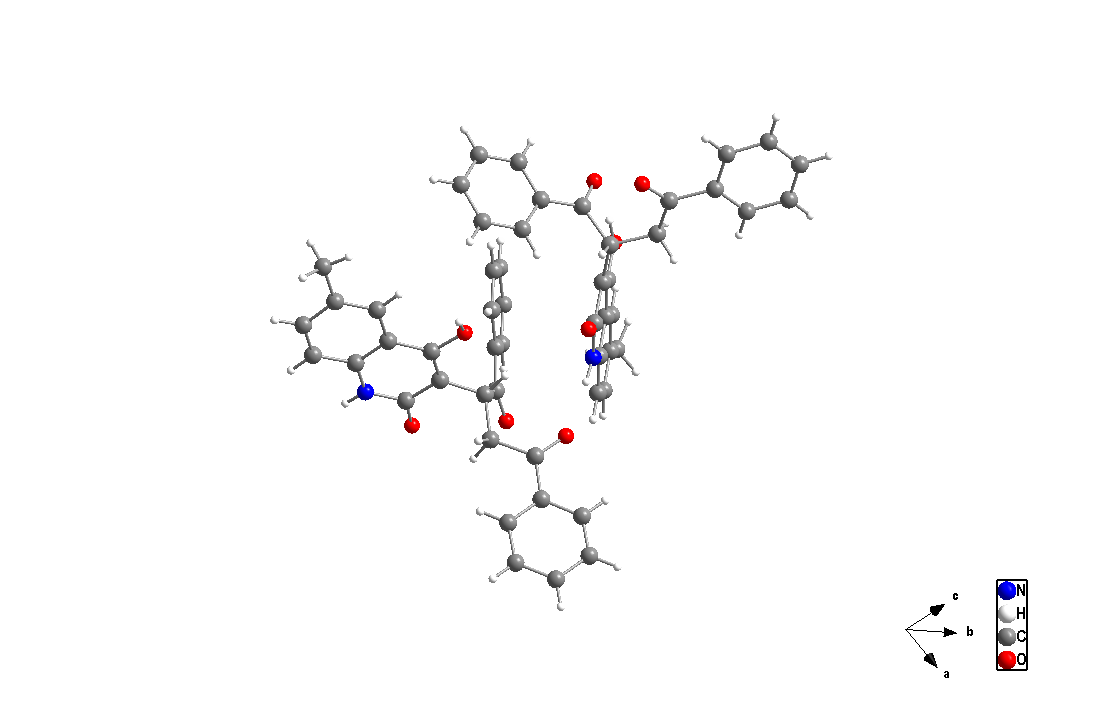


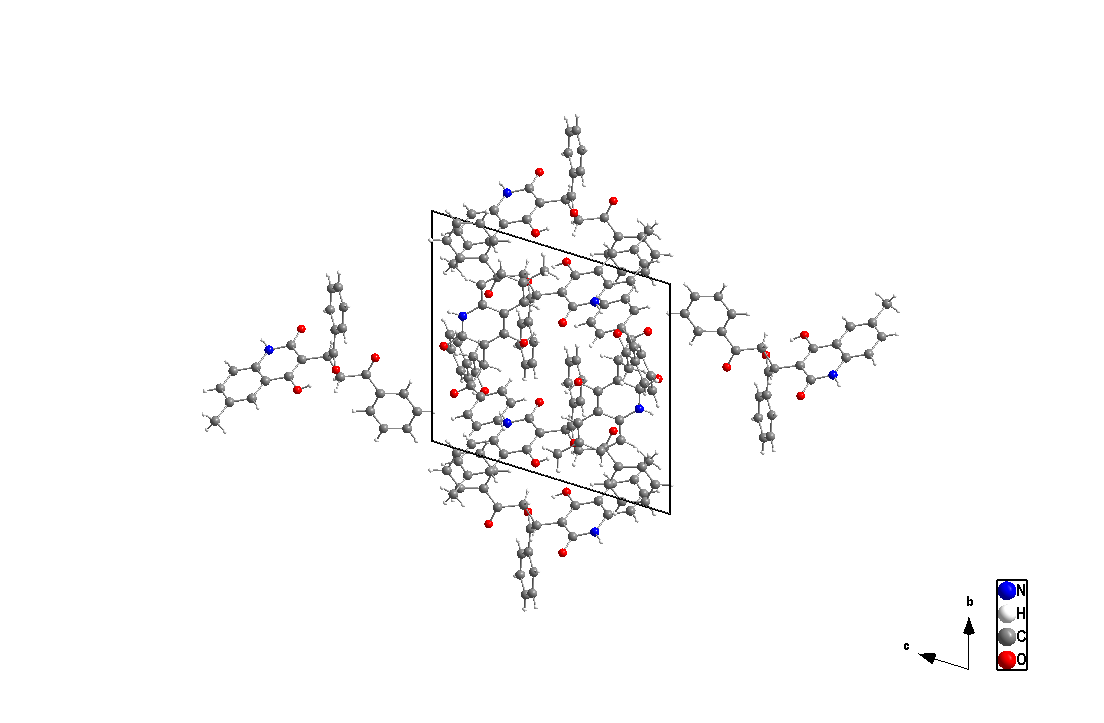


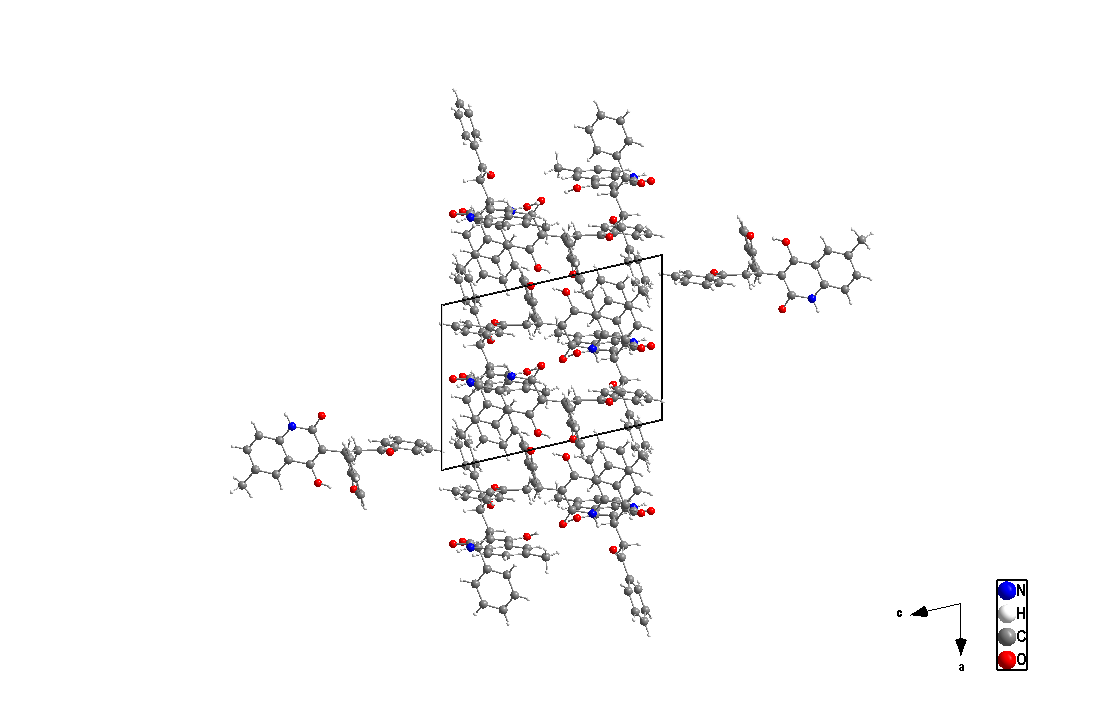


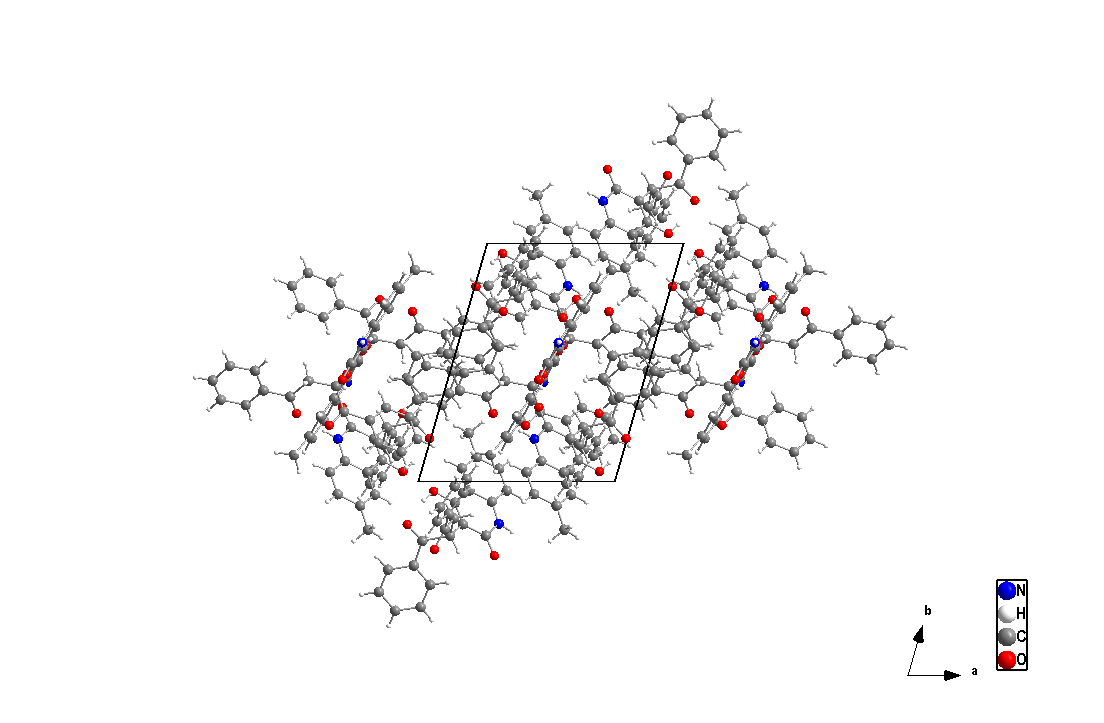


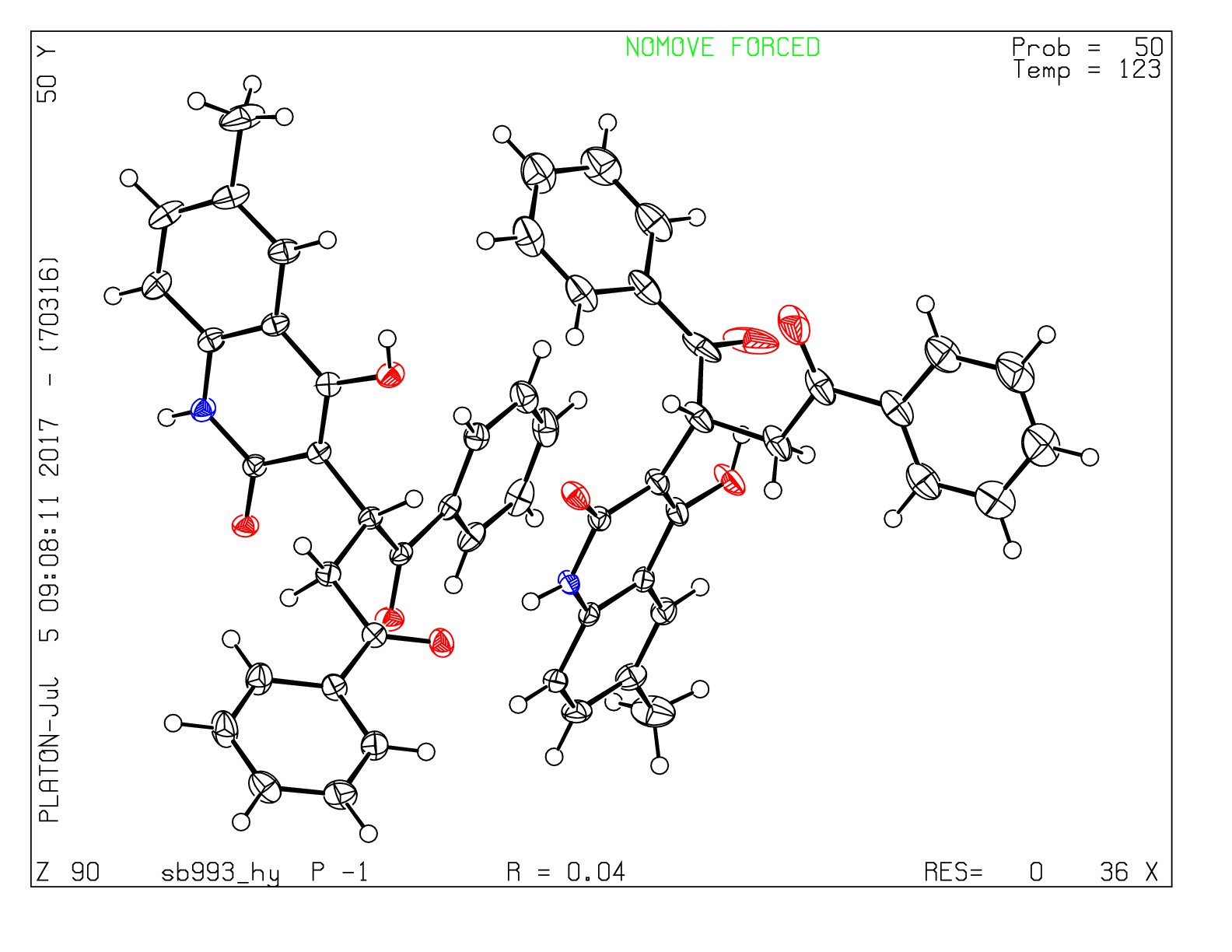


**2-(4-hydroxy-6-methyl-2-oxo-1,2-dihydroquinolin-3-yl)-1,4-diphenylbutane-1,4-dione – SB993_HY**

*Crystal data*

| C26H21NO4 | *Z* = 4 |
| --- | --- |
| *Mr* = 411.44 | *F*(000) = 864 |
| Triclinic, *P*-1 (no.2) | *D*x = 1.320 Mg m-3 |
| *a* = 11.1823 (3) Å | Cu *K*α radiation,  = 1.54178 Å |
| *b* = 14.3827 (4) Å | Cell parameters from 9863 reflections |
| *c* = 15.3460 (4) Å |  = 3.1–72.1° |
| α = 67.695 (1)° |  = 0.72 mm-1 |
| β = 70.666 (1)° | *T* = 123 K |
| γ = 68.389 (1)° | Blocks, colourless |
| *V* = 2069.77 (10) Å3 | 0.16 × 0.08 × 0.06 mm |

*Data collection*

| Bruker D8 VENTURE diffractometer with Photon100 detector | 8117 independent reflections |
| --- | --- |
| Radiation source: INCOATEC microfocus sealed tube | 6749 reflections with *I* > 2σ(*I*) |
| Detector resolution: 10.4167 pixels mm-1 | *R*int = 0.035 |
| rotation in  and , 1°, shutterless scans | max = 72.1°, min = 3.2° |
| Absorption correction: multi-scan  *SADABS* (Sheldrick, 2014) | *h* = -1313 |
| *T*min = 0.896, *T*max = 0.958 | *k* = -1717 |
| 28579 measured reflections | *l* = -1818 |

*Refinement*

| Refinement on *F*2 | Primary atom site location: structure-invariant direct methods |
| --- | --- |
| Least-squares matrix: full | Secondary atom site location: difference Fourier map |
| *R*[*F*2 > 2σ(*F*2)] = 0.043 | Hydrogen site location: difference Fourier map |
| *wR*(*F*2) = 0.112 | H atoms treated by a mixture of independent and constrained refinement |
| *S* = 1.01 | *w* = 1/[2(*F*o2) + (0.0508*P*)2 + 1.0588*P*]  where *P* = (*F*o2 + 2*F*c2)/3 |
| 8117 reflections | (Δ/σ)max < 0.001 |
| 577 parameters | max = 0.37 e Å-3 |
| 5 restraints | min = -0.50 e Å-3 |

**Computing details**

Data collection: *APEX3*; cell refinement: *APEX3*; data reduction: *SAINT*; program(s) used to solve structure: *SHELXS97*; program(s) used to refine structure: *SHELXL2014*/7 (Sheldrick, 2014); software used to prepare material for publication: *publCIF*.

*Special details*

| *Experimental*. dx = 40 mm, 1 deg., 13+1 runs, 2123 frames, 20/30/40 sec./frame |
| --- |
| *Geometry*. All esds (except the esd in the dihedral angle between two l.s. planes) are estimated using the full covariance matrix. The cell esds are taken into account individually in the estimation of esds in distances, angles and torsion angles; correlations between esds in cell parameters are only used when they are defined by crystal symmetry. An approximate (isotropic) treatment of cell esds is used for estimating esds involving l.s. planes. |

*Fractional atomic coordinates and isotropic or equivalent isotropic displacement parameters (Å2)*

|  | *x* | *y* | *z* | *U*iso*/*U*eq |
| --- | --- | --- | --- | --- |
| N1 | 0.47212 (12) | 0.82187 (9) | 0.31591 (9) | 0.0174 (2) |
| H1 | 0.5523 (16) | 0.7878 (14) | 0.2895 (14) | 0.029 (5)* |
| C2 | 0.42278 (14) | 0.77218 (11) | 0.40904 (10) | 0.0183 (3) |
| O2 | 0.49561 (10) | 0.68827 (8) | 0.45100 (7) | 0.0244 (2) |
| C3 | 0.28923 (15) | 0.82002 (12) | 0.45323 (11) | 0.0219 (3) |
| C4 | 0.21771 (15) | 0.91098 (12) | 0.39875 (11) | 0.0231 (3) |
| O4 | 0.09299 (12) | 0.95824 (9) | 0.43501 (9) | 0.0348 (3) |
| H4 | 0.055 (3) | 0.921 (2) | 0.4886 (17) | 0.102 (12)* |
| C4A | 0.27328 (15) | 0.96164 (11) | 0.30020 (10) | 0.0191 (3) |
| C5 | 0.20238 (16) | 1.05539 (12) | 0.24340 (11) | 0.0232 (3) |
| H5 | 0.1133 | 1.0869 | 0.2699 | 0.028* |
| C6 | 0.26045 (17) | 1.10206 (12) | 0.14991 (12) | 0.0264 (3) |
| C7 | 0.39219 (16) | 1.05373 (12) | 0.11184 (11) | 0.0249 (3) |
| H7 | 0.4332 | 1.0855 | 0.0477 | 0.030* |
| C8 | 0.46320 (15) | 0.96118 (12) | 0.16557 (11) | 0.0205 (3) |
| H8 | 0.5517 | 0.9292 | 0.1383 | 0.025* |
| C8A | 0.40369 (14) | 0.91473 (11) | 0.26055 (10) | 0.0174 (3) |
| C9 | 0.1850 (2) | 1.20304 (15) | 0.08938 (15) | 0.0427 (5) |
| H9A | 0.1588 | 1.1885 | 0.0420 | 0.064* |
| H9B | 0.2411 | 1.2505 | 0.0556 | 0.064* |
| H9C | 0.1061 | 1.2357 | 0.1312 | 0.064* |
| C10 | 0.10789 (16) | 0.75004 (15) | 0.58608 (12) | 0.0351 (4) |
| O10 | 0.00625 (13) | 0.81924 (11) | 0.59694 (14) | 0.0660 (6) |
| C11 | 0.24075 (16) | 0.77269 (13) | 0.56107 (11) | 0.0284 (4) |
| H11 | 0.3073 | 0.7047 | 0.5823 | 0.034* |
| C12 | 0.23549 (18) | 0.84481 (14) | 0.61512 (12) | 0.0337 (4) |
| H12A | 0.3215 | 0.8606 | 0.5941 | 0.040* |
| H12B | 0.1673 | 0.9116 | 0.5971 | 0.040* |
| C13 | 0.20501 (18) | 0.79897 (15) | 0.72412 (12) | 0.0348 (4) |
| O13 | 0.18128 (15) | 0.71442 (10) | 0.76169 (9) | 0.0457 (4) |
| C14 | 0.20314 (17) | 0.86267 (15) | 0.78259 (12) | 0.0327 (4) |
| C15 | 0.24605 (19) | 0.95225 (16) | 0.74110 (13) | 0.0387 (4) |
| H15 | 0.2787 | 0.9743 | 0.6732 | 0.046* |
| C16 | 0.2421 (2) | 1.01014 (18) | 0.79713 (15) | 0.0432 (5) |
| H16 | 0.2737 | 1.0704 | 0.7679 | 0.052* |
| C17 | 0.1919 (2) | 0.97951 (19) | 0.89588 (15) | 0.0477 (5) |
| H17 | 0.1881 | 1.0191 | 0.9346 | 0.057* |
| C18 | 0.1474 (2) | 0.8913 (2) | 0.93788 (14) | 0.0497 (5) |
| H18 | 0.1118 | 0.8709 | 1.0055 | 0.060* |
| C19 | 0.1542 (2) | 0.83227 (17) | 0.88211 (13) | 0.0404 (4) |
| H19 | 0.1253 | 0.7707 | 0.9119 | 0.048* |
| C20 | 0.10040 (16) | 0.64648 (15) | 0.59436 (12) | 0.0321 (4) |
| C21 | 0.21131 (17) | 0.56601 (16) | 0.57233 (13) | 0.0354 (4) |
| H21 | 0.2968 | 0.5759 | 0.5531 | 0.043* |
| C22 | 0.19700 (19) | 0.47175 (18) | 0.57848 (15) | 0.0414 (5) |
| H22 | 0.2727 | 0.4173 | 0.5633 | 0.050* |
| C23 | 0.0725 (2) | 0.45687 (19) | 0.60669 (16) | 0.0462 (5) |
| H23 | 0.0630 | 0.3924 | 0.6105 | 0.055* |
| C24 | -0.03820 (19) | 0.53576 (19) | 0.62935 (16) | 0.0468 (5) |
| H24 | -0.1233 | 0.5251 | 0.6489 | 0.056* |
| C25 | -0.02485 (18) | 0.62942 (17) | 0.62345 (14) | 0.0401 (5) |
| H25 | -0.1011 | 0.6832 | 0.6392 | 0.048* |
| N31 | 0.49153 (12) | 0.41642 (10) | 0.12944 (9) | 0.0199 (3) |
| H31 | 0.4879 (19) | 0.4149 (15) | 0.0744 (12) | 0.030 (5)* |
| C32 | 0.51501 (13) | 0.50386 (11) | 0.12774 (10) | 0.0173 (3) |
| O32 | 0.53702 (10) | 0.57244 (8) | 0.04942 (7) | 0.0212 (2) |
| C33 | 0.51352 (13) | 0.51196 (11) | 0.21923 (10) | 0.0165 (3) |
| C34 | 0.48170 (14) | 0.43771 (11) | 0.30309 (10) | 0.0183 (3) |
| O34 | 0.47874 (11) | 0.45091 (8) | 0.38601 (8) | 0.0233 (2) |
| H34 | 0.486 (2) | 0.3941 (15) | 0.4341 (13) | 0.057 (7)* |
| C34A | 0.44861 (14) | 0.34933 (11) | 0.30277 (11) | 0.0201 (3) |
| C35 | 0.40720 (16) | 0.27335 (12) | 0.38690 (12) | 0.0253 (3) |
| H35 | 0.3989 | 0.2793 | 0.4483 | 0.030* |
| C36 | 0.37830 (18) | 0.19011 (13) | 0.38190 (13) | 0.0309 (4) |
| C37 | 0.39271 (18) | 0.18158 (13) | 0.29086 (14) | 0.0327 (4) |
| H37 | 0.3759 | 0.1234 | 0.2868 | 0.039* |
| C38 | 0.43060 (17) | 0.25542 (12) | 0.20712 (13) | 0.0277 (3) |
| H38 | 0.4384 | 0.2487 | 0.1461 | 0.033* |
| C38A | 0.45753 (14) | 0.34057 (11) | 0.21256 (11) | 0.0206 (3) |
| C39 | 0.3284 (2) | 0.11120 (16) | 0.47252 (15) | 0.0467 (5) |
| H39A | 0.3396 | 0.1209 | 0.5289 | 0.070* |
| H39B | 0.3786 | 0.0403 | 0.4687 | 0.070* |
| H39C | 0.2347 | 0.1213 | 0.4786 | 0.070* |
| C40 | 0.48234 (14) | 0.70732 (11) | 0.16393 (10) | 0.0176 (3) |
| O40 | 0.53668 (11) | 0.76403 (8) | 0.09343 (8) | 0.0243 (2) |
| C41 | 0.55975 (14) | 0.59968 (11) | 0.21720 (10) | 0.0172 (3) |
| H41 | 0.5456 | 0.5983 | 0.2855 | 0.021* |
| C42 | 0.70728 (14) | 0.57766 (11) | 0.17334 (11) | 0.0201 (3) |
| H42A | 0.7497 | 0.5032 | 0.2034 | 0.024* |
| H42B | 0.7210 | 0.5875 | 0.1036 | 0.024* |
| C43 | 0.77730 (14) | 0.64341 (11) | 0.18366 (11) | 0.0196 (3) |
| O43 | 0.72020 (10) | 0.71417 (9) | 0.22092 (8) | 0.0252 (2) |
| C44 | 0.92323 (14) | 0.61773 (12) | 0.14665 (11) | 0.0209 (3) |
| C45 | 0.99427 (16) | 0.53461 (14) | 0.10823 (13) | 0.0291 (4) |
| H45 | 0.9496 | 0.4919 | 0.1045 | 0.035* |
| C46 | 1.13085 (16) | 0.51377 (15) | 0.07514 (14) | 0.0339 (4) |
| H46 | 1.1789 | 0.4569 | 0.0489 | 0.041* |
| C47 | 1.19612 (16) | 0.57535 (15) | 0.08047 (13) | 0.0311 (4) |
| H47 | 1.2889 | 0.5621 | 0.0564 | 0.037* |
| C48 | 1.12681 (16) | 0.65634 (15) | 0.12084 (13) | 0.0321 (4) |
| H48 | 1.1723 | 0.6972 | 0.1266 | 0.039* |
| C49 | 0.99133 (16) | 0.67806 (13) | 0.15283 (12) | 0.0267 (3) |
| H49 | 0.9441 | 0.7348 | 0.1793 | 0.032* |
| C50 | 0.33696 (15) | 0.74333 (11) | 0.20440 (11) | 0.0192 (3) |
| C51 | 0.27435 (15) | 0.69026 (12) | 0.29504 (11) | 0.0220 (3) |
| H51 | 0.3234 | 0.6269 | 0.3323 | 0.026* |
| C52 | 0.14066 (16) | 0.72988 (14) | 0.33089 (13) | 0.0299 (4) |
| H52 | 0.0984 | 0.6933 | 0.3924 | 0.036* |
| C53 | 0.06862 (17) | 0.82261 (15) | 0.27720 (14) | 0.0332 (4) |
| H53 | -0.0229 | 0.8495 | 0.3019 | 0.040* |
| C54 | 0.13029 (17) | 0.87614 (13) | 0.18741 (14) | 0.0310 (4) |
| H54 | 0.0810 | 0.9400 | 0.1509 | 0.037* |
| C55 | 0.26339 (16) | 0.83696 (12) | 0.15065 (12) | 0.0245 (3) |
| H55 | 0.3049 | 0.8737 | 0.0888 | 0.029* |

*Atomic displacement parameters (Å2)*

|  | *U*11 | *U*22 | *U*33 | *U*12 | *U*13 | *U*23 |
| --- | --- | --- | --- | --- | --- | --- |
| N1 | 0.0174 (6) | 0.0182 (6) | 0.0147 (6) | -0.0035 (5) | -0.0014 (5) | -0.0059 (5) |
| C2 | 0.0211 (7) | 0.0185 (7) | 0.0149 (7) | -0.0049 (6) | -0.0045 (5) | -0.0048 (5) |
| O2 | 0.0223 (5) | 0.0244 (5) | 0.0167 (5) | -0.0011 (4) | -0.0033 (4) | -0.0018 (4) |
| C3 | 0.0231 (8) | 0.0223 (7) | 0.0157 (7) | -0.0044 (6) | -0.0007 (6) | -0.0057 (6) |
| C4 | 0.0234 (8) | 0.0217 (7) | 0.0187 (7) | -0.0010 (6) | -0.0005 (6) | -0.0087 (6) |
| O4 | 0.0297 (6) | 0.0234 (6) | 0.0225 (6) | 0.0050 (5) | 0.0086 (5) | -0.0007 (5) |
| C4A | 0.0238 (7) | 0.0168 (7) | 0.0174 (7) | -0.0049 (6) | -0.0042 (6) | -0.0067 (6) |
| C5 | 0.0247 (8) | 0.0185 (7) | 0.0240 (8) | -0.0035 (6) | -0.0058 (6) | -0.0058 (6) |
| C6 | 0.0311 (8) | 0.0208 (7) | 0.0267 (8) | -0.0086 (6) | -0.0107 (7) | -0.0013 (6) |
| C7 | 0.0326 (8) | 0.0234 (8) | 0.0182 (7) | -0.0142 (7) | -0.0056 (6) | 0.0006 (6) |
| C8 | 0.0231 (7) | 0.0220 (7) | 0.0181 (7) | -0.0099 (6) | -0.0021 (6) | -0.0061 (6) |
| C8A | 0.0224 (7) | 0.0156 (6) | 0.0163 (7) | -0.0060 (5) | -0.0051 (5) | -0.0057 (5) |
| C9 | 0.0372 (10) | 0.0321 (10) | 0.0403 (11) | -0.0068 (8) | -0.0122 (8) | 0.0094 (8) |
| C10 | 0.0223 (8) | 0.0398 (10) | 0.0163 (7) | -0.0003 (7) | 0.0025 (6) | 0.0065 (7) |
| O10 | 0.0242 (7) | 0.0277 (7) | 0.0879 (12) | 0.0002 (6) | 0.0129 (7) | 0.0155 (7) |
| C11 | 0.0250 (8) | 0.0308 (8) | 0.0152 (7) | -0.0002 (7) | -0.0004 (6) | -0.0025 (6) |
| C12 | 0.0350 (9) | 0.0347 (9) | 0.0166 (8) | 0.0014 (7) | -0.0012 (7) | -0.0064 (7) |
| C13 | 0.0317 (9) | 0.0382 (10) | 0.0185 (8) | 0.0045 (7) | -0.0034 (7) | -0.0071 (7) |
| O13 | 0.0647 (10) | 0.0336 (7) | 0.0193 (6) | -0.0032 (6) | -0.0027 (6) | -0.0031 (5) |
| C14 | 0.0245 (8) | 0.0418 (10) | 0.0201 (8) | 0.0017 (7) | -0.0066 (6) | -0.0059 (7) |
| C15 | 0.0338 (10) | 0.0481 (11) | 0.0239 (9) | -0.0036 (8) | -0.0062 (7) | -0.0073 (8) |
| C16 | 0.0369 (10) | 0.0541 (12) | 0.0368 (10) | -0.0105 (9) | -0.0149 (8) | -0.0080 (9) |
| C17 | 0.0509 (12) | 0.0670 (14) | 0.0353 (10) | -0.0163 (11) | -0.0195 (9) | -0.0174 (10) |
| C18 | 0.0583 (13) | 0.0716 (15) | 0.0232 (9) | -0.0216 (12) | -0.0105 (9) | -0.0132 (10) |
| C19 | 0.0424 (11) | 0.0556 (12) | 0.0215 (9) | -0.0133 (9) | -0.0094 (8) | -0.0080 (8) |
| C20 | 0.0229 (8) | 0.0497 (11) | 0.0168 (7) | -0.0046 (7) | -0.0033 (6) | -0.0087 (7) |
| C21 | 0.0233 (8) | 0.0566 (12) | 0.0268 (9) | -0.0075 (8) | -0.0026 (7) | -0.0185 (8) |
| C22 | 0.0296 (9) | 0.0614 (13) | 0.0388 (10) | -0.0083 (9) | -0.0043 (8) | -0.0276 (10) |
| C23 | 0.0368 (11) | 0.0681 (14) | 0.0453 (12) | -0.0164 (10) | -0.0073 (9) | -0.0287 (11) |
| C24 | 0.0270 (9) | 0.0726 (15) | 0.0468 (12) | -0.0171 (10) | -0.0066 (8) | -0.0220 (11) |
| C25 | 0.0220 (9) | 0.0602 (13) | 0.0303 (9) | -0.0037 (8) | -0.0042 (7) | -0.0139 (9) |
| N31 | 0.0248 (6) | 0.0197 (6) | 0.0183 (6) | -0.0088 (5) | -0.0064 (5) | -0.0048 (5) |
| C32 | 0.0150 (6) | 0.0175 (7) | 0.0198 (7) | -0.0048 (5) | -0.0042 (5) | -0.0053 (6) |
| O32 | 0.0275 (6) | 0.0230 (5) | 0.0157 (5) | -0.0129 (4) | -0.0044 (4) | -0.0030 (4) |
| C33 | 0.0156 (6) | 0.0155 (6) | 0.0190 (7) | -0.0041 (5) | -0.0063 (5) | -0.0040 (5) |
| C34 | 0.0176 (7) | 0.0187 (7) | 0.0184 (7) | -0.0042 (5) | -0.0065 (5) | -0.0039 (6) |
| O34 | 0.0349 (6) | 0.0204 (5) | 0.0159 (5) | -0.0113 (5) | -0.0089 (4) | -0.0003 (4) |
| C34A | 0.0202 (7) | 0.0162 (7) | 0.0229 (7) | -0.0054 (5) | -0.0079 (6) | -0.0016 (6) |
| C35 | 0.0289 (8) | 0.0228 (8) | 0.0233 (8) | -0.0103 (6) | -0.0095 (6) | 0.0005 (6) |
| C36 | 0.0352 (9) | 0.0229 (8) | 0.0351 (9) | -0.0150 (7) | -0.0142 (7) | 0.0031 (7) |
| C37 | 0.0416 (10) | 0.0207 (8) | 0.0431 (10) | -0.0156 (7) | -0.0178 (8) | -0.0032 (7) |
| C38 | 0.0334 (9) | 0.0233 (8) | 0.0316 (9) | -0.0101 (7) | -0.0121 (7) | -0.0072 (7) |
| C38A | 0.0193 (7) | 0.0171 (7) | 0.0253 (8) | -0.0049 (6) | -0.0080 (6) | -0.0038 (6) |
| C39 | 0.0655 (14) | 0.0335 (10) | 0.0433 (12) | -0.0317 (10) | -0.0187 (10) | 0.0092 (9) |
| C40 | 0.0237 (7) | 0.0172 (7) | 0.0163 (7) | -0.0086 (6) | -0.0066 (5) | -0.0047 (6) |
| O40 | 0.0297 (6) | 0.0211 (5) | 0.0199 (5) | -0.0104 (4) | -0.0047 (4) | -0.0011 (4) |
| C41 | 0.0183 (7) | 0.0169 (7) | 0.0177 (7) | -0.0062 (5) | -0.0057 (5) | -0.0039 (5) |
| C42 | 0.0177 (7) | 0.0213 (7) | 0.0222 (7) | -0.0060 (6) | -0.0041 (6) | -0.0073 (6) |
| C43 | 0.0191 (7) | 0.0205 (7) | 0.0193 (7) | -0.0053 (6) | -0.0045 (5) | -0.0058 (6) |
| O43 | 0.0185 (5) | 0.0309 (6) | 0.0289 (6) | -0.0062 (4) | -0.0009 (4) | -0.0158 (5) |
| C44 | 0.0178 (7) | 0.0259 (7) | 0.0209 (7) | -0.0057 (6) | -0.0048 (6) | -0.0087 (6) |
| C45 | 0.0224 (8) | 0.0349 (9) | 0.0378 (9) | -0.0076 (7) | -0.0057 (7) | -0.0201 (8) |
| C46 | 0.0204 (8) | 0.0416 (10) | 0.0421 (10) | -0.0009 (7) | -0.0029 (7) | -0.0256 (8) |
| C47 | 0.0172 (7) | 0.0432 (10) | 0.0308 (9) | -0.0059 (7) | -0.0042 (6) | -0.0122 (8) |
| C48 | 0.0226 (8) | 0.0414 (10) | 0.0376 (10) | -0.0140 (7) | -0.0051 (7) | -0.0135 (8) |
| C49 | 0.0212 (8) | 0.0318 (8) | 0.0322 (9) | -0.0084 (6) | -0.0040 (6) | -0.0153 (7) |
| C50 | 0.0225 (7) | 0.0181 (7) | 0.0221 (7) | -0.0058 (6) | -0.0086 (6) | -0.0081 (6) |
| C51 | 0.0219 (7) | 0.0236 (7) | 0.0234 (7) | -0.0057 (6) | -0.0073 (6) | -0.0085 (6) |
| C52 | 0.0244 (8) | 0.0422 (10) | 0.0279 (8) | -0.0096 (7) | -0.0034 (6) | -0.0170 (7) |
| C53 | 0.0209 (8) | 0.0440 (10) | 0.0410 (10) | 0.0008 (7) | -0.0093 (7) | -0.0267 (8) |
| C54 | 0.0300 (9) | 0.0257 (8) | 0.0430 (10) | 0.0023 (7) | -0.0214 (8) | -0.0145 (7) |
| C55 | 0.0296 (8) | 0.0198 (7) | 0.0296 (8) | -0.0064 (6) | -0.0147 (7) | -0.0067 (6) |

*Geometric parameters (Å, º) for (sb993_hy)*

| N1—C2 | 1.3604 (19) | N31—C32 | 1.3656 (19) |
| --- | --- | --- | --- |
| N1—C8A | 1.3811 (18) | N31—C38A | 1.3747 (19) |
| N1—H1 | 0.891 (15) | N31—H31 | 0.868 (15) |
| C2—O2 | 1.2520 (18) | C32—O32 | 1.2494 (18) |
| C2—C3 | 1.446 (2) | C32—C33 | 1.447 (2) |
| C3—C4 | 1.375 (2) | C33—C34 | 1.363 (2) |
| C3—C11 | 1.523 (2) | C33—C41 | 1.5165 (18) |
| C4—O4 | 1.3385 (19) | C34—O34 | 1.3444 (18) |
| C4—C4A | 1.442 (2) | C34—C34A | 1.451 (2) |
| O4—H4 | 0.862 (17) | O34—H34 | 0.867 (16) |
| C4A—C8A | 1.397 (2) | C34A—C38A | 1.405 (2) |
| C4A—C5 | 1.409 (2) | C34A—C35 | 1.408 (2) |
| C5—C6 | 1.380 (2) | C35—C36 | 1.384 (2) |
| C5—H5 | 0.9500 | C35—H35 | 0.9500 |
| C6—C7 | 1.407 (2) | C36—C37 | 1.400 (3) |
| C6—C9 | 1.509 (2) | C36—C39 | 1.513 (2) |
| C7—C8 | 1.380 (2) | C37—C38 | 1.376 (2) |
| C7—H7 | 0.9500 | C37—H37 | 0.9500 |
| C8—C8A | 1.400 (2) | C38—C38A | 1.401 (2) |
| C8—H8 | 0.9500 | C38—H38 | 0.9500 |
| C9—H9A | 0.9800 | C39—H39A | 0.9800 |
| C9—H9B | 0.9800 | C39—H39B | 0.9800 |
| C9—H9C | 0.9800 | C39—H39C | 0.9800 |
| C10—O10 | 1.215 (2) | C40—O40 | 1.2104 (18) |
| C10—C20 | 1.478 (3) | C40—C50 | 1.501 (2) |
| C10—C11 | 1.531 (3) | C40—C41 | 1.5281 (19) |
| C11—C12 | 1.531 (3) | C41—C42 | 1.5220 (19) |
| C11—H11 | 1.0000 | C41—H41 | 1.0000 |
| C12—C13 | 1.517 (2) | C42—C43 | 1.508 (2) |
| C12—H12A | 0.9900 | C42—H42A | 0.9900 |
| C12—H12B | 0.9900 | C42—H42B | 0.9900 |
| C13—O13 | 1.219 (2) | C43—O43 | 1.2214 (18) |
| C13—C14 | 1.497 (3) | C43—C44 | 1.491 (2) |
| C14—C15 | 1.388 (3) | C44—C45 | 1.390 (2) |
| C14—C19 | 1.392 (2) | C44—C49 | 1.392 (2) |
| C15—C16 | 1.388 (3) | C45—C46 | 1.395 (2) |
| C15—H15 | 0.9500 | C45—H45 | 0.9500 |
| C16—C17 | 1.385 (3) | C46—C47 | 1.377 (3) |
| C16—H16 | 0.9500 | C46—H46 | 0.9500 |
| C17—C18 | 1.381 (3) | C47—C48 | 1.381 (3) |
| C17—H17 | 0.9500 | C47—H47 | 0.9500 |
| C18—C19 | 1.385 (3) | C48—C49 | 1.382 (2) |
| C18—H18 | 0.9500 | C48—H48 | 0.9500 |
| C19—H19 | 0.9500 | C49—H49 | 0.9500 |
| C20—C21 | 1.398 (3) | C50—C51 | 1.397 (2) |
| C20—C25 | 1.406 (3) | C50—C55 | 1.400 (2) |
| C21—C22 | 1.388 (3) | C51—C52 | 1.389 (2) |
| C21—H21 | 0.9500 | C51—H51 | 0.9500 |
| C22—C23 | 1.386 (3) | C52—C53 | 1.386 (3) |
| C22—H22 | 0.9500 | C52—H52 | 0.9500 |
| C23—C24 | 1.386 (3) | C53—C54 | 1.387 (3) |
| C23—H23 | 0.9500 | C53—H53 | 0.9500 |
| C24—C25 | 1.375 (3) | C54—C55 | 1.385 (2) |
| C24—H24 | 0.9500 | C54—H54 | 0.9500 |
| C25—H25 | 0.9500 | C55—H55 | 0.9500 |
|  |  |  |  |
| C2—N1—C8A | 124.66 (12) | C32—N31—C38A | 124.12 (13) |
| C2—N1—H1 | 115.3 (13) | C32—N31—H31 | 115.7 (13) |
| C8A—N1—H1 | 119.9 (13) | C38A—N31—H31 | 119.6 (13) |
| O2—C2—N1 | 118.12 (13) | O32—C32—N31 | 120.11 (13) |
| O2—C2—C3 | 124.21 (13) | O32—C32—C33 | 122.79 (13) |
| N1—C2—C3 | 117.67 (13) | N31—C32—C33 | 117.11 (12) |
| C4—C3—C2 | 118.79 (13) | C34—C33—C32 | 120.32 (13) |
| C4—C3—C11 | 123.36 (14) | C34—C33—C41 | 121.75 (13) |
| C2—C3—C11 | 117.53 (13) | C32—C33—C41 | 117.72 (12) |
| O4—C4—C3 | 121.67 (14) | O34—C34—C33 | 117.56 (13) |
| O4—C4—C4A | 116.30 (13) | O34—C34—C34A | 121.39 (13) |
| C3—C4—C4A | 122.03 (14) | C33—C34—C34A | 121.03 (13) |
| C4—O4—H4 | 115.2 (19) | C34—O34—H34 | 114.5 (15) |
| C8A—C4A—C5 | 119.32 (14) | C38A—C34A—C35 | 118.62 (14) |
| C8A—C4A—C4 | 117.67 (13) | C38A—C34A—C34 | 117.40 (13) |
| C5—C4A—C4 | 123.01 (14) | C35—C34A—C34 | 123.98 (14) |
| C6—C5—C4A | 120.97 (15) | C36—C35—C34A | 121.30 (15) |
| C6—C5—H5 | 119.5 | C36—C35—H35 | 119.3 |
| C4A—C5—H5 | 119.5 | C34A—C35—H35 | 119.3 |
| C5—C6—C7 | 118.64 (14) | C35—C36—C37 | 118.67 (15) |
| C5—C6—C9 | 121.00 (16) | C35—C36—C39 | 121.06 (17) |
| C7—C6—C9 | 120.36 (15) | C37—C36—C39 | 120.24 (16) |
| C8—C7—C6 | 121.50 (14) | C38—C37—C36 | 121.60 (15) |
| C8—C7—H7 | 119.3 | C38—C37—H37 | 119.2 |
| C6—C7—H7 | 119.3 | C36—C37—H37 | 119.2 |
| C7—C8—C8A | 119.44 (14) | C37—C38—C38A | 119.52 (16) |
| C7—C8—H8 | 120.3 | C37—C38—H38 | 120.2 |
| C8A—C8—H8 | 120.3 | C38A—C38—H38 | 120.2 |
| N1—C8A—C4A | 119.14 (13) | N31—C38A—C38 | 119.95 (14) |
| N1—C8A—C8 | 120.74 (13) | N31—C38A—C34A | 119.81 (13) |
| C4A—C8A—C8 | 120.12 (13) | C38—C38A—C34A | 120.24 (14) |
| C6—C9—H9A | 109.5 | C36—C39—H39A | 109.5 |
| C6—C9—H9B | 109.5 | C36—C39—H39B | 109.5 |
| H9A—C9—H9B | 109.5 | H39A—C39—H39B | 109.5 |
| C6—C9—H9C | 109.5 | C36—C39—H39C | 109.5 |
| H9A—C9—H9C | 109.5 | H39A—C39—H39C | 109.5 |
| H9B—C9—H9C | 109.5 | H39B—C39—H39C | 109.5 |
| O10—C10—C20 | 119.05 (18) | O40—C40—C50 | 120.54 (13) |
| O10—C10—C11 | 119.55 (19) | O40—C40—C41 | 121.43 (13) |
| C20—C10—C11 | 121.37 (15) | C50—C40—C41 | 117.97 (12) |
| C3—C11—C12 | 109.08 (14) | C33—C41—C42 | 108.43 (11) |
| C3—C11—C10 | 113.06 (14) | C33—C41—C40 | 112.40 (11) |
| C12—C11—C10 | 111.51 (14) | C42—C41—C40 | 112.16 (12) |
| C3—C11—H11 | 107.7 | C33—C41—H41 | 107.9 |
| C12—C11—H11 | 107.7 | C42—C41—H41 | 107.9 |
| C10—C11—H11 | 107.7 | C40—C41—H41 | 107.9 |
| C13—C12—C11 | 113.68 (16) | C43—C42—C41 | 116.40 (12) |
| C13—C12—H12A | 108.8 | C43—C42—H42A | 108.2 |
| C11—C12—H12A | 108.8 | C41—C42—H42A | 108.2 |
| C13—C12—H12B | 108.8 | C43—C42—H42B | 108.2 |
| C11—C12—H12B | 108.8 | C41—C42—H42B | 108.2 |
| H12A—C12—H12B | 107.7 | H42A—C42—H42B | 107.3 |
| O13—C13—C14 | 122.02 (16) | O43—C43—C44 | 120.14 (13) |
| O13—C13—C12 | 120.76 (17) | O43—C43—C42 | 123.09 (13) |
| C14—C13—C12 | 117.21 (17) | C44—C43—C42 | 116.77 (12) |
| C15—C14—C19 | 118.60 (18) | C45—C44—C49 | 118.73 (14) |
| C15—C14—C13 | 122.45 (16) | C45—C44—C43 | 122.65 (14) |
| C19—C14—C13 | 118.94 (18) | C49—C44—C43 | 118.60 (14) |
| C14—C15—C16 | 121.12 (18) | C44—C45—C46 | 120.24 (15) |
| C14—C15—H15 | 119.4 | C44—C45—H45 | 119.9 |
| C16—C15—H15 | 119.4 | C46—C45—H45 | 119.9 |
| C17—C16—C15 | 119.6 (2) | C47—C46—C45 | 120.15 (16) |
| C17—C16—H16 | 120.2 | C47—C46—H46 | 119.9 |
| C15—C16—H16 | 120.2 | C45—C46—H46 | 119.9 |
| C18—C17—C16 | 119.8 (2) | C46—C47—C48 | 119.99 (15) |
| C18—C17—H17 | 120.1 | C46—C47—H47 | 120.0 |
| C16—C17—H17 | 120.1 | C48—C47—H47 | 120.0 |
| C17—C18—C19 | 120.46 (19) | C47—C48—C49 | 120.11 (16) |
| C17—C18—H18 | 119.8 | C47—C48—H48 | 119.9 |
| C19—C18—H18 | 119.8 | C49—C48—H48 | 119.9 |
| C18—C19—C14 | 120.4 (2) | C48—C49—C44 | 120.74 (15) |
| C18—C19—H19 | 119.8 | C48—C49—H49 | 119.6 |
| C14—C19—H19 | 119.8 | C44—C49—H49 | 119.6 |
| C21—C20—C25 | 118.70 (19) | C51—C50—C55 | 119.17 (14) |
| C21—C20—C10 | 123.14 (16) | C51—C50—C40 | 122.66 (13) |
| C25—C20—C10 | 118.14 (17) | C55—C50—C40 | 118.09 (14) |
| C22—C21—C20 | 120.24 (17) | C52—C51—C50 | 120.21 (15) |
| C22—C21—H21 | 119.9 | C52—C51—H51 | 119.9 |
| C20—C21—H21 | 119.9 | C50—C51—H51 | 119.9 |
| C23—C22—C21 | 120.10 (19) | C53—C52—C51 | 120.23 (16) |
| C23—C22—H22 | 119.9 | C53—C52—H52 | 119.9 |
| C21—C22—H22 | 119.9 | C51—C52—H52 | 119.9 |
| C24—C23—C22 | 120.2 (2) | C52—C53—C54 | 119.90 (16) |
| C24—C23—H23 | 119.9 | C52—C53—H53 | 120.1 |
| C22—C23—H23 | 119.9 | C54—C53—H53 | 120.1 |
| C25—C24—C23 | 120.01 (18) | C55—C54—C53 | 120.36 (15) |
| C25—C24—H24 | 120.0 | C55—C54—H54 | 119.8 |
| C23—C24—H24 | 120.0 | C53—C54—H54 | 119.8 |
| C24—C25—C20 | 120.71 (18) | C54—C55—C50 | 120.14 (16) |
| C24—C25—H25 | 119.6 | C54—C55—H55 | 119.9 |
| C20—C25—H25 | 119.6 | C50—C55—H55 | 119.9 |
|  |  |  |  |
| C8A—N1—C2—O2 | -179.97 (13) | C38A—N31—C32—O32 | -175.33 (13) |
| C8A—N1—C2—C3 | 0.0 (2) | C38A—N31—C32—C33 | 4.9 (2) |
| O2—C2—C3—C4 | -178.22 (15) | O32—C32—C33—C34 | 176.32 (13) |
| N1—C2—C3—C4 | 1.9 (2) | N31—C32—C33—C34 | -4.0 (2) |
| O2—C2—C3—C11 | 8.1 (2) | O32—C32—C33—C41 | -8.9 (2) |
| N1—C2—C3—C11 | -171.79 (14) | N31—C32—C33—C41 | 170.84 (12) |
| C2—C3—C4—O4 | 179.00 (15) | C32—C33—C34—O34 | -178.34 (12) |
| C11—C3—C4—O4 | -7.7 (3) | C41—C33—C34—O34 | 7.1 (2) |
| C2—C3—C4—C4A | -2.1 (2) | C32—C33—C34—C34A | -0.1 (2) |
| C11—C3—C4—C4A | 171.20 (15) | C41—C33—C34—C34A | -174.63 (13) |
| O4—C4—C4A—C8A | 179.43 (14) | O34—C34—C34A—C38A | -178.50 (13) |
| C3—C4—C4A—C8A | 0.4 (2) | C33—C34—C34A—C38A | 3.3 (2) |
| O4—C4—C4A—C5 | -0.3 (2) | O34—C34—C34A—C35 | 2.2 (2) |
| C3—C4—C4A—C5 | -179.29 (15) | C33—C34—C34A—C35 | -176.02 (14) |
| C8A—C4A—C5—C6 | -0.9 (2) | C38A—C34A—C35—C36 | 1.4 (2) |
| C4—C4A—C5—C6 | 178.82 (15) | C34—C34A—C35—C36 | -179.32 (15) |
| C4A—C5—C6—C7 | 0.4 (2) | C34A—C35—C36—C37 | 0.9 (3) |
| C4A—C5—C6—C9 | -179.38 (16) | C34A—C35—C36—C39 | -177.43 (17) |
| C5—C6—C7—C8 | 0.4 (2) | C35—C36—C37—C38 | -2.1 (3) |
| C9—C6—C7—C8 | -179.76 (16) | C39—C36—C37—C38 | 176.22 (18) |
| C6—C7—C8—C8A | -0.8 (2) | C36—C37—C38—C38A | 1.0 (3) |
| C2—N1—C8A—C4A | -1.6 (2) | C32—N31—C38A—C38 | 177.65 (14) |
| C2—N1—C8A—C8 | 179.00 (13) | C32—N31—C38A—C34A | -1.7 (2) |
| C5—C4A—C8A—N1 | -178.89 (13) | C37—C38—C38A—N31 | -178.01 (15) |
| C4—C4A—C8A—N1 | 1.4 (2) | C37—C38—C38A—C34A | 1.3 (2) |
| C5—C4A—C8A—C8 | 0.5 (2) | C35—C34A—C38A—N31 | 176.86 (14) |
| C4—C4A—C8A—C8 | -179.22 (13) | C34—C34A—C38A—N31 | -2.5 (2) |
| C7—C8—C8A—N1 | 179.71 (13) | C35—C34A—C38A—C38 | -2.5 (2) |
| C7—C8—C8A—C4A | 0.3 (2) | C34—C34A—C38A—C38 | 178.16 (14) |
| C4—C3—C11—C12 | -67.6 (2) | C34—C33—C41—C42 | 106.57 (15) |
| C2—C3—C11—C12 | 105.78 (16) | C32—C33—C41—C42 | -68.14 (15) |
| C4—C3—C11—C10 | 57.1 (2) | C34—C33—C41—C40 | -128.86 (14) |
| C2—C3—C11—C10 | -129.53 (16) | C32—C33—C41—C40 | 56.43 (17) |
| O10—C10—C11—C3 | -85.1 (2) | O40—C40—C41—C33 | -120.30 (14) |
| C20—C10—C11—C3 | 92.99 (18) | C50—C40—C41—C33 | 62.58 (16) |
| O10—C10—C11—C12 | 38.3 (2) | O40—C40—C41—C42 | 2.19 (19) |
| C20—C10—C11—C12 | -143.66 (15) | C50—C40—C41—C42 | -174.93 (12) |
| C3—C11—C12—C13 | -172.72 (14) | C33—C41—C42—C43 | -167.76 (12) |
| C10—C11—C12—C13 | 61.70 (19) | C40—C41—C42—C43 | 67.52 (16) |
| C11—C12—C13—O13 | -3.4 (2) | C41—C42—C43—O43 | -3.3 (2) |
| C11—C12—C13—C14 | 177.70 (15) | C41—C42—C43—C44 | 176.12 (13) |
| O13—C13—C14—C15 | 171.62 (18) | O43—C43—C44—C45 | 176.52 (15) |
| C12—C13—C14—C15 | -9.4 (2) | C42—C43—C44—C45 | -2.9 (2) |
| O13—C13—C14—C19 | -9.7 (3) | O43—C43—C44—C49 | -2.0 (2) |
| C12—C13—C14—C19 | 169.27 (16) | C42—C43—C44—C49 | 178.60 (14) |
| C19—C14—C15—C16 | 0.8 (3) | C49—C44—C45—C46 | -1.1 (3) |
| C13—C14—C15—C16 | 179.49 (17) | C43—C44—C45—C46 | -179.63 (16) |
| C14—C15—C16—C17 | -1.4 (3) | C44—C45—C46—C47 | 0.1 (3) |
| C15—C16—C17—C18 | 0.6 (3) | C45—C46—C47—C48 | 1.6 (3) |
| C16—C17—C18—C19 | 0.9 (3) | C46—C47—C48—C49 | -2.3 (3) |
| C17—C18—C19—C14 | -1.6 (3) | C47—C48—C49—C44 | 1.3 (3) |
| C15—C14—C19—C18 | 0.7 (3) | C45—C44—C49—C48 | 0.4 (2) |
| C13—C14—C19—C18 | -178.03 (18) | C43—C44—C49—C48 | 179.03 (15) |
| O10—C10—C20—C21 | 172.48 (18) | O40—C40—C50—C51 | -167.34 (14) |
| C11—C10—C20—C21 | -5.6 (2) | C41—C40—C50—C51 | 9.8 (2) |
| O10—C10—C20—C25 | -5.8 (3) | O40—C40—C50—C55 | 9.2 (2) |
| C11—C10—C20—C25 | 176.09 (15) | C41—C40—C50—C55 | -173.61 (12) |
| C25—C20—C21—C22 | 0.5 (3) | C55—C50—C51—C52 | 0.3 (2) |
| C10—C20—C21—C22 | -177.78 (17) | C40—C50—C51—C52 | 176.88 (13) |
| C20—C21—C22—C23 | -0.1 (3) | C50—C51—C52—C53 | -0.3 (2) |
| C21—C22—C23—C24 | -0.3 (3) | C51—C52—C53—C54 | -0.1 (3) |
| C22—C23—C24—C25 | 0.3 (3) | C52—C53—C54—C55 | 0.6 (3) |
| C23—C24—C25—C20 | 0.1 (3) | C53—C54—C55—C50 | -0.6 (2) |
| C21—C20—C25—C24 | -0.6 (3) | C51—C50—C55—C54 | 0.1 (2) |
| C10—C20—C25—C24 | 177.85 (17) | C40—C50—C55—C54 | -176.58 (13) |

*Hydrogen-bond geometry (Å, º) for (sb993_hy)*

| *D*—H···*A* | *D*—H | H···*A* | *D*···*A* | *D*—H···*A* |
| --- | --- | --- | --- | --- |
| N1—H1···O43 | 0.89 (2) | 1.96 (2) | 2.8445 (16) | 174 (2) |
| O4—H4···O10 | 0.86 (2) | 1.83 (2) | 2.6785 (18) | 168 (3) |
| C11—H11···O34i | 1.00 | 2.64 | 3.6215 (19) | 167 |
| C12—H12*B*···O4 | 0.99 | 2.65 | 3.228 (2) | 118 |
| N31—H31···O32ii | 0.87 (2) | 1.94 (2) | 2.8054 (16) | 173 (2) |
| O34—H34···O2i | 0.87 (2) | 1.74 (2) | 2.5685 (14) | 159 (2) |
| C42—H42*B*···O32 | 0.99 | 2.56 | 3.1397 (18) | 117 |

Symmetry codes: (i) -*x*+1, -*y*+1, -*z*+1; (ii) -*x*+1, -*y*+1, -*z*.

Document origin: *publCIF* [Westrip, S. P. (2010). *J. Apply. Cryst.*, **43**, 920-925].
